# Stepwise Evolution of Coral Biomineralization Revealed with Genome-Wide Proteomics and Transcriptomics

**DOI:** 10.1371/journal.pone.0156424

**Published:** 2016-06-02

**Authors:** Takeshi Takeuchi, Lixy Yamada, Chuya Shinzato, Hitoshi Sawada, Noriyuki Satoh

**Affiliations:** 1 Marine Genomics Unit, Okinawa Institute of Science and Technology Graduate University, Onna, Okinawa, 904–0495, Japan; 2 Sugashima Marine Biological Laboratory, Graduate School of Science, Nagoya University, Sugashima, Toba, 517–0004, Japan; King Abdullah University of Science and Technology, SAUDI ARABIA

## Abstract

Despite the importance of stony corals in many research fields related to global issues, such as marine ecology, climate change, paleoclimatogy, and metazoan evolution, very little is known about the evolutionary origin of coral skeleton formation. In order to investigate the evolution of coral biomineralization, we have identified skeletal organic matrix proteins (SOMPs) in the skeletal proteome of the scleractinian coral, *Acropora digitifera*, for which large genomic and transcriptomic datasets are available. Scrupulous gene annotation was conducted based on comparisons of functional domain structures among metazoans. We found that SOMPs include not only coral-specific proteins, but also protein families that are widely conserved among cnidarians and other metazoans. We also identified several conserved transmembrane proteins in the skeletal proteome. Gene expression analysis revealed that expression of these conserved genes continues throughout development. Therefore, these genes are involved not only skeleton formation, but also in basic cellular functions, such as cell-cell interaction and signaling. On the other hand, genes encoding coral-specific proteins, including extracellular matrix domain-containing proteins, galaxins, and acidic proteins, were prominently expressed in post-settlement stages, indicating their role in skeleton formation. Taken together, the process of coral skeleton formation is hypothesized as: 1) formation of initial extracellular matrix between epithelial cells and substrate, employing pre-existing transmembrane proteins; 2) additional extracellular matrix formation using novel proteins that have emerged by domain shuffling and rapid molecular evolution and; 3) calcification controlled by coral-specific SOMPs.

## Introduction

Reef-building corals (Cnidaria, Anthozoa, Scleractinia) produce huge masses of calcium carbonate structures in the ocean [1]. Coral reefs provide habitats for one-third of all described marine species [2]. Nevertheless corals face a range of anthropogenic challenges, including ocean acidification and increasing seawater temperatures [3]. Loss of corals results in loss of entire reef ecosystems.

Scleractinian corals, which appear in the fossil record as early as the Triassic [4–6], are the dominant reef builders in shallow tropical and subtropical oceans. Scleractinians were derived from soft-bodied animals that did not possess calcified exoskeletons [6–8]. Coral calcification is a striking example of biomineralization, a complicated process including crystal nucleation and crystal growth. Polymorphism and microstructure of the biomineral are controlled by the organism itself [9, 10]. The enigma is how corals regulate such an intricate process and how such a versatile mechanism arose.

The coral skeleton contains organic macromolecules such as proteins [11, 12], phospholipids [13], and polysaccharides [14, 15]. It is widely accepted that such components, collectively termed the organic matrix, play a major role in the biomineralization process [9, 16–18]. The mineralizing front of the skeletal surface is adjacent to the aboral epidermal cell layer, called calicoblastic ectoderm [19]. Micro-scale observations using SEM and TEM show that an organic fibrous substance is present between the calicoblastic ectoderm and the skeleton [20–22]. Immunohistochemical studies have revealed that the organic component of the skeleton is produced by the calicoblastic ectoderm [23, 24]. In addition, *in vitro* experiments have demonstrated that the organic matrix extracted from the skeleton could affect the polymorphism and morphology of deposited calcium carbonate crystals [25]. Taken together, these previous observations indicate that the organic matrix provided by calicoblastic ectoderm plays a central role in coral skeleton formation, by generating the scaffold for calcification and by controlling the crystallization process in the extracellular space. Therefore, to uncover molecular mechanisms of coral skeleton formation, it is essential to identify all components in the organic matrix.

Recently, a number of coral skeletal organic matrix proteins or SOMPs have been identified proteomically. Drake *et al*. [26] reported 36 proteins from the *Stylophora pistillata* skeleton, using LC/MS/MS protein sequencing and a draft genome assembly of the *S*. *pistillata* genome. They identified adhesion proteins, collagens, carbonic anhydrases, and acidic proteins. They also listed some cellular components including actins and tubulin, although whether these proteins are real SOMPs or merely contaminants from cells in the sample remains a matter of debate [24, 27, 28]. Ramos-Silva *et al*. [29] identified 36 SOMPs in the *Acropora millepora* skeletal proteome and BLAST searches showed that many of them are homologous or similar to proteins of non-calcifying cnidarians, while a few are unique to scleractinians. They also claimed that other SOMPs with multiple domains of extracellular matrix proteins arose by domain shuffling. Despite these efforts, the evolutionary origin of all SOMPs and their roles in skeleton formation are unclear because many coral SOMPs are ambiguously annotated, based on partial sequence similarities to known proteins. Moreover, how all these genes evolved to optimize their functions for the entirely novel process of skeleton formation is unknown.

In addition, the role of SOMPs during coral development has been poorly studied. Skeleton formation between calicoblastic ectoderm of settled polyps and the substrate occurs soon after larval settlement [30, 31], and microscopic observation showed that microstructure of the calcareous elements in the polyp stage is different from that of adult skeleton [31–33]. Based on these observations, expression of SOMP genes is thought to start after larval settlement, and genes responsible for larval skeleton formation are not identical to those of adults. In order to test this hypothesis, gene expression profiling throughout development is essential.

In this paper we propose potential molecular functions of coral SOMP and hypothesize their evolutionary origins. First, we report 30 SOMPs of a scleractinian coral, *Acropora digitifera*, for which whole genomic and comprehensive transcriptome sequences are available [34]. LC/MS/MS analysis of the *A*. *digitifera* skeletal proteome provides direct evidence that the proteins are related to the coral skeleton formation process. Next, for the first time, we analyzed the expression of all SOMP genes during development using RNA-seq, and providing valuable information for inferring protein functions in both larvae and adults. Furthermore, we conducted thorough annotation of SOMPs by comparing functional domain architecture among a wide range of metazoans for which genome sequences are available. Genome and transcriptome sequences allowed us to retrieve many full-length protein sequences that are necessary for accurate annotation. In case the comparison of functional domain architecture proved insufficient to annotate *A*. *digitifera* SOMP genes, phylogenetic analysis was also performed. In conclusion, we hypothesize the evolutionary means by which corals acquired the ability to form mineralized skeletons.

## Materials and Methods

### Transcriptome analysis

Total RNA of various embryonic and larval developmental stages (eggs, blastulae, gastrulae, swimming larvae, and metamorphosing larvae) and adult *A*. *digitifera* was isolated [34]. Total RNA was then fragmented into about 200 bp lengths and an RNA-seq library was prepared using a TruSeq RNA Sample Prep Kit v2 (Illumina). Libraries were sequenced (100 bp paired-end reads) using an Illumina GAIIx platform. Sequence data were mapped to the *A*. *digitifera* genome using TopHat ver. 2.0.8b [35]. Differential gene expression analysis and calculations of FPKM (Fragments Per Kilobase of exon per Million mapped fragments) for each *A*. *digitifera* gene model were performed using Cufflinks ver. 2.1.1 [36] with default parameters.

### Proteomic analysis

The skeleton used was from the same *A*. *digitifera* colony from which the genome was decoded [34]. Specimens were stirred in 12.5% NaClO and washed with H_2_O several times to remove soft tissue. The skeleton was mechanically crushed and ground into fine powder, and treated with NaClO again to remove remaining contaminants. The sample was washed in MilliQ water and then air-dried. The powder (10g) was decalcified with 1M acetic acid overnight and the de-calcified solution was centrifuged to separate insoluble material and solution. The insoluble pellet or acid insoluble matrix (AIM) was washed with MilliQ water several times. Then, the pellet was suspended in solubilization buffer (1% SDS, 10mM DTT, 50mM Tris-HCl (pH 8.0)). The acid soluble matrix (ASM) was precipitated from the soluble fraction using chloroform/methanol precipitation. Briefly, four volumes of methanol, one volume of chloroform, and three volumes of MilliQ water were added to one volume of soluble fraction. After mixing by vortexing, the sample was centrifuged at 14,000g, at 4°C for 5 min. The top, aqueous layer was removed. Then four volumes of methanol were added and centrifuged at 14,000g, 4°C for 5 min. The methanol was removed by pipetting and then the sample was air-dried. Finally, the sample was suspended in solubilization buffer.

ASM and AIM samples were dissolved in sample loading buffer for SDS-PAGE. Proteins were separated in a 10%-20% gradient gel, visualized with silver staining and CBB staining, and size-fractionated by dividing the gel horizontally into 16 pieces. Each piece of the gel was digested with trypsin for LC/MS/MS analysis as described previously [37]. Briefly, peptide digests were analyzed using a capillary liquid chromatography system (Ultima3000; DIONEX) connected online to a mass spectrometer (LTQ-XL, Thermo Scientific). Raw spectra were processed using SEQUEST software to extract peak lists [38]. Resulting peak lists were analyzed using an in-house MASCOT (ver. 2.3.2) server against *A*. *digitifera* sequences included in gene models and also against RNA-seq sequences, which were retrieved from MarinegenomicsDB (http://marinegenomics.oist.jp/genomes/gallery). The false discovery rate was set to 0.05. Proteins with a spectral count of more than 2 in each sample were treated as identified in this study.

### Characterization and annotation of SOMP sequences

Protein sequences identified through proteomic analysis were analyzed using the InterProScan 5.3 platform [39] in order to find conserved functional domains. Signal peptide prediction was conducted with SignalP 4.0 [40], and non-classical protein secretion information was retrieved with SecretomeP 2.0 [41]. Proteins with high NN-scores (0.5 or higher) were regarded as possible extracellular proteins. Transmembrane domains were assessed with TMHMM 2.0 software [42]. Phosphorylation sites of amino acid sequences were predicted using NetPhos 2.0 Server [43]. All of these programs were run using default settings and thresholds.

In order to search for proteins with domain architectures similar to those of other metazoans, functional domain searching, described above, was also applied to a wide range of animals for which whole genome sequences are available. Genomic data used are listed in S1 Table.

For phylogenetic analysis, sequences of diverse metazoan animals were retrieved from public databases as described in S2 Table. Multiple sequence alignments of amino acid sequences were made with MUSCLE 3.8 [44] with default parameters, and visualized with Jalview version 2.8.2 [45]. The alignment was prepared with Gblocks version 0.91b [46] under default settings in order to remove poorly aligned positions if needed. Then, phylogenetic analysis was conducted using the maximum likelihood (ML) method in RAxML 8.1.3 [47]. The best-fit model of amino acid evolution for the tree was selected using ProteinModelSelection.pl script provided in the RAxML package. Neighbor-joining (NJ) trees were also obtained using ClustalW version 2.1 [48]. Reliability of the topology was checked by bootstrap analysis on the basis of 1,000 replicates for the ML and NJ methods. This dataset was also analyzed using PhyloBayes 3.3b [49] with the site heterogeneous mixture CAT model and two independent Markov chains. Convergence between the two chains was confirmed by comparing the frequency of their bipartitions.

## Results

### Identification of coral skeletal matrix proteins

**Fig 1:**
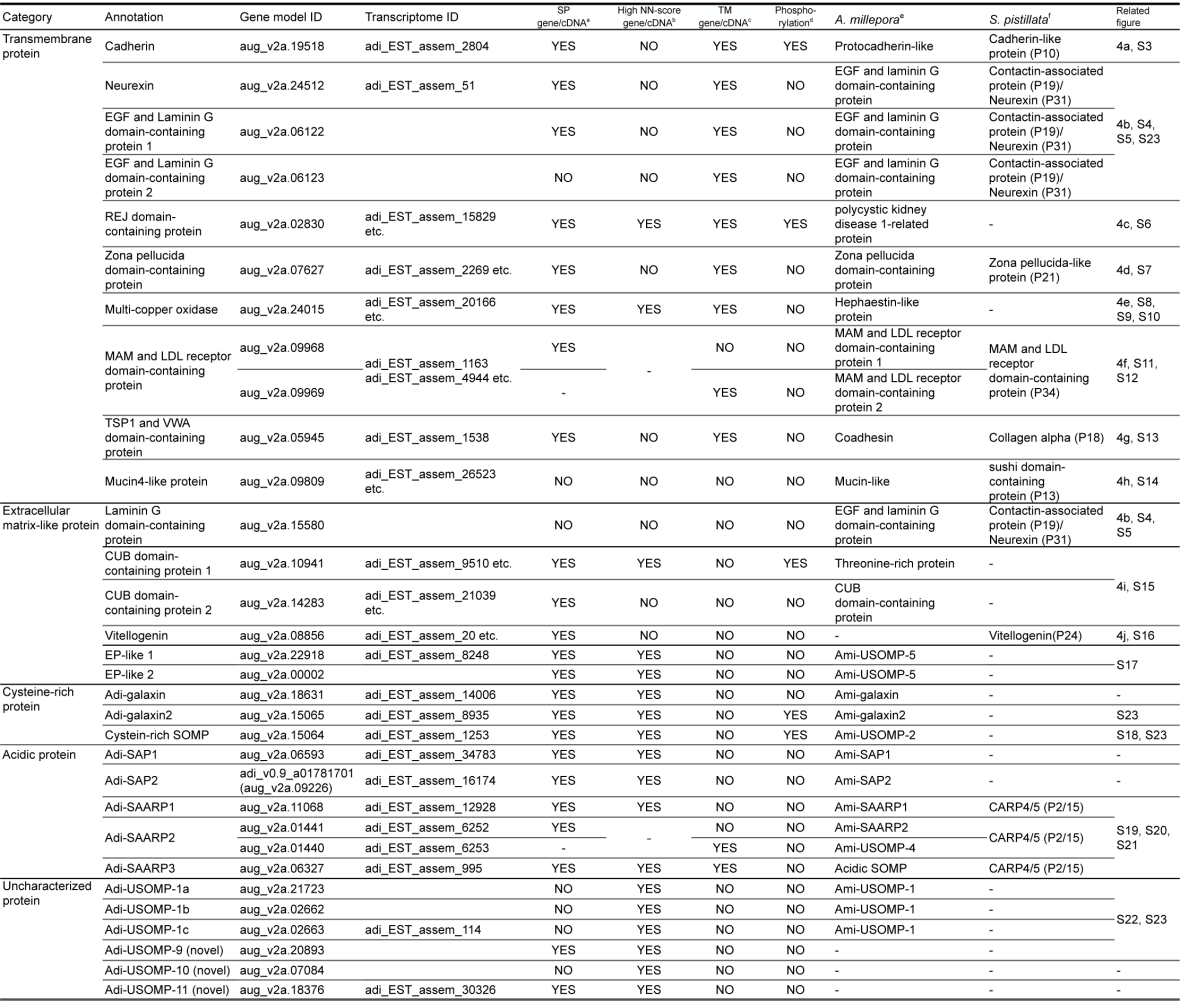
List of skeletal organic matrix proteins in *A*. *digitifera*. See also S3 Table. a: The presence of a signal peptide at the N-terminus predicted by SignalP [40]. b: Possible extracellular localization predicted by SecretomeP [41]. c: Presence of a transmembrane domain predicted by TMHMM [42]. d: Phosphorylation detected by MS/MS. e: Orthologous protein in the *A*. *millepora* proteome [29]. f: Orthologous protein in the *S*. *pistillata* proteome [26].

In order to identify coral skeleton matrix proteins, *A*. *digitifera* gene models version 2 and a transcriptome database [34] were searched. Fig 1 and S3 Table list *A*. *digitifera* SOMPs. Proteins from ASM and AIM were separated by gel electrophoresis (S1 Fig) and their analysis after tryptic digestion allowed us to identify 30 proteins (Fig 1, S3 Table). The presence of all these SOMPs in the skeletal proteome was supported by more than one unique peptide identified by LC/MS/MS analysis (S3 Table). All proteins found in ASM were also detected in AIM. Comparisons among three coral skeleton proteomes (*A*. *digitifera*, *A*. *millepora*, and *S*. *pistillata*) showed that 27 *A*. *digitifera* proteins had significant sequence similarity to skeletal matrix proteins of at least one of the other two coral species, and the remaining three proteins (USOMP -9 to -11) were newly identified in the *A*. *digitifera* skeletal proteome (S2 Fig). Twelve SOMPs were conserved among three species, while fourteen proteins were shared between *Acropora* species but not found in *S*. *pistillata*. One protein (vitellogenin) was detected from the *A*. *digitifera* and *S*. *pistillata* proteomes, but not from *A*. *millepora*. Four SOMPs of *A*. *digitifera* were not described in the proteome of the closely related *A*. *millepora*, while 12 SOMPs in the *A*. *millepora* proteome have not been detected in the *A*. *digitifera* skeleton.

### Protein localization and classification

The *in vivo* localization of proteins predicted using SignalP, SecretomeP, and TMHMM software showed that almost all of the identified proteins are either secretory or transmembrane proteins (Fig 1, S3 Table). Sixteen SOMPs have been categorized as “Transmembrane” or “Extracellular matrix-like” based on their predicted localization. Both the “Cysteine-rich” proteins and “Acidic” proteins are secretory. The former are rich in cysteine residues while the latter have high percentages of aspartic acid and a signal peptide; two of them also contain a transmembrane domain near the N-terminus. Finally, six secretory SOMPs are called “Uncharacterized” after Ramos-Silva *et al*. [29], since they do not have any conventional conserved domains.

### Gene expression profile

**Fig 2:**
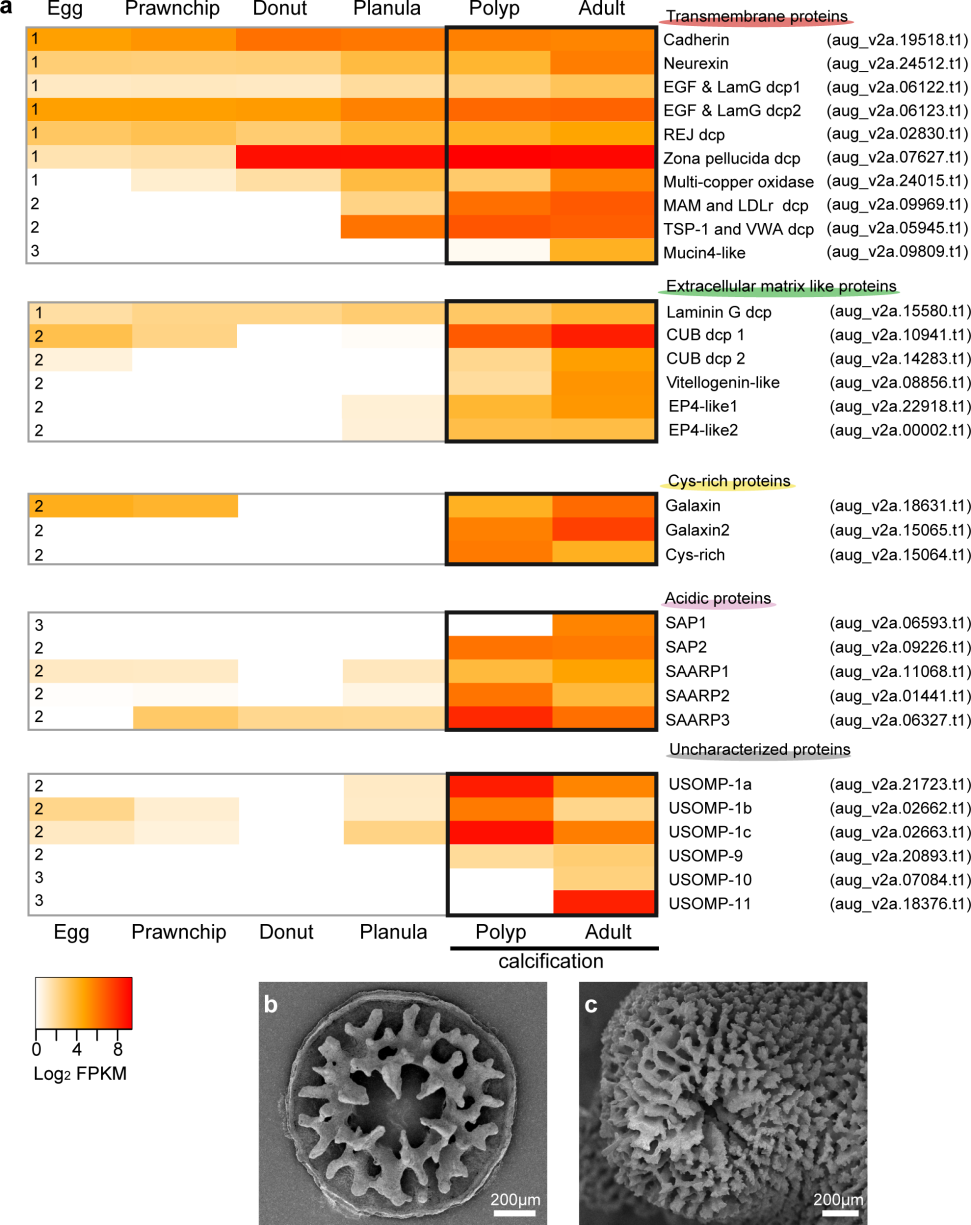
Gene expression patterns of SOMPs and SEM images of coral skeletons of polyp and adult stages. (a) Heatmap of SOMP gene expression at different developmental stages. Three expression patterns are indicated at the left: continuous expression before and during calcification (Pattern 1), strong expression commencing during the planula or polyp stages (Pattern 2), and exclusive expression in adult stage (Pattern 3). The color key represents FPKM of normalized log_2_ transformed counts. Orange to red color intensity indicates higher gene expression. Polyp and adult stages, in which corals generate calcium carbonate skeletons, are boxed in black. (b, c) SEM images of polyp (b) and adult (c) skeletons. The individual polyp used here was fixed seven days after settlement. Soft tissue of each sample was removed with 10% NaOH. After settlement, the disk-like structure was deposited onto the substrate. Then, a network structure similar to that of an adult skeleton began to form.

RNA-seq data obtained from embryonic and larval stages (e.g., egg, blastula, gastrula, planula, and polyp) and from adults included 29,446,547 read pairs for egg, 27,117,706 for blastula, 29,692,832 for gastrula, 29,400,663 for planula, 29,385,101 for polyp and 26,597,707 for adult. Photographs of *Acropora digitifera* developmental stages are shown in Fig 1 of Shinzato *et al*. [34]. Polyp and adult RNA libraries correspond to stages in which this species forms a calcareous skeleton (Fig 2). Gene expression analysis using RNA-seq showed three expression patterns. First, eight genes encoding cadherin, neurexin, EGF and laminin G domain-containing proteins (dcps), REJ dcp, zona pellucida dcp, multi-copper oxidase (MCO), and laminin G dcp, were expressed in earlier developmental stages before skeleton formation, as well as during polyp and adult stages (Pattern 1). Second, 18 genes that encode MAM and LDLr dcp, TSP and VWA dcp, CUB dcps, vitellogenin-like protein, EP-like proteins, galaxins, Cys-rich protein, SAP2, SAARPs, USOMP-1, and 9 began to be expressed in the planula or polyp stage (Pattern 2). Third, four genes (mucin4-like, SAP-1, USOMP-10, and -11) were expressed only during the adult stage (Pattern 3).

### Annotation of *Acropora digitifera* SOMPs

**Fig 3:**
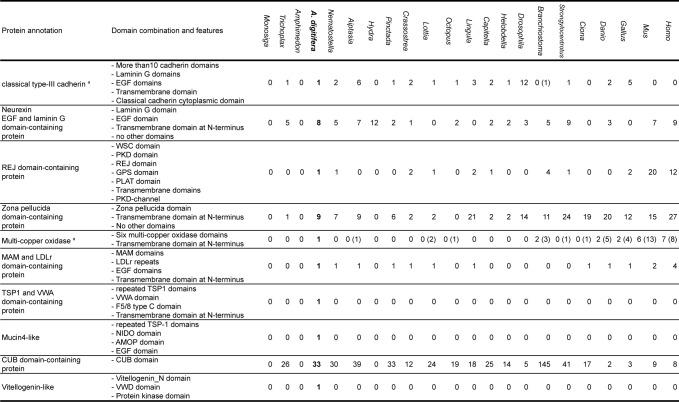
Number of proteins with specific domain architecture encoded in animal genomes. a: Figures in parentheses indicate number of proteins without transmembrane domain.

**Fig 4:**
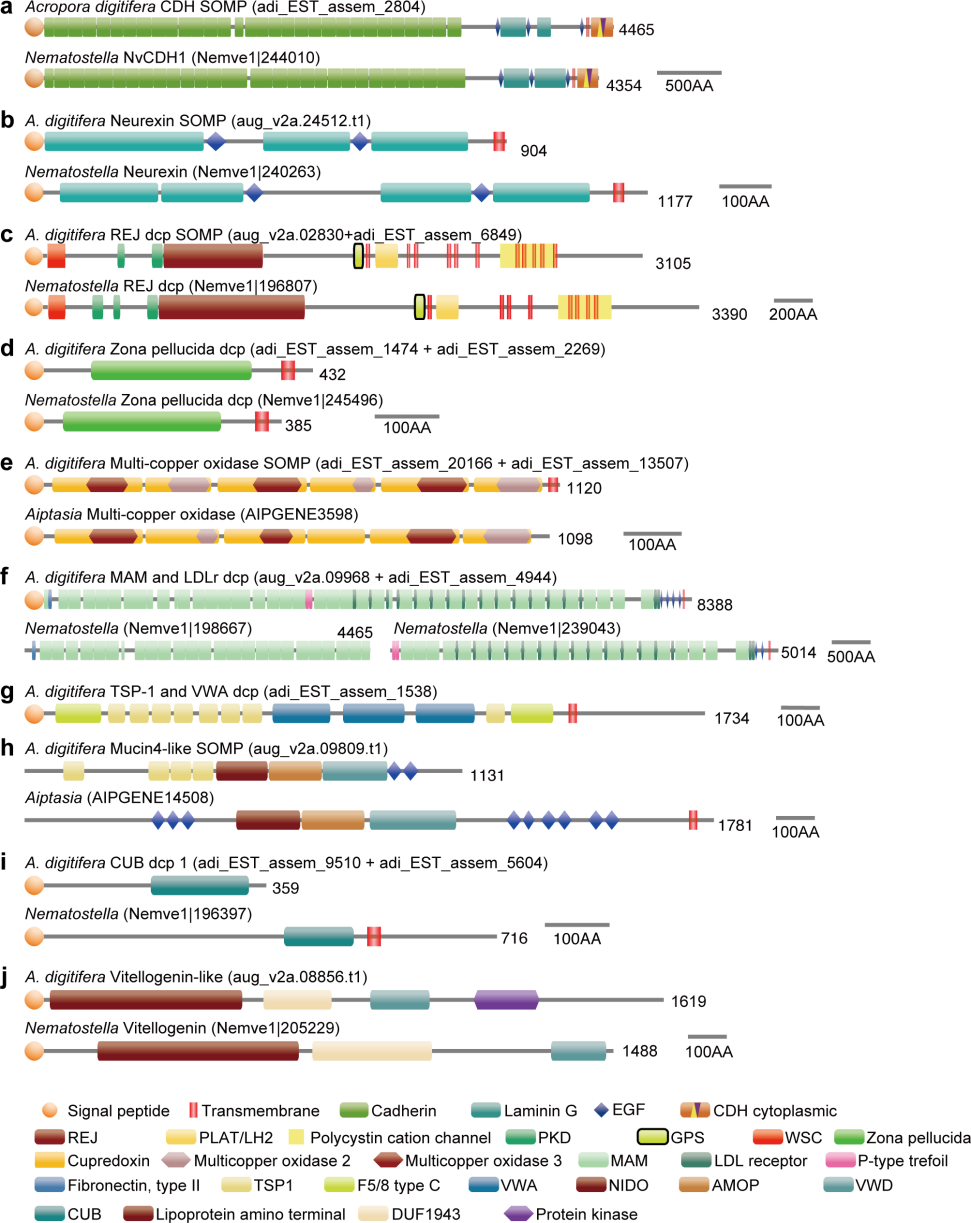
Domain architectures of SOMPs from *Acropora digitifera* and sea anemone proteins similar to the SOMPs. (a) Cadherin. (b) Neurexin. (c) REJ domain-containing protein (dcp). (d) Zona pellucida dcp. (e) Multi-copper oxidase. (f) MAM and LDL receptor dcp. (g) TSP-1 and VWA dcp. (h) Mucin4-like protein. (i) CUB dcp. (j) Vitellogenin-like protein. Lengths of amino acid sequences are shown at the right.

We searched functional domains in SOMP sequences and compared them to proteins having the same or similar domain architecture encoded in other metazoan animal genomes (Fig 3). Domain architectures of transmembrane and extracellular matrix-like SOMPs are shown in Fig 4. Other cnidarian (*Nematostella* and *Aiptasia*) proteins with similar domain architecture are also depicted for comparison. Most of the domain architectures of transmembrane and extracellular matrix-like SOMPs, except TSP-1 and VWA dcp, are comparable to those of non-calcified cnidarian proteins. The domain architecture of each SOMP is described in more detail below.

### Annotation of transmembrane proteins

Proteins with one or more transmembrane domains were found in the skeletal proteome. Cadherin, neurexin, EGF and laminin G dcps, REJ dcp, zona pellucida dcp, MCO, MAM and LDL receptor dcp, TSP-1 and VWA dcp, and mucin4-like protein were categorized as transmembrane proteins (Fig 1).

The *Acropora* SOMP encoded by transcriptome contig adi_EST_assem_2804 and gene model aug_v2a.19518 had the domain structure typical of classical type-III cadherin [50], including 31 extracellular cadherin domains, 2 laminin G domains, 3 EGF-like domains, a single transmembrane domain, and a classical cadherin cytoplasmic domain (CCD) (Fig 4A, S3 Fig). This domain architecture is widely found in both protostomes and deuterostomes (Fig 3). The intracellular p120-catenin- and β-catenin-binding domains are also retained (S3 Fig). Thus, we annotated this SOMP as cadherin or CDH.

One coral skeletal matrix protein encoded in gene models aug_v2a.24512, had laminin G domains, EGF-like domains, and a transmembrane domain (Fig 4B, S4 Fig). Its overall domain structure was comparable to that of *Drosophila* neurexin IV (S4 Fig), which is the transmembrane component of septate junctions [51–53]. We named this SOMP following the suggestions of Chapman *et al*. [53], who described a putative cnidarian ortholog as “Neurexin.” The domain structure similar to neurexin is expanded in cnidarian species [54] (Fig 3). Two gene models (aug_v2a.06122 and aug_v2a.06123) encoded SOMPs with one or two laminin G domains and one EGF-like domain (S4 Fig). We named them “EGF and laminin G dcp -1 and -2”. In addition, one SOMP, encoded by aug_v2a.15580, was annotated as “Laminin G dcp” since it carried two tandem laminin G domains (S4 Fig). Although this protein does not have a signal peptide or a transmembrane domain, we tentatively classified it as an extracellular matrix-like protein based on its overall domain architecture. Alignment of these four SOMPs showed significant similarity to laminin G and EGF-like domain regions, as well as non-domain regions (S5 Fig), indicating a common origin of these genes.

Gene model aug_v2a.02830 encoded a multi-pass transmembrane protein with a Receptor for Egg Jelly or REJ domain (Fig 4C, S6 Fig), which is found in the polycystic kidney desease-1 (PKD1) gene family [55, 56]. The *A*. *digitifera* REJ domain-containing SOMP has a series of conserved domains, including WSC, PKD repeats, REJ, GPS, and a PLAT domain. PKD1 homologs have been reported in deuterostome animals, including sea urchins [55] and humans [56]. A functional domain search showed that sea anemones also have REJ domain-containing proteins and that the overall domain architecture is very similar to that of the coral SOMP (S6 Fig). It has been reported that the sea urchin SpREJ3 and human Polycystin-1 are cleaved at the G-protein-coupled receptor cleavage site (GPS) [57, 58]. Sequence alignment of the GPS domain showed that the cleavage site is highly conserved among cnidarians and deuterostomes (S6 Fig), suggesting that the coral REJ dcp may also undergo proteolytic processing.

Zona pellucida (ZP) domain proteins are extracellular matrix components found in a diverse array of tissues from many animals [59]. Genes encoding one ZP domain are extensively found in metazoan animals (Fig 3). The primary structure shows that the ZP domain includes consensus cysteine residues that are responsible for intra- and inter-molecular disulfide bridges and polymerization of the proteins (S7 Fig) [60]. In general, ZP proteins undergo proteolytic cleavage and the moiety that carries the ZP domain is released to the extracellular space [59, 61]. Anthozoan ZP proteins retain the conserved cleavage sequence (Arg-X-Lys/Arg-Arg) [62], which is located between the ZP and transmembrane domains (S7 Fig).

Multi-copper oxidase (MCO), with six cupredoxin domains, was also found in the coral skeletal proteome (Fig 4E). In vertebrates, MCOs are involved in iron efflux in various tissues [63], and the ceruloplasmin-type MCO family includes three genes: ceruloplasmin (Cp), hephaestin (Heph), and hephaestin-like (Hephl, also known as zyklopen) [63, 64]. Sequence alignment of mouse six-domain MCOs and the *A*. *digitifera* MCO in the skeleton proteome revealed that the coral MCO retains metal-binding residues in the cupredoxin domains (S8 Fig). Six-domain MCOs had not previously been described from non-chordate animals [65]. We found MCOs with six domains in various non-chordate genomes (Fig 3, S9 Fig). With the aim of clarifying evolutionary relationships among MCOs and of annotating the *Acropora* MCO SOMP precisely, a molecular phylogeny of MCOs was constructed (S10 Fig, S2 Table, S1 Data). The result clearly shows that cnidarian MCOs are clustered together and distinguished from chordate MCOs. Therefore, we annotated this SOMP as multi-copper oxidase or MCO. In addition, two *Acropora* MCOs, present in the skeleton proteome, are closely clustered.

Gene models aug_v2a.09968 and 09969 were tandemly arranged in the scaffold, and cDNA sequence adi_EST_assem_4944 encoded C-terminus of 09968 and N-terminus of 09969, respectively (S11 Fig). Therefore, it is concluded that these two gene models are actually a single gene. The protein product consists of a long repeat of an MAM domain with an LDL receptor repeat, as well as other extracellular matrix domains of fibronectin, EGF, and Trefoil (Fig 4, S12 Fig). The tandem MAM and LDL receptor domains are present in vertebrate transmembrane proteins such as Endotubin (also known as apical early endosomal glycoprotein) [66] and Diet1 [67]. Proteins with MAM, LDLr, and EGF domains were found in cnidarians and bilaterian animals (Fig 3, S12 Fig). Notably, *Nematostella* has two gene models (protein IDs, 198667 and 239043) that are tandemly arranged in scaffold 11, and the domain structures of the two proteins are highly similar to the N- and C- terminal halves of the *A*. *digitifera* SOMP, respectively. Thus, it is likely that *N*. *vectensis* also has homologs of coral MAM and LDLr domain-containing proteins.

The SOMP corresponding to gene model aug_v2a.05945 had three VWA domains, successive TSP-1 domains, and two F5_F8_type_C domains (Fig 4G, S13 Fig). Genome-wide domain searches showed no significant global similarity to available sequences in public databases including those from the *N*. *vectensis*, *Aiptasia*, and *H*. *magnipapillata* genomes, except for *A*. *millepora* SOMP (Fig 3, S13 Fig). Hence, we named this protein “TSP-1 and VWA domain-containing protein.”

The SOMP encoded by gene model aug_v2a.09809 was annotated as a mucin4-like protein, since it contained NIDO, AMOP, VWD, and EGF domains, which are typically present in mucin4 proteins [68] (Fig 4H, S14 Fig). We categorized this protein as a transmembrane protein based on its considerable sequence similarity to the *A*. *millepora* mucin-like SOMP [29], although the *A*. *digitifera* sequence lacked a signal peptide and a transmembrane domain, possibly because both ends of the sequence were truncated. Coral mucin4-like SOMPs have additional TSP1 domains at their N-termini (Fig 4H, S14 Fig). We surveyed public databases and there is no protein sequence with the same domain architecture in any other metazoan (Fig 3).

### Annotation of extracellular matrix-like proteins

Two CUB domain-containing SOMPs, with signal peptides at their N-termini and no other known domains, were found in the skeletal proteome (Fig 4I). Proteins with a single CUB domain are widely distributed in animal genomes (Fig 3). *A*. *digitifera* CUB domain-containing protein 1 (aug_v2a.14283) and 2 (aug_v2a.10941) were closely similar to *A*. *millepora* CUB domain-containing protein and Threonin-rich protein (S15 Fig). These four proteins retain a threonine-rich sequence that is absent in other animal genomes.

*A*. *digitifera* skeletal proteome includes a protein with vitellogenin-like domain architecture, which is composed of vitellogenin N-terminus and VWD domains (Fig 4J). This domain architecture is common to metazoan animals (S16 Fig). However, the coral vitellogenin-like SOMP is unique since the protein carries an additional protein kinase domain (Figs 3 and 4J, S16 Fig).

Two gene models, aug_v2a.22918 and aug_v2a.00002, encode SOMPs similar to *A*. *millepora* USOMP-5 (S17 Fig). These *Acropora* SOMPs share a partial sequence that is significantly conserved in coral egg proteins (EPs) identified from *Euphyllia ancora* [69] and *Galaxea fascicularis* [70]. We therefore annotated these SOMPs as “EP-like” 1 and 2. It is notable that the conserved sequence is also encoded in the *Nematostella* genome (S17 Fig).

### Annotation of galaxins and cysteine-rich proteins

Galaxin is a coral-specific secretory, skeleton matrix protein that has tandem repeat motifs containing di-cysteine sequences (Cys-Cys). Galaxin was originally identified from the coral, *Galaxea fascicularis* [71]. Two galaxins, Adi-galaxin and Adi-galaxin2, have been identified in the *A*. *digitifera* proteome (Fig 1). Galaxins are rich in di-cysteines, which appear 19 times in Adi-galaxin and 13 in Adi-galaxin2 (S3 Table). In addition, another cysteine-rich SOMP was also characterized. This Cys-rich SOMP contains 51 cysteine residues that comprise 10.49% of the mature protein sequence, comparable to the galaxins (12.7% for Adi-galaxin and 11.76% for Adi-galaxin2, respectively). The Cys-rich SOMP had tandem repeat motifs with conserved cysteine residues (S18 Fig), while only 4 di-cysteine blocks were found.

### Annotation of acidic proteins

Genes encoding skeletal acidic proteins or SAPs were predicted in the *A*. *digitifera* genome sequence [34] and it is confirmed that SAP-1 and SAP-2 are components of the skeletal matrix of *A*. *digitifera* (Fig 1). Orthologs of their proteins have been identified in *A*. *millepora*, but not in *S*. *pistillata* [26, 29]. BLAST searches showed that cDNA sequences corresponding to SAP-1 and -2 were not found among ESTs of the corals, *Montastraea faveolata* and *Porites astreoides*; therefore, SAPs appear unique to *Acropora* species [34].

We have found two gene models that are highly similar to *A*. *millepora* secreted acidic Asp-rich protein (SAARP) 2. Alignment of these amino acid sequences deduced from the corresponding cDNAs (S19 Fig) indicated that adi_EST_assem6252 and 6253 may be transcripts of the 5’ and 3’ halves of the SAARP gene. In addition, gene models encoding these two cDNAs (aug_v2a.01441 and aug_v2a_01140, respectively) were located adjacently in the genome (S19 Fig). Therefore, we conclude that these two gene models are really a single gene that encodes Adi-SAARP2. The counterpart of Ami-SAARP1 was also detected in the *A*. *digitifera* proteome. In addition, we have identified another acidic protein encoded in the gene model aug_v2a.06327. We named the protein “Adi-SAARP3,” since its sequence is very similar to that of SAARP1 and 2, both in the acidic and non-acidic regions (S20 and S21 Figs). These *Acropora* SAARPs are also similar to CARP4 and 5 of *S*. *pistillata* [26]. Interestingly, SAARPs and CARPs share significantly similar sequences between acidic domains (S20 and S21 Figs), and the conserved amino acid sequence is also encoded in *Nematostella*, *Hydra*, and, unexpectedly, also in bivalve (*Pinctada* and *Crassostrea*) genomes (S21 Fig), while none of them possess the acidic region (data not shown).

### Annotation of uncharacterized coral-specific SOMPs

The proteome of *A*. *digitifera* contained six unique proteins that showed no significant similarity to any proteins in public databases. Three of them that were comparable to Ami-USOMP-1, were named “Adi-USOMP-1a, b, and c” (S22 Fig). Adi-USOMP-9 displays partial similarity to USOMP-1 proteins (S22 Fig). Two SOMPs named “USOMP-10 and -11” were novel proteins, and there is no corresponding protein in *A*. *millepora* skeletal proteome. Signal peptide searches and secretome detection analysis indicated that all six USOMPs are extracellular proteins (Fig 1, S3 Table).

### Tandem arrangement of SOMP genes in the *A*. *digitifera* genome

In *A*. *digitifera*, two laminin G and EGF dcp genes and two USOMP-1 genes were tandemly arranged in the genome scaffold (S23 Fig). These genes are likely to have emerged due to gene duplication events. High conservation of amino acid sequences (S5 and S22 Figs) and similar gene expression patterns (Fig 2) imply their functional redundancy.

## Discussion

### Identification of coral SOMPs

Scrupulous attention needs to be paid to avoid contamination with proteins not associated with the skeleton, since cellular debris may remain if the coral skeleton is inadequately cleaned [27, 28]. We treated skeleton samples with extended bleaching to remove soft tissue attached to the skeleton surface [29]. We propose that the proteins identified here are veritable SOMPs, for several reasons. First, cell membrane ATPase, which has been detected in calicoblastic cells, but not in the skeleton [23], was not found in the *A*. *digitifera* skeletal proteome. Second, most proteins presented here were also detected in previous, independent experiments in different species, using different cleaning methods. Third, several transmembrane protein families contain various proteins, but only one specific member of the family was found in the present proteomic analysis. For example, the *A*. *digitifera* genome has 51 gene models that encode cadherin domains, but only one cadherin domain-containing protein, of which an ortholog is detected in the *A*. *millepora* and *S*. *pistillata* skeletal proteomes, is evident among *A*. *digitifera* SOMPs. Fourth, in the case of transmembrane proteins, only peptide sequences presumably located in extracellular side were identified in the skeletal proteome (S3 Table). Furthermore, while transmembrane neurexin is found among coral SOMPs, cytoplasmic components of the septate junction, including Gliotactin, Disc large, Scribble, and Coracle [53] were not detected in the coral skeletal proteome. This confirms that the coral skeleton proteome was not contaminated by intracellular components and that neurexin polypeptides are actually associated with the extracellular skeletal matrix. For all of these reasons, the proteins listed in Fig 1 are highly likely to be SOMPs and intimately incorporated into the coral skeleton.

Twenty-six proteins in the *A*. *digitifera* skeleton proteome are shared with *A*. *millepora* (Fig 1, S2 Fig). However, some *A*. *millepora* SOMPs, such as carbonic anhydrase, peptidase, and collagen, were not detected in *A*. *digitifera*. One possible explanation is that the gene gain/loss or change of protein function that occurred after the divergence of *A*. *digitifera* and *A*. *millepora*, resulted in different skeletal matrix proteomes in these two species. However, such a significant change is unlikely to have occurred after the separation of these closely related species. Therefore, we believe that the difference between the two *Acropora* proteomes is likely due to technical variability. Two *A*. *digitifera* SOMPs, vitellogenin and Adi-USOMP-11 were not identified in *A*. *millepora* SOMP (S2 Fig) and their presence in *A*. *digitifera* SOMP was supported by a relatively low number of peptides (18 and 9, respectively, S3 Table). It remains possible that these extracellular proteins are randomly incorporated into the skeleton. It is also probable that enzymes like carbonic anhydrase and peptidase, which were exclusively identified from *A*. *millepora* SOMP, function in soluble form and not in the solid skeleton. We speculate such enzymes are incidentally included in the coral skeleton, and that they are weakly associated with calcium carbonate crystals. Thus, these proteins are easily removed by strict sample cleaning. Further experiments with different sample cleaning and extraction conditions and studies of other coral species should confirm lineage-specific and conserved coral skeleton proteins.

The *in silico* prediction of protein localization suggests that all *A*. *digitifera* SOMPs are exposed in the subcalicoblastic space, where calcification occurs. Two gene models (aug_v2a.09809 and adi_v2a.15580) and their corresponding mRNA sequences predict no signal peptides at the N-termini and show low NN-scores, presumably due to truncated sequences. These proteins (laminin G dcp and mucin4-like protein) may also be located in the extracellular space, according to their functional domain architecture (S4 and S14 Figs).

### Gene expression profile of SOMPs during development

Gene expression analysis of SOMPs throughout coral development provides new insights into the biological role of the proteins. Many transmembrane SOMP and laminin G dcp genes were expressed throughout developmental stages (Fig 2), indicating that these proteins have general cellular functions related to development, in addition to skeleton formation. Sixteen genes are expressed in both polyp and adult stages, suggesting that they are responsible for larval and adult skeleton formation. This group includes MAM and LDLr dcp, TSP1 and VWA dcp, extracellular matrix-like proteins, cysteine-rich proteins, acidic proteins except SAP-1, and uncharacterized protein -1 and -9. Other genes, encoding mucin4-like, Adi-SAP1, USOMP10, and 11, are expressed only in adults. Therefore, these proteins have a specialized role in adult calcification.

The differential expression of SOMP genes in polyps and adults (Fig 2A) indicates that the molecular basis of initial skeleton formation is not identical to that of the adult skeleton. This is concordant with SEM observations, showing that settled polyps generate disk-like structures on the substrate during the initial step in skeleton formation (Fig 2B), and subsequently, they begin to construct a network structure similar to adult skeleton (Fig 2C). Moreover, smaller and less ordered crystals in the primary skeleton [31, 33] may also be related to the lack of adult-specific SOMPs, and it is also possible that there are polyp-specific SOMPs that were not examined here. Further investigation including the larval skeleton proteome will be necessary in order to complete the inventory of coral skeleton SOMPs.

Transcripts of SOMP genes encoding CUB domain-containing protein, Galaxin, SAARP1, USOMP-1b, and -1c, were detected weakly in the egg and prawnchip stages (Fig 2). This may occur because maternal mRNAs are provided to the egg and are retained in early developmental stages in *Acropora* [72], but it is not certain that these SOMP gene transcripts are translated and functional in early development.

### Putative function of coral SOMPs

In total, twelve transmembrane proteins were identified in the *A*. *digitifera* skeletal proteome (Fig 1, S3 Table). Interestingly, many of these, including cadherin, neurexin, REJ dcp, ZP dcp, MCO, MAM and LDLr dcp, were remarkably similar to transmembrane proteins of non-calcifying animals, such as sea anemones (Fig 4).

Cadherin is a cell-cell adhesion protein shared by all metazoans [50, 73, 74]. The overall domain structure of coral cadherin SOMP is highly similar to the counterpart found in other metazoans (Figs 3 and 4A, S3 Fig), and the conserved p120-catenin- and β-catenin-binding domains in the CCD suggest that the intracellular region is functional, interacting with the actin cytoskeleton, mediated by catenins [75] (S3 Fig). The gene expression profile showed that the *A*. *digitifera* cadherin gene is expressed throughout development (Fig 2). Therefore, the cadherin SOMP may have inherent functions of cell-cell adhesion and may function in a variety of developmental processes [73, 76, 77], later acquiring the additional function of skeleton formation. One possible role of the cadherin SOMP in skeleton formation is the link between cells and organic matrix in the skeleton, inferred from its primary role in cell adhesion. Detachment of the polyp from the skeleton was observed when the animal was subjected to low calcium seawater [78], possibly because cadherin, the adhesion function of which is calcium-dependent, mediates attachment of the animal. Besides, it is known that in some biological processes, the classical cadherin ectodomain can be released into the extracellular space by proteolytic cleavage [79, 80]. Coral cadherin SOMP may also be shed, and the extracellular peptides with cadherin domains may help to build up the organic matrix. It is possible that intact, cell surface cadherin molecules combine with the cleaved moiety that constitutes the organic matrix, forming a close association between ectodermal cells and skeleton. Similarly, REJ dcp, and ZP dcp carry a proteolytic cleavage site upstream of the transmembrane domain (S3 and S4 Figs). N-terminal polypeptides of these proteins may be released into the extracellular subcalicoblastic space and where they constitute the organic matrix. Cell membrane proteins exposed to the extracellular space may connect to the organic matrix, and participate in settlement on the substrate, as well as maintaining the attachment to the skeleton.

On the other hand, the remaining transmembrane SOMPs do not contain such specific cleavage domains. Ramos-Silva *et al*. [29] showed that extracellular proteases were detected in the *A*. *millepora* proteome and that those enzymes were responsible for digestion of transmembrane proteins. The same process may also explain the presence of peptides of transmembrane proteins in the *A*. *digitifera* skeletal proteome. In addition, it is also possible that transmembrane proteins are anchored to the cell membrane and interact with calcium carbonate crystals *in vivo*. For example, Adi-SAARP2 and 3 carry one transmembrane domain at their C-terminal ends while the aspartic acid-rich domains are positioned at the N-terminal ends. It is conceivable that the Asp-rich domains bind to the calcium carbonate surface and make close attachments between calicoblastic ectoderm and the crystal surface. The mammalian multi-copper oxidase, ceruloplasmin, has an affinity for calcium ions [81], giving rise to the idea that Adi-MCO can mediate the interaction between cell membranes and calcium carbonate crystals. The idea of cell-skeleton interaction mediated by transmembrane proteins is congruent with previous direct observations that calicoblastic ectoderm is intimately associated with the mineral phase [19, 22]. Desmocytes, which are the anchoring cells in calicoblastic ectoderm [82], maintain the attachment to the skeleton by extending fibrils into the organic matrix [83]. Desmocytes associated with the organic matrix are responsible for attachment to the substrate of the octocorallian soft coral, *Dendronephthya hemprichi* [84], suggesting that the primal role of the anthozoan organic matrix produced by aboral ectoderm is for settlement of the polyp, and that it was subsequently utilized for skeletal organization in the scleractinian lineage.

In *A*. *digitifera* SOMPs, there are many extracellular matrix protein domains, such as cadherin, EGF, laminin_G, VWA, MAM, LDLr, fibronectin, TSP-1, VWA, vitellogenin, and CUB. Proteins and other organic components of the extracellular matrix, such as polysaccharides, interact with each other via these domains and constitute the organic framework. Galaxins and Cys-rich SOMPs may also be involved in the organic matrix framework [71].

Acidic proteins are key components in biomineralization, since aspartic acid (Asp) and glutamic acid (Glu) are negatively charged at neutral pH and are able to interact with calcium ions [85, 86]. Many vertebrates and invertebrates employ acidic proteins for biomineral formation [87–91]. Previous studies have shown that aspartic acid and glutamic acid are major components of the amino acid fraction of the coral skeleton [11, 92, 93]. Our proteomic analysis detected many polypeptides derived from acidic proteins. For example, the spectral counts of Adi-SAP2 and Adi-SAARP1 are 4,703 and 3,310, respectively, and these are the most abundant *A*. *digitifera* SOMPs (S3 Table). This means the aspartic acid-rich proteins are abundant in the *A*. *digitifera* skeletal proteome. Therefore, SAPs and SAARPs are candidate proteins for major roles in CaCO_3_ crystallization including nucleation, growth, and inhibition. Acidic transmembrane proteins (SAARP-2 and -3) may also mediate the close association between cells and skeletal crystals. In addition to acidic regions, SAARPs contain a conserved sequence that is present in *Nematostella*, *Hydra*, and two bivalve genomes (S21 Fig). Six of these non-coral proteins are secretory (data not shown), implying that the conserved sequence may have a similar function in the extracellular space.

### Evolutionary origin of coral SOMPs

**Fig 5:**
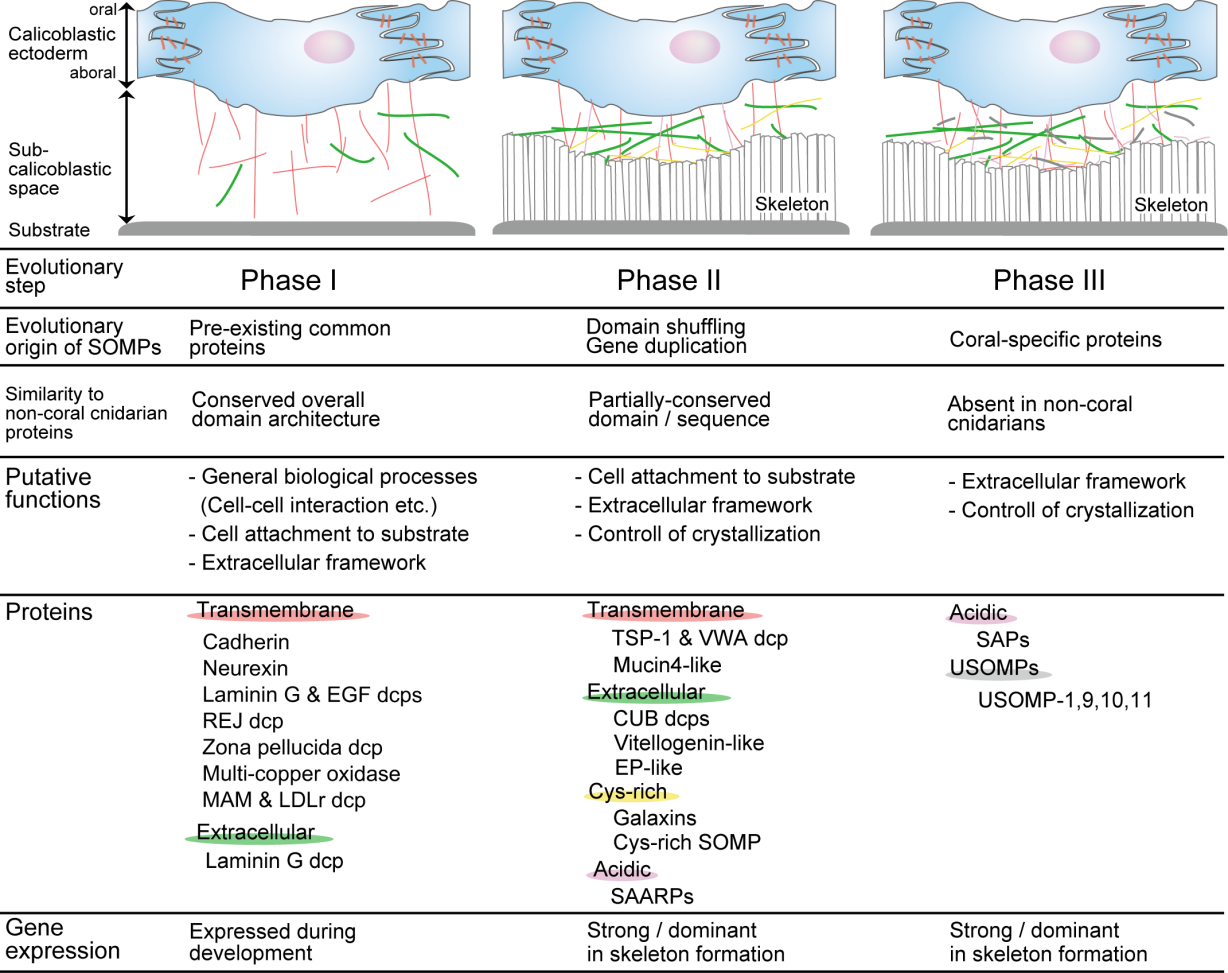
Evolutionary and stepwise acquisition processes for coral skeleton formation. In Phase I, pre-existing proteins that functioned in cellular activity and developmental processes in non-skeletal cnidarians, may have acquired additional functions, presumably for attachment to the substrate and to constitute the initial extracellular organic matrix. In Phase II, novel proteins emerged after gene duplication, domain shuffling, and rapid molecular evolution, and became specifically involved in constructing the organic matrix framework. Finally, unique proteins emerged in the coral lineage (Phase III). They may have interacted with other organic matrix proteins to play important roles in crystal formation.

We classified *A*. *digitifera* SOMPs into three evolutionary phases, according to the similarity of protein domain structure to non-coral cnidarian (*Nematostella* and *Aiptasia*) proteins (Fig 5, Phases I, II, and III). Many transmembrane SOMPs have apparent homologs in cnidarian and bilaterian genomes (Fig 4, S3, S4, S6, S7, S9, S10 and S12 Figs), indicating that these proteins were present in uncalcified cnidarian ancestors (Fig 5, Phase I).

TSP-1 and VWA dcp, mucin4-like, and vitellogenin-like protein contain unique domain architecture that is absent in genomes of non-skeletal cnidarians and other metazoans (Figs 3 and 4, S13, S14 and S16 Figs). We defined these as Phase II proteins (Fig 5). The TSP1, VWA, and F5/F8 type C domains are widely distributed among phylogenetically disparate organisms; however, these three domains do not normally occur in the same protein (Fig 3, S13 Fig). Coral mucin4-like SOMPs have repeated TSP1 domains at their N-termini, in addition to typical mucin4 domain structure, and the overall domain structure is unique to corals (Figs 3 and 4H, S14 Fig). Likewise, *Acropora* vitellogenin-like SOMP is composed of similar domain architecture to vitellogenin, and specifically has a protein kinase domain (Fig 4J, S16 Fig). It is hypothesized that these three SOMPs appeared in the coral lineage by domain shuffling after divergence of corals from other anthozoans.

Phase II proteins also include CUB dcps, EP-like proteins, galaxins, Cys-rich, and SAARPs. The CUB dcps and the EP-like proteins retain partial sequences that are comparable to non-coral cnidarian proteins (Fig 4I, S17 Fig). The restricted sequence similarity indicates these proteins have been modified to engage coral-specific, skeletal matrix formation process. Proteins similar to galaxin, with repeated motifs containing di-cysteine, are found in non-skeletal cnidarians such as *Hydra* and *Nematostella* [29]. Detectable sequence similarity suggests that coral cysteine-rich SOMPs and non-coral cnidarian proteins may share an evolutionary origin. Therefore, we put galaxin and Cys-rich SOMP in the Phase II category (Fig 5). Galaxin-like genes have been reported in *Acropora* species [34, 94], while protein products of these genes are not present in the skeleton proteome. Cysteine-rich proteins may originally have been responsible for biological processes unrelated to biomineralization, then utilized for skeletal formation after rapid evolution of this gene family in the coral lineage. SAARPs include a conserved region that is quite similar to those of non-coral cnidarian and bivalves (S21 Fig). The conserved sequence may be derived from a metazoan ancestor, and may have an unknown function. The acidic amino acid regions that are thought to be important for crystallization processes were specifically acquired by SAARPs. In addition, lineage-specific expansion of SOMP genes seems to have occurred among CUB dcp, EP-like, galaxin and SAARP genes. Gene duplication events are also evident in matrix proteins of molluscs [95] and vertebrates [96], reflecting the rapid molecular evolution of biomineralization-related genes.

Finally, SAPs and uncharacterized SOMPs are classified as Phase III (Fig 5), since they have unique sequences such as an acidic domain that is not present in non-coral cnidarians. The evolutionary origin of these proteins is unclear, but it is possible they emerged by rapid molecular evolution, which expunges detectable sequence similarity to non-coral animal proteins.

### Stepwise evolution of coral biomineralization

In biomineralization, organic matrix provided by cells plays a pivotal role in controlling hard tissue formation. The present and previous studies confirm that more than 30 proteins are present in the coral skeletal proteome.

Based on thorough gene annotation of coral SOMPs, stepwise evolution of coral biomineralization is hypothesized. We found that many genes are closely related to those of the sea anemones, *N*. *vectensis* and *Aiptasia*, indicating that coral genes were derived from genes of a non-calcified anthozoan ancestor (Fig 5, Phase I). The majority of transmembrane proteins are involved in this phase. The coral skeletal organic matrix is also composed of newly innovated proteins that manifest reduced similarity to other cnidarian proteins (Fig 5, phase II). Partial sequence conservation in domains indicates that these proteins can be traced to those of non-calcified cnidarians or common metazoan ancestors. These proteins are thought to have emerged by domain shuffling or rapid molecular evolution, and to have been recruited to function in the skeletal organic matrix. Finally, skeletal acidic proteins and uncharacterized proteins in the organic matrix are thought to have emerged after coral divergence from non-skeletal anthozoans (Fig 5, Phase III). To examine the hypothesis, experimental functional analysis such as gene knockdown, identification of SOMPs from different coral species, and comparative genomics using more cnidarian species are needed.

## Conclusions

The skeleton of the coral, *A*. *digitifera*, is composed of many transmembrane and extracellular matrix proteins inherited from uncalcified ancestors, indicating that corals first used pre-existing proteins for skeleton formation. Second, settlement and attachment to the substrate mediated by transmembrane proteins and extracellular matrix may be the initial phase of skeleton formation. Third, thereafter, coral-specific molecules, such as acidic proteins, control the calcification process. The annotation of biomineralization-related genes presented here will be helpful for further research, such as functional analysis of each gene. Whole genome information will also provide us with essential information for investigating coral calcification, including cellular physiology and response to environmental change.

## Supporting Information

S1 Data Amino acid sequence alignment of six-domain multi-copper oxidases used for molecular phylogeny in S10 Fig. (TXT)

S1 Fig SDS-PAGE analysis of the ASM and AIM fractions of the skeleton. Acid soluble matrix (ASM) and acid insoluble matrix (AIM) fractions of the skeleton stained by silver staining. Arrowheads indicate visible bands.
(PDF)

S2 Fig Venn diagram comparing SOMPs identified in three coral species. The majority of transmembrane proteins are found in all three proteomes. The difference between proteomes of two closely related species, *Acropora digitifera* and *A*. *millepora*, is presumably due to technical issues.
(PDF)

S3 Fig Structure of the cadherin SOMP of *Acropora digitifera* and cadherins of other representative animals. (a) Schematic domain structure of cadherin proteins. The *A*. *digitifera* cadherin SOMP has a domain structure typical of non-chordate metazoans. Lengths of amino acid sequences are shown at the right. (b) Alignment of amino acid sequences in the cytoplasmic domain of cadherins. *A*. *digitifera* cadherin SOMP retains conserved motifs of p120-catenin and β-catenin-binding sequences. Conserved amino acid positions are highlighted with blue. Transcript ID, gene model ID, and NCBI accession ID of the proteins are as follows: *A*. *digitifera* cadherin (adi_EST_assem_2804), *N*. *vectensis* CDH1 (XP_001631293.1), *D*. *melanogaster* cadherin-N (NP_001027277.1), *T*. *adhaerens* TaCDH (Triad1|55710), *A*. *millepora* cadherin (JT011093), and *M*. *musculus* Cad-1 (NP_033994.1).
(PDF)

S4 Fig Neurexin protein architectures of selected animals and related proteins of *A*. *digitifera*. (a) Domain architecture of neurexin is conserved among cnidarian species. (b) EGF-like and laminin G dcps. (c) Laminin G dcp. Lengths of amino acid sequences are shown at the right.
(PDF)

S5 Fig Alignment of coral neurexin and related proteins. *A*. *digitifera* neurexin (aug_v2a.24512), EGF-like and laminin G dcps (aug_v2a.06122, aug_v2a.06123), laminin G dcp (aug_v2a.15580), and *A*. *millepora* EGF and laminin G dcp (JR980881.1) are aligned. Conserved residues are highlighted with blue and the transmembrane domain is underlined with purple.
(PDF)

S6 Fig Domain structure of REJ domain-containing proteins. (a) The conserved domain structure of PKD1 proteins in a coral and representative animals. The lengths of amino acid sequences are shown at the right. (b) Sequence alignment of GPS domains. The arrow indicates the putative cleavage site of the domain. Gene model IDs or NCBI accession IDs of the proteins are as follows: *A*. *digitifera* (aug_v2a.02830.t1, adi_EST_assem_6849), *N*. *vectensis* (Nmeve1|196807), *S*. *purpuratus* SpREJ3 (AAL26499.1), and *H*. *sapiens* PC1 (NP_001009944.2).
(PDF)

S7 Fig Sequence alignment of cnidarian zona pellucida (ZP) domain-containing proteins. Conserved cysteine residues in the ZP domain are marked by asterisks. The ZP domain underlined with red is immediately followed by a putative proteolytic cleavage site (Arg-X-Lys/Arg-Arg) underlined with green. The transmembrane domain is underlined with purple. Conserved amino acid positions are highlighted with blue. The transcriptome IDs, gene model IDs, or NCBI accession IDs of the proteins are as follows: *A*. *digitifera* ZP dcp N-terminus (adi_EST_assem_1474), C-terminus (adi_EST_2269), *A*. *millepora* (AET09743.1), *N*. *vectensis* (Nemve1|204835), and *Aiptasia pallida* (JV132371.1).
(PDF)

S8 Fig Alignment of coral multi-copper oxidase (MCO) and mouse MCO. Cu-binding histidine residues are highlighted in cyan for type I, pink for type II, and yellow for type III. Metal-binding residues are highlighted in green. Transmembrane domains are shaded in purple. Cupredoxin domains inferred from mouse Hephaestin are boxed in red for the 1st, 3rd, and 5th, and blue for the 2nd, 4th, and 6th, respectively. Transcriptome IDs or NCBI accession IDs of the proteins are as follows: *A*. *digitifera* MCO N-terminus (adi_EST_assem_20166), C-terminus (adi_EST_assem_13507), *M*. *musculus* Cp (NP_001263177.1), Heph (NP_001153099.1), and Hephl (NP_001158269.1).
(PDF)

S9 Fig Six domain multi-copper oxidase architectures of selected animals. *Acropora* multi-copper oxidase SOMP is similar to that of *Aiptasia*. In addition, the domain architecture with six cupredoxin domains is conserved among bilaterian animals. Lengths of amino acid sequences are shown at the right.
(PDF)

S10 Fig Maximum likelihood (ML) molecular phylogenetic tree of six-domain multi-copper oxidases (MCOs). Sequences were aligned with MUSCLE, and poorly aligned positions were removed with Gblocks. Then, the resulting dataset (S1 Data) was used for phylogenetic analysis. An ML tree was constructed using the LG + GAMMA model. Cnidarian MCOs are clustered (orange) separately from vertebrate Hephaestin, Hephaestin-like, Ceruloplasmin (green), and Coagulation factors (Cyan). Coral MCO skeletal matrix proteins are highlighted in red. Bootstrap values for the ML analysis are shown at each node. Nodes supported by neighbor-joining (NJ) analysis with high bootstrap support (BS≥70%) are gray-dotted, and nodes supported by NJ (BS≥70%) as well as by Bayesian inference with high posterior probability (≥95%) are black-dotted. The scale bar represents the number of expected substitutions per site in the aligned regions. Abbreviations for protein sequences are presented in S2 Table.
(PDF)

S11 Fig Gene structure of MAM and LDLr domain- containing proteins. (a) Tandem arrangement of two gene models (aug_v2a.09968 and aug_v2a.09969) encoding MAM domains in the single scaffold of the *A*. *digitifera* genome assembly. (b) Sequence alignment of two gene models (aug_v2a.09968 and aug_v2a.09969), termini of which are connected by the sequence deduced from cDNA (adi_EST_assem_4944), indicating that these two gene models may be one gene that encodes a MAM and LDLr domain-containing protein.
(PDF)

S12 Fig Domain architecture of MAM and LDLr dcps of metazoan animals. Lengths of amino acid sequences are shown at the right.
(PDF)

S13 Fig Coral TSP-1 and VWA domain-containing SOMPs. This domain architecture is only found in *Acropora* species. Lengths of amino acid sequences are shown at the right.
(PDF)

S14 Fig Domain structure of MUC4 and mucin4-like proteins of representative animals. Coral mucin4-like SOMPs contain NIDO, AMOP, VWD, and EGF domains, which are typically present in mucin4 of other animals. In addition, coral mucin4-like proteins have TSP1 domains. Lengths of amino acid sequences are shown at the right.
(PDF)

S15 Fig Alignment of CUB domain containing SOMPs of *Acropora* species. *A*. *digitifera* CUB dcps and *A*. *millepora* CUB dcp have a single CUB domain underlined with red, which is inferred from Adi_CUB dcp sequences with the InterProScan. The threonine-rich SOMP of *A*. *millepora* has a conserved sequence in the N-terminus region, while it lacks a CUB domain. Conserved amino acid positions are highlighted with blue. Transcriptome IDs or NCBI accession IDs of the proteins are as follows: *A*. *digitifera* CUB dcp-1 N-terminus (adi_EST_assem_9510), CUB dcp-1 C-terminus (adi_EST_assem_5604), CUB dcp-2 N-terminus (adi_EST_assem_30005), CUB dcp-2 C-terminus (adi_EST_assem_21039), *A*. *millepora* Threonine-rich protein (JT013896.1), and CUB dcp (JR989025).
(PDF)

S16 Fig Vitellogenin domain architectures of selected animals. The *Acropora* vitellogenin-like SOMP has a protein kinase domain, which is absent from the other metazoan vitellogenins. Lengths of amino acid sequences are shown at the right.
(PDF)

S17 Fig Sequence alignment of coral egg proteins, EP-like proteins, and a similar protein of *Nematostella*. Local sequence similarity is found in middle of the proteins. Conserved positions are shaded in blue. Gene model IDs and NCBI accession IDs of the proteins are as follows: *A*. *digitifera* EP-like-1 (aug_v2a.22918.t1), EP-like-2 (aug_v2a.00002.t1), *A*. *millepora* USOMP-5 (B8VIU6.1), *Euphyllia ancora* egg protein (AGO04749.1), *Galaxea fascicularis* egg protein (BAE94663.1), and *N*. *vectensis* (Nemve1|248548).
(PDF)

S18 Fig Alignment of repetitive sequences in the Cys-rich SOMP. Conserved cysteine residues are colored in yellow. Amino acid positions are shown at the left.
(PDF)

S19 Fig Amino acid sequences and gene structure of SAARPs. (a) Alignment of amino acid sequences of *A*. *digitifera* SAARPs. Gene model IDs are as follows: Adi-SAARP1(adi_EST_assem_12928), Adi-SAARP2_N (adi_EST_assem_6252), Adi-SAARP2_C (aug_v2a.01440), and Adi-SAARP3 (adi_EST_assem_995). (b) Genomic positions of gene models aug_v2a.01441 and aug_v2a_01140, consecutively located in the same scaffold, presumably encode one protein Adi-SAARP2.
(PDF)

S20 Fig Conserved domain structures of coral SAARPs and CARPs. All these proteins share non-acidic, conserved sequences between acidic domains (red lines). Lengths of amino acid sequences are shown at the right.
(PDF)

S21 Fig Alignment of non-acidic, conserved sequences of coral SAARPs, CARPs, and other metazoan proteins. Sequence positions are indicated in parentheses. Conserved amino acid positions are highlighted with blue.
(PDF)

S22 Fig Sequence alignment of the Adi-USOMP-1 family and USOMP-9. Conserved positions are shaded in blue. Gene model IDs or transcriptome IDs of the proteins are as follows: *A*. *digitifera* USOMP-1a (aug_v2a.21723.t1), USOMP-1b (aug_v2a.02662.t1), USOMP-1c (aug_v2a.02663.t1), USOMP-9 (aug_v2a.20893), and *A*. *millepora* USOMP-1 (JT021412.1).
(PDF)

S23 Fig Tandem arrangement of SOMP genes in the *A*. *digitifera* genome scaffold. (PDF)

S1 Table List of species used for functional domain analysis. (XLSX)

S2 Table List of multi-copper oxidase protein sequences used for molecular phylogenetic analysis in S10 Fig. (XLSX)

S3 Table Detailed list of skeletal organic matrix proteins in *A*. *digitifera*. (XLSX)

## Abbreviations

AIM: acid insoluble matrix
ASM: acid soluble matrix
CCD: conserved cytoplasmic domain
CUB: complement C1r/C1s, Uegf, Bmp1
dcp: domain-containing protein
DUF: domain of unknown function
LDLr: low-density lipoprotein receptor
MAM: Meprin, A5, μ
MCO: multi-copper oxidase
ML: maximum likelihood
MTP: microsomal triglyceride transfer protein
NJ: neighbor-joining
PKD: polycystic kidney disease
SAARP: secreted acidic Asp-rich protein
SAP: skeletal acidic protein
SOMP: skeletal organic matrix protein
TSP-1: thrombospondin type 1
USOMP: uncharacterized skeletal organic matrix protein
VWA: von Willebrand factor type A
ZP: zona pellucida

## References

[1] Kleypas JA, Yates KK. Coral Reefs and Ocean Acidification. Oceanography. 2009;22:108–17.

[2] Knowlton N, Brainard RE, Fisher R, Moews M, Plaisance L, Caley MJ. Coral reef biodiversity In: McIntyre AD, editor. Life in the World's Oceans: Wiley-Blackwell; 2010 p. 65–78.

[3] Hoegh-Guldberg O, Mumby PJ, Hooten AJ, Steneck RS, Greenfield P, Gomez E, et al Coral reefs under rapid climate change and ocean acidification. Science. 2007;318(5857):1737–42. 18079392 10.1126/science.1152509

[4] Stanley SM. Influence of seawater chemistry on biomineralization throughout phanerozoic time: Paleontological and experimental evidence. Palaeogeogr Palaeoclimatol Palaeoecol. 2006;232(2–4):214–36.

[5] Stanton R Jr, Flügel E. Palecology of upper triassic reefs in the Northern Calcareous Alps: Reef communities. Facies. 1987;16(1):157–85.

[6] Stanley GD, Fautin DG. Paleontology and evolution—The origins of modern corals. Science. 2001;291(5510):1913–4. 11245198 10.1126/science.1056632

[7] Stanley GD. The evolution of modern corals and their early history. Earth-Science Reviews. 2003;60(3–4):195–225.

[8] Medina M, Collins AG, Takaoka TL, Kuehl JV, Boore JL. Naked corals: skeleton loss in Scleractinia. Proc Natl Acad Sci U S A. 2006;103(24):9096–100. 16754865 10.1073/pnas.0602444103 PMC1482572

[9] Lowenstam HA, Weiner S. On Biomineralization New York: Oxford University Press 1989.

[10] Mann S. Molecular recognition in biomineralization. Nature. 1988;332(6160):119–24.

[11] Constantz B, Weiner S. Acidic macromolecules associated with the mineral phase of scleractinian coral skeletons. J Exp Zool. 1988;248(3):253–8.

[12] Mitterer RM. Amino-acid composition and metal-binding capability of skeletal protein of corals. Bull Mar Sci. 1978;28(1):173–80.

[13] Isa Y, Okazaki M. Some observations on the Ca2+ binding phospholipid from scleractinian coral skeletons. Comp Biochem Physiol B Biochem Mol Biol. 1987;87(3):507–12.

[14] Cuif JP, Dauphin Y, Doucet J, Salome M, Susini J. XANES mapping of organic sulfate in three scleractinian coral skeletons. Geochim Cosmochim Acta. 2003;67(1):75–83.

[15] Goldberg WM. Acid polysaccharides in the skeletal matrix and calicoblastic epithelium of the stony coral *Mycetophyllia reesi*. Tissue Cell. 2001;33(4):376–87. 11521954 10.1054/tice.2001.0191

[16] Addadi L, Joester D, Nudelman F, Weiner S. Mollusk shell formation: A source of new concepts for understanding biomineralization processes. Chem Eur J. 2006;12(4):981–7. 10.1002/chem.200500980 16315200

[17] Belcher AM, Wu XH, Christensen RJ, Hansma PK, Stucky GD, Morse DE. Control of crystal phase switching and orientation by soluble mollusc-shell proteins. Nature. 1996;381(6577):56–8.

[18] Mann S. Biomineralization London: Oxford University Press 2001.

[19] Tambutté E, Allemand D, Zoccola D, Meibom A, Lotto S, Caminiti N, et al Observations of the tissue-skeleton interface in the scleractinian coral *Stylophora pistillata*. Coral Reefs. 2007;26(3):517–29.

[20] Isa Y. An electron-microscope study on the mineralization of the skeleton of the staghorn coral *Acropora hebes*. Mar Biol. 1986;93(1):91–101.

[21] Clode PL, Marshall AT. Low temperature FESEM of the calcifying interface of a scleractinian coral. Tissue Cell. 2002;34(3):187–98. 12182812 10.1016/s0040-8166(02)00031-9

[22] Clode PL, Marshall AT. Calcium associated with a fibrillar organic matrix in the scleractinian coral *Galaxea fascicularis*. Protoplasma. 2003;220(3–4):153–61. 12664279 10.1007/s00709-002-0046-3

[23] Puverel S, Tambutté E, Zoccola D, Domart-Coulon I, Bouchot A, Lotto S, et al Antibodies against the organic matrix in scleractinians: a new tool to study coral biomineralization. Coral Reefs. 2005;24(1):149–56.

[24] Mass T, Drake JL, Peters EC, Jiang W, Falkowski PG. Immunolocalization of skeletal matrix proteins in tissue and mineral of the coral *Stylophora pistillata*. Proc Natl Acad Sci U S A. 2014;111(35):12728–33. 10.1073/pnas.1408621111 25139990 PMC4156710

[25] Goffredo S, Vergni P, Reggi M, Caroselli E, Sparla F, Levy O, et al The skeletal organic matrix from Mediterranean coral *Balanophyllia europaea* influences calcium carbonate precipitation. PLoS One. 2011;6(7):e22338 10.1371/journal.pone.0022338 21799830 PMC3142144

[26] Drake JL, Mass T, Haramaty L, Zelzion E, Bhattacharya D, Falkowski PG. Proteomic analysis of skeletal organic matrix from the stony coral *Stylophora pistillata*. Proc Natl Acad Sci U S A. 2013;110(10):3788–93. 10.1073/pnas.1301419110 23431140 PMC3593878

[27] Drake JL, Mass T, Haramaty L, Zelzion E, Bhattacharya D, Falkowski PG. Reply to Ramos-Silva et al: Regarding coral skeletal proteome. Proc Natl Acad Sci U S A. 2013;110(24):E2147–E8. 23905183 10.1073/pnas.1304591110 PMC3683761

[28] Ramos-Silva P, Marin F, Kaandorp J, Marie B. Biomineralization toolkit: The importance of sample cleaning prior to the characterization of biomineral proteomes. Proc Natl Acad Sci U S A. 2013;110(24):E2144–E6. 10.1073/pnas.1303657110 23645633 PMC3683792

[29] Ramos-Silva P, Kaandorp J, Huisman L, Marie B, Zanella-Cleon I, Guichard N, et al The skeletal proteome of the coral *Acropora millepora*: the evolution of calcification by co-option and domain shuffling. Mol Biol Evol. 2013;30(9):2099–112. 10.1093/molbev/mst109 23765379 PMC3748352

[30] Jell JS. Skeletogenesis of newly settled planulae of the hermatypic coral *Porites lutea*. Acta Palaeontologica Polonica. 1980;25(3–4):311–20.

[31] Vandermeulen JH, Watabe N. Studies on reef corals. I. Skeleton formation by newly settled planula larva of *Pocillopora damicornis*. Mar Biol. 1973;23(1):47–57.

[32] Le Tissier MDAA Patterns of formation and the ultrastructure of the larval skeleton of *Pocillopora damicornis*. Mar Biol. 1988;98(4):493–501.

[33] Gilis M, Meibom A, Domart-Coulon I, Grauby O, Stolarski J, Baronnet A. Biomineralization in newly settled recruits of the scleractinian coral *Pocillopora damicornis*. J Morphol. 2014;275(12):1349–65. 10.1002/jmor.20307 24966116

[34] Shinzato C, Shoguchi E, Kawashima T, Hamada M, Hisata K, Tanaka M, et al Using the *Acropora digitifera* genome to understand coral responses to environmental change. Nature. 2011;476(7360):320–U82. 10.1038/nature10249 21785439

[35] Trapnell C, Pachter L, Salzberg SL. TopHat: discovering splice junctions with RNA-Seq. Bioinformatics. 2009;25(9):1105–11. 10.1093/bioinformatics/btp120 19289445 PMC2672628

[36] Trapnell C, Williams BA, Pertea G, Mortazavi A, Kwan G, van Baren MJ, et al Transcript assembly and quantification by RNA-Seq reveals unannotated transcripts and isoform switching during cell differentiation. Nat Biotech. 2010;28(5):511–5. 10.1038/nbt.1621 PMC3146043 20436464

[37] Yamada L, Saito T, Taniguchi H, Sawada H, Harada Y. Comprehensive egg coat proteome of the ascidian *Ciona intestinalis* reveals gamete recognition molecules involved in self-sterility. J Biol Chem. 2009;284(14):9402–10. 10.1074/jbc.M809672200 19193647 PMC2666592

[38] Araki Y, Shimizu HD, Saeki K, Okamoto M, Yamada L, Ishida K, et al A surface glycoprotein indispensable for gamete fusion in the social amoeba *Dictyostelium discoideum*. Eukaryotic Cell. 2012;11(5):638–44. 10.1128/EC.00028-12 22389384 PMC3346428

[39] Jones P, Binns D, Chang HY, Fraser M, Li W, McAnulla C, et al InterProScan 5: genome-scale protein function classification. Bioinformatics. 2014;30(9):1236–40. 10.1093/bioinformatics/btu031 24451626 PMC3998142

[40] Petersen TN, Brunak S, von Heijne G, Nielsen H. SignalP 4.0: discriminating signal peptides from transmembrane regions. Nat Methods. 2011;8(10):785–6. 10.1038/nmeth.1701 21959131

[41] Bendtsen JD, Jensen LJ, Blom N, Von Heijne G, Brunak S. Feature-based prediction of non-classical and leaderless protein secretion. Protein Eng Des Sel. 2004;17(4):349–56. 15115854 10.1093/protein/gzh037

[42] Krogh A, Larsson B, von Heijne G, Sonnhammer EL. Predicting transmembrane protein topology with a hidden Markov model: application to complete genomes. J Mol Biol. 2001;305(3):567–80. 11152613 10.1006/jmbi.2000.4315

[43] Blom N, Gammeltoft S, Brunak S. Sequence and structure-based prediction of eukaryotic protein phosphorylation sites. J Mol Biol. 1999;294(5):1351–62. 10600390 10.1006/jmbi.1999.3310

[44] Edgar RC. MUSCLE: multiple sequence alignment with high accuracy and high throughput. Nucleic Acids Res. 2004;32(5):1792–7. 15034147 10.1093/nar/gkh340 PMC390337

[45] Waterhouse AM, Procter JB, Martin DM, Clamp M, Barton GJ. Jalview Version 2—a multiple sequence alignment editor and analysis workbench. Bioinformatics. 2009;25(9):1189–91. 10.1093/bioinformatics/btp033 19151095 PMC2672624

[46] Castresana J. Selection of conserved blocks from multiple alignments for their use in phylogenetic analysis. Mol Biol Evol. 2000;17(4):540–52. 10742046 10.1093/oxfordjournals.molbev.a026334

[47] Stamatakis A. RAxML version 8: a tool for phylogenetic analysis and post-analysis of large phylogenies. Bioinformatics. 2014;30(9):1312–3. 10.1093/bioinformatics/btu033 24451623 PMC3998144

[48] Larkin MA, Blackshields G, Brown NP, Chenna R, McGettigan PA, McWilliam H, et al Clustal W and Clustal X version 2.0. Bioinformatics. 2007;23(21):2947–8. 17846036 10.1093/bioinformatics/btm404

[49] Lartillot N, Philippe H. A Bayesian mixture model for across-site heterogeneities in the amino-acid replacement process. Mol Biol Evol. 2004;21(6):1095–109. 15014145 10.1093/molbev/msh112

[50] Hulpiau P, van Roy F. New insights into the evolution of metazoan cadherins. Mol Biol Evol. 2011;28(1):647–57. 10.1093/molbev/msq233 20817718

[51] Baumgartner S, Littleton JT, Broadie K, Bhat MA, Harbecke R, Lengyel JA, et al A *Drosophila* neurexin is required for septate junction and blood-nerve barrier formation and function. Cell. 1996;87(6):1059–68. 8978610 10.1016/s0092-8674(00)81800-0

[52] Magie CR, Martindale MQ. Cell-cell adhesion in the Cnidaria: Insights into the evolution of tissue morphogenesis. Biol Bull. 2008;214(3):218–32. 18574100 10.2307/25470665

[53] Chapman JA, Kirkness EF, Simakov O, Hampson SE, Mitros T, Weinmaier T, et al The dynamic genome of *Hydra*. Nature. 2010;464(7288):592–6. 10.1038/nature08830 20228792 PMC4479502

[54] Ganot P, Zoccola D, Tambutté E, Voolstra CR, Aranda M, Allemand D, et al Structural molecular components of septate junctions in cnidarians point to the origin of epithelial junctions in eukaryotes. Mol Biol Evol. 2015;32(1):44–62. 10.1093/molbev/msu265 25246700

[55] Gunaratne HJ, Moy GW, Kinukawa M, Miyata S, Mah SA, Vacquier VD. The 10 sea urchin receptor for egg jelly proteins (SpREJ) are members of the polycystic kidney disease-1 (PKD1) family. BMC Genomics. 2007;8. 10.1186/1471-2164-8-235 PMC1934368 17629917

[56] Hughes J, Ward CJ, Aspinwall R, Butler R, Harris PC. Identification of a human homologue of the sea urchin receptor for egg jelly: a polycystic kidney disease-like protein. Hum Mol Genet. 1999;8(3):543–9. 9949214 10.1093/hmg/8.3.543

[57] Mengerink KJ, Moy GW, Vacquier VD. suREJ3, a polycystin-1 protein, is cleaved at the GPS domain and localizes to the acrosomal region of sea urchin sperm. J Biol Chem. 2002;277(2):943–8. 11696547 10.1074/jbc.M109673200

[58] Qian F, Boletta A, Bhunia AK, Xu HX, Liu LJ, Ahrabi AK, et al Cleavage of polycystin-1 requires the receptor for egg jelly domain and is disrupted by human autosomal-dominant polycystic kidney disease 1-associated mutations. Proc Natl Acad Sci U S A. 2002;99(26):16981–6. 12482949 10.1073/pnas.252484899 PMC139255

[59] Jovine L, Darie CC, Litscher ES, Wassarman PM. Zona pellucida domain proteins. Annu Rev Biochem. 2005;74:83–114. 15952882 10.1146/annurev.biochem.74.082803.133039

[60] Boja ES, Hoodbhoy T, Fales HM, Dean J. Structural characterization of native mouse zona pellucida proteins using mass spectrometry. J Biol Chem. 2003;278(36):34189–202. 12799386 10.1074/jbc.M304026200

[61] Yonezawa N, Nakano M. Identification of the carboxyl termini of porcine zona pellucida glycoproteins ZPB and ZPC. Biochem Biophys Res Commun. 2003;307(4):877–82. 12878193 10.1016/s0006-291x(03)01297-x

[62] Hosaka M, Nagahama M, Kim WS, Watanabe T, Hatsuzawa K, Ikemizu J, et al Arg-X-Lys/Arg-Arg motif as a signal for precursor cleavage catalyzed by furin within the constitutive secretory pathway. J Biol Chem. 1991;266(19):12127–30. 1905715

[63] Vashchenko G, MacGillivray RTA. Multi-copper oxidases and human iron metabolism. Nutrients. 2013;5(7):2289–313. 10.3390/nu5072289 23807651 PMC3738974

[64] Chen HJ, Attieh ZK, Syed BA, Kuo YM, Stevens V, Fuqua BK, et al Identification of zyklopen, a new member of the vertebrate multicopper ferroxidase family, and characterization in rodents and human cells. J Nutr. 2010;140(10):1728–35. 10.3945/jn.109.117531 20685892 PMC2937573

[65] Vasin A, Klotchenko S, Puchkova L. Phylogenetic analysis of six-domain multi-copper blue proteins. PLOS Currents Tree of Life. 2013. 10.1371/currents.tol.574bcb0f133fe52835911abc4e296141 PMC3600357 23516668

[66] Wilson JM, Colton TL. Targeting of an intestinal apical endosomal protein to endosomes in nonpolarized cells. J Cell Biol. 1997;136(2):319–30. 9015303 10.1083/jcb.136.2.319 PMC2134826

[67] Vergnes L, Lee JM, Chin RG, Auwerx J, Reue K. Diet1 functions in the FGF15/19 enterohepatic signaling axis to modulate bile acid and lipid levels. Cell Metabolism. 2013;17(6):916–28. 10.1016/j.cmet.2013.04.007 23747249 PMC3956443

[68] Desseyn JL, Tetaert D, Gouyer V. Architecture of the large membrane-bound mucins. Gene. 2008;410(2):215–22. 10.1016/j.gene.2007.12.014 18242885

[69] Shikina S, Chen C-J, Chung Y-J, Shao Z-F, Liou J-Y, Tseng H-P, et al Yolk Formation in a Stony Coral Euphyllia ancora (Cnidaria, Anthozoa): Insight Into the Evolution of Vitellogenesis in Nonbilaterian Animals. Endocrinology. 2013;154(9):3447–59. 10.1210/en.2013-1086 23766130

[70] Hayakawa H, Andoh T, Watanabe T. Identification of a Novel Yolk Protein in the Hermatypic Coral *Galaxea fascicularis*. Zoolog Sci. 2007;24(3):249–55. 17551245 10.2108/zsj.24.249

[71] Fukuda I, Ooki S, Fujita T, Murayama E, Nagasawa H, Isa Y, et al Molecular cloning of a cDNA encoding a soluble protein in the coral exoskeleton. Biochem Biophys Res Commun. 2003;304(1):11–7. 12705876 10.1016/s0006-291x(03)00527-8

[72] Shinzato C, Iguchi A, Hayward DC, Technau U, Ball EE, Miller DJ. Sox genes in the coral *Acropora millepora*: divergent expression patterns reflect differences in developmental mechanisms within the Anthozoa. BMC Evol Biol. 2008;8. 10.1186/1471-2148-8-311 PMC2613919 19014479

[73] Oda H, Takeichi M. Structural and functional diversity of cadherin at the adherens junction. J Cell Biol. 2011;193(7):1137–46. 10.1083/jcb.201008173 21708975 PMC3216324

[74] Abedin M, King N. The premetazoan ancestry of cadherins. Science. 2008;319(5865):946–8. 10.1126/science.1151084 18276888

[75] McCrea PD, Gu DM. The catenin family at a glance. J Cell Sci. 2010;123(5):637–42. 20164302 10.1242/jcs.039842 PMC2823574

[76] van Roy F, Berx G. The cell-cell adhesion molecule E-cadherin. Cell Mol Life Sci. 2008;65(23):3756–88. 10.1007/s00018-008-8281-1 18726070 PMC11131785

[77] Gumbiner BM. Regulation of cadherin-mediated adhesion in morphogenesis. Nat Rev Mol Cell Biol. 2005;6(8):622–34. 16025097 10.1038/nrm1699

[78] Domart-Coulon I, Tambutté S, Tambutté E, Allemand D. Short term viability of soft tissue detached from the skeleton of reef-building corals. J Exp Mar Bio Ecol. 2004;309(2):199–217.

[79] Steinhusen U, Weiske J, Badock V, Tauber R, Bommert K, Huber O. Cleavage and shedding of E-cadherin after induction of apoptosis. J Biol Chem. 2001;276(7):4972–80. 11076937 10.1074/jbc.M006102200

[80] Marambaud P, Shioi J, Serban G, Georgakopoulos A, Sarner S, Nagy V, et al A presenilin‐1/γ‐secretase cleavage releases the E‐cadherin intracellular domain and regulates disassembly of adherens junctions. The EMBO Journal. 2002;21(8):1948–56. 11953314 10.1093/emboj/21.8.1948 PMC125968

[81] Musci G, Bonaccorsi di Patti MC, Petruzzelli R, Giartosio A, Calabrese L. Divalent cation binding to ceruloplasmin. Biometals. 1996;9(1):66–72. 8574094 10.1007/BF00188092

[82] Muscatine L, Tambutte E, Allemand D. Morphology of coral desmocytes, cells that anchor the calicoblastic epithelium to the skeleton. Coral Reefs. 1997;16(4):205–13.

[83] Goldberg WM. Desmocytes in the calicoblastic epithelium of the stony coral *Mycetophyllia reesi* and their attachment to the skeleton. Tissue Cell. 2001;33(4):388–94. 11521955 10.1054/tice.2001.0192

[84] Barneah O, Malik Z, Benayahu Y. Attachment to the substrate by soft coral fragments: desmocyte development, structure, and function. Invertebr Biol. 2002;121(2):81–90.

[85] Hare PE. Amino acids in proteins from aragonite and calcite in shells of *Mytilus californianus*. Science. 1963;139(355):216–&. 13952795 10.1126/science.139.3551.216

[86] Weiner S, Hood L. Soluble protein of the organic matrix of mollusk shells: a potential template for shell formation. Science. 1975;190(4218):987–9. 1188379 10.1126/science.1188379

[87] Sarashina I, Endo K. Skeletal matrix proteins of invertebrate animals: Comparative analysis of their amino acid sequences. Paleontological Research. 2006;10(4):311–36.

[88] Gorski JP. Acidic phosphoproteins from bone-matrix—a structural rationalization of their role in biomineralization. Calcif Tissue Int. 1992;50(5):391–6. 1596774 10.1007/BF00296767

[89] Kawasaki K, Buchanan AV, Weiss KM. Biomineralization in humans: Making the hard choices in life. Annu Rev Genet. 2009;43:119–42. 10.1146/annurev-genet-102108-134242 19659443

[90] Drake JL, Mass T, Falkowski PG. The evolution and future of carbonate precipitation in marine invertebrates: Witnessing extinction or documenting resilience in the Anthropocene? Elementa Science of the Anthropocene. 2014;2:000026.

[91] Isowa Y, Sarashina I, Setiamarga DHE, Endo K. A comparative study of the shell matrix protein Aspein in pterioid bivalves. J Mol Evol. 2012;75(1–2):11–8. 10.1007/s00239-012-9514-3 22922907

[92] Cuif JP, Dauphin Y, Freiwald A, Gautret P, Zibrowius H. Biochemical markers of zoaxanthellae symbiosis in soluble matrices of skeleton of 24 Scleractinia species. Comp Biochem Physiol A Mol Integr Physiol. 1999;123(3):269–78.

[93] Gautret P, Cuif JP, Freiwald A. Composition of soluble mineralizing matrices in zooxanthellate and non-zooxanthellate scleractinian corals: Biochemical assessment of photosynthetic metabolism through the study of a skeletal feature. Facies. 1997;36:189–94.

[94] Reyes-Bermudez A, Lin Z, Hayward DC, Miller DJ, Ball EE. Differential expression of three galaxin-related genes during settlement and metamorphosis in the scleractinian coral *Acropora millepora*. BMC Evol Biol. 2009;9:178 10.1186/1471-2148-9-178 19638240 PMC2726143

[95] Miyamoto H, Endo H, Hashimoto N, Limura K, Isowa Y, Kinoshita S, et al The diversity of shell matrix proteins: genome-wide investigation of the pearl oyster, *Pinctada fucata*. Zoolog Sci. 2013;30(10):801–16. 10.2108/zsj.30.801 24125645

[96] Kawasaki K, Buchanan AV, Weiss KM. Gene duplication and the evolution of vertebrate skeletal mineralization. Cells Tissues Organs. 2007;186(1):7–24. 17627116 10.1159/000102678

