# Supplementary material for: Stepwise Evolution of Coral Biomineralization Revealed with Genome-Wide Proteomics and Transcriptomics
Source: PLoS One. 2016 Jun 2;11(6):e0156424. doi: 10.1371/journal.pone.0156424 (PMC4890752; doi:10.1371/journal.pone.0156424)
Supplement: S1 Fig — Acid soluble matrix (ASM) and acid insoluble matrix (AIM) fractions of the skeleton stained by silver staining. Arrowheads indicate visible bands. (PDF) [file pone.0156424.s002.pdf]

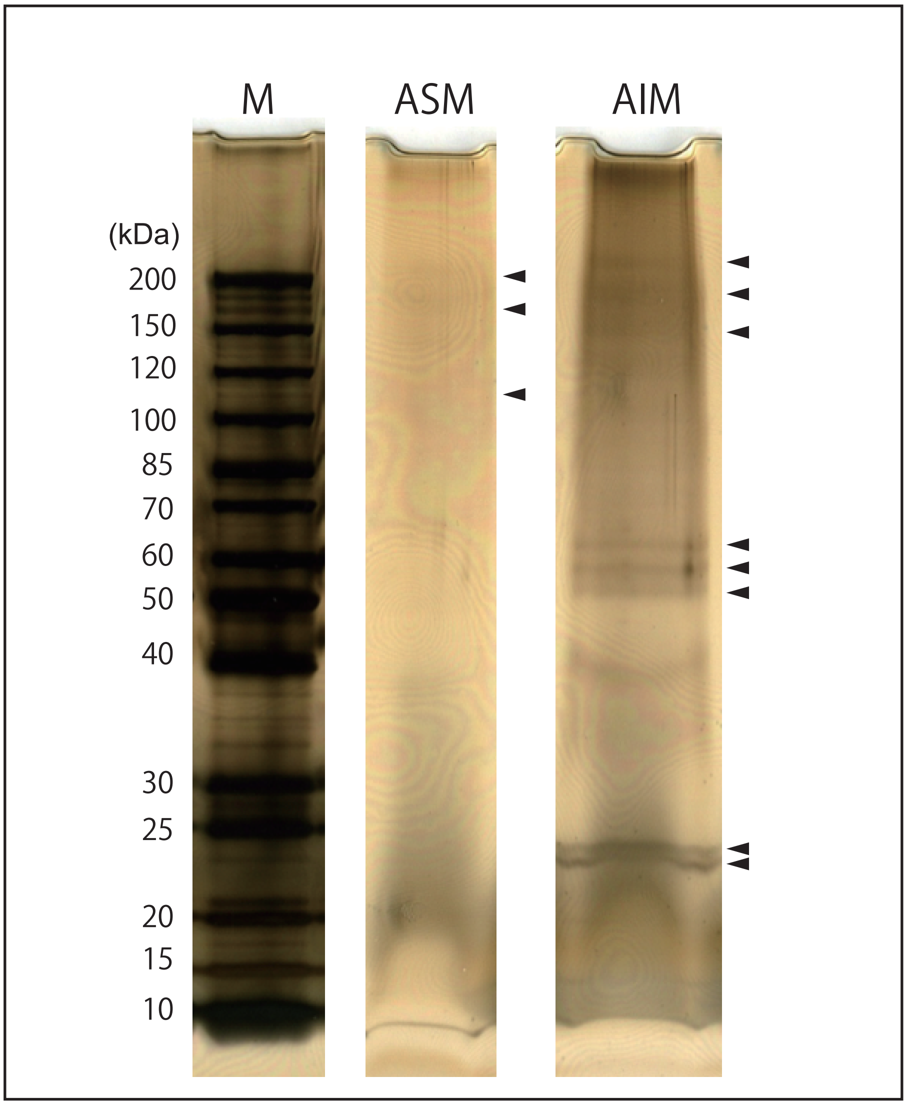

S1 Fig. SDS-PAGE analysis of the ASM and AIM fractions of the skeleton. Acid soluble matrix (ASM) and acid insoluble matrix (AIM) fractions of the skeleton stained by silver staining. Arrowheads indicate visible bands.
