# Supplementary material for: Stepwise Evolution of Coral Biomineralization Revealed with Genome-Wide Proteomics and Transcriptomics
Source: PLoS One. 2016 Jun 2;11(6):e0156424. doi: 10.1371/journal.pone.0156424 (PMC4890752; doi:10.1371/journal.pone.0156424)
Supplement: S2 Fig — The majority of transmembrane proteins are found in all three proteomes. The difference between proteomes of two closely related species, Acropora digitifera and A. millepora, is presumably due to technical issues. (PDF) [file pone.0156424.s003.pdf]

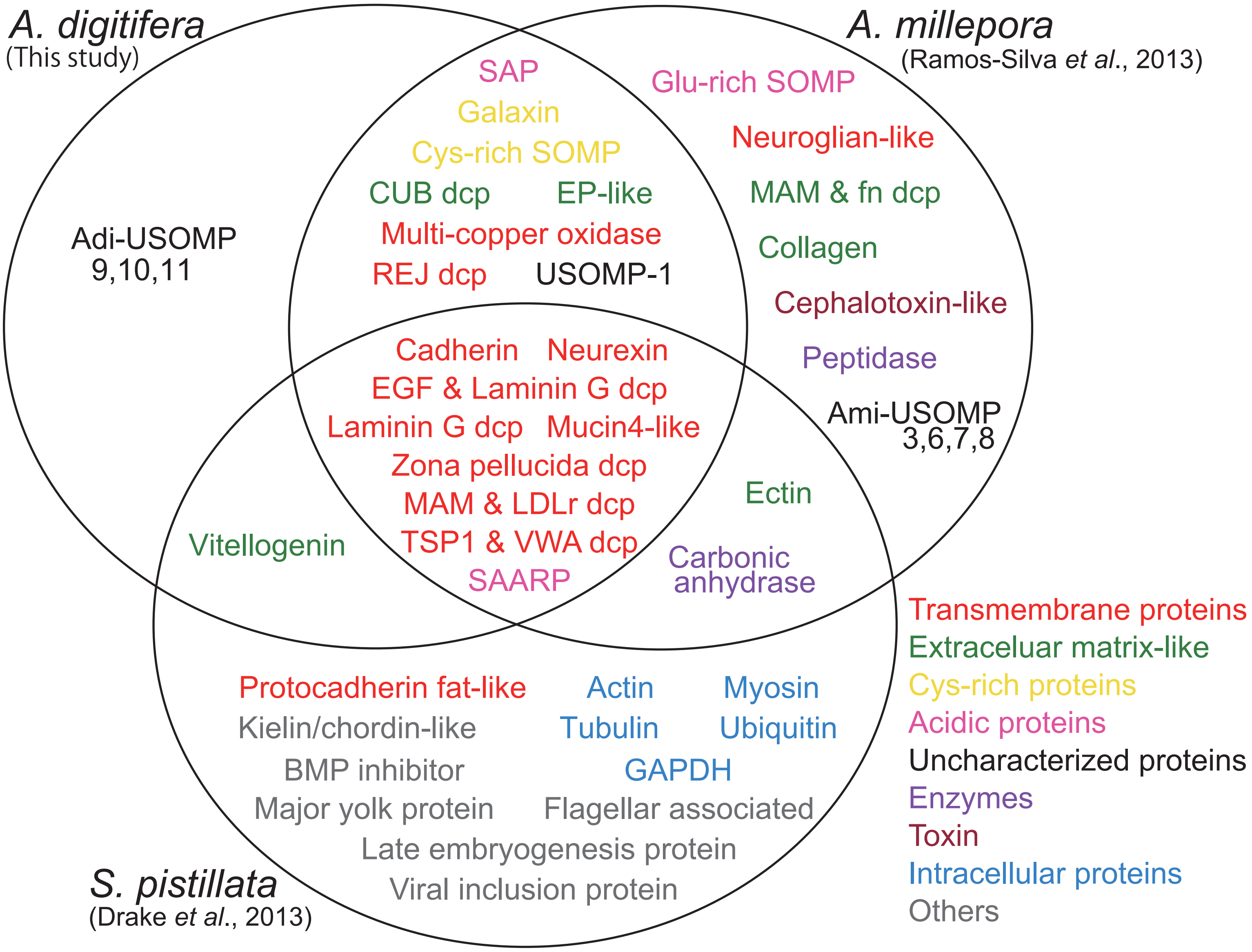

S2 Fig. Venn diagram comparing SOMPs identified in three coral species. The majority of transmembrane proteins are found in all three proteomes. The difference between proteomes of two closely related species, *Acropora digitifera* and *A. millepora,* is presumably be due to technical issues.
