# Supplementary material for: Stepwise Evolution of Coral Biomineralization Revealed with Genome-Wide Proteomics and Transcriptomics
Source: PLoS One. 2016 Jun 2;11(6):e0156424. doi: 10.1371/journal.pone.0156424 (PMC4890752; doi:10.1371/journal.pone.0156424)
Supplement: S3 Fig — (a) Schematic domain structure of cadherin proteins. The A. digitifera cadherin SOMP has a domain structure typical of non-chordate metazoans. Lengths of amino acid sequences are shown at the right. (b) Alignment of amino acid sequences in the cytoplasmic domain of cadherins. A. digitifera cadherin SOMP retains conserved motifs of p120-catenin and β-catenin-binding sequences. Conserved amino acid positions are highlighted with blue. Transcript ID, gene model ID, and NCBI accession ID of the proteins are as follows: A. digitifera cadherin (adi_EST_assem_2804), N. vectensis CDH1 (XP_001631293.1), D. melanogaster cadherin-N (NP_001027277.1), T. adhaerens TaCDH (Triad1|55710), A. millepora cadherin (JT011093), and M. musculus Cad-1 (NP_033994.1). (PDF) [file pone.0156424.s004.pdf]

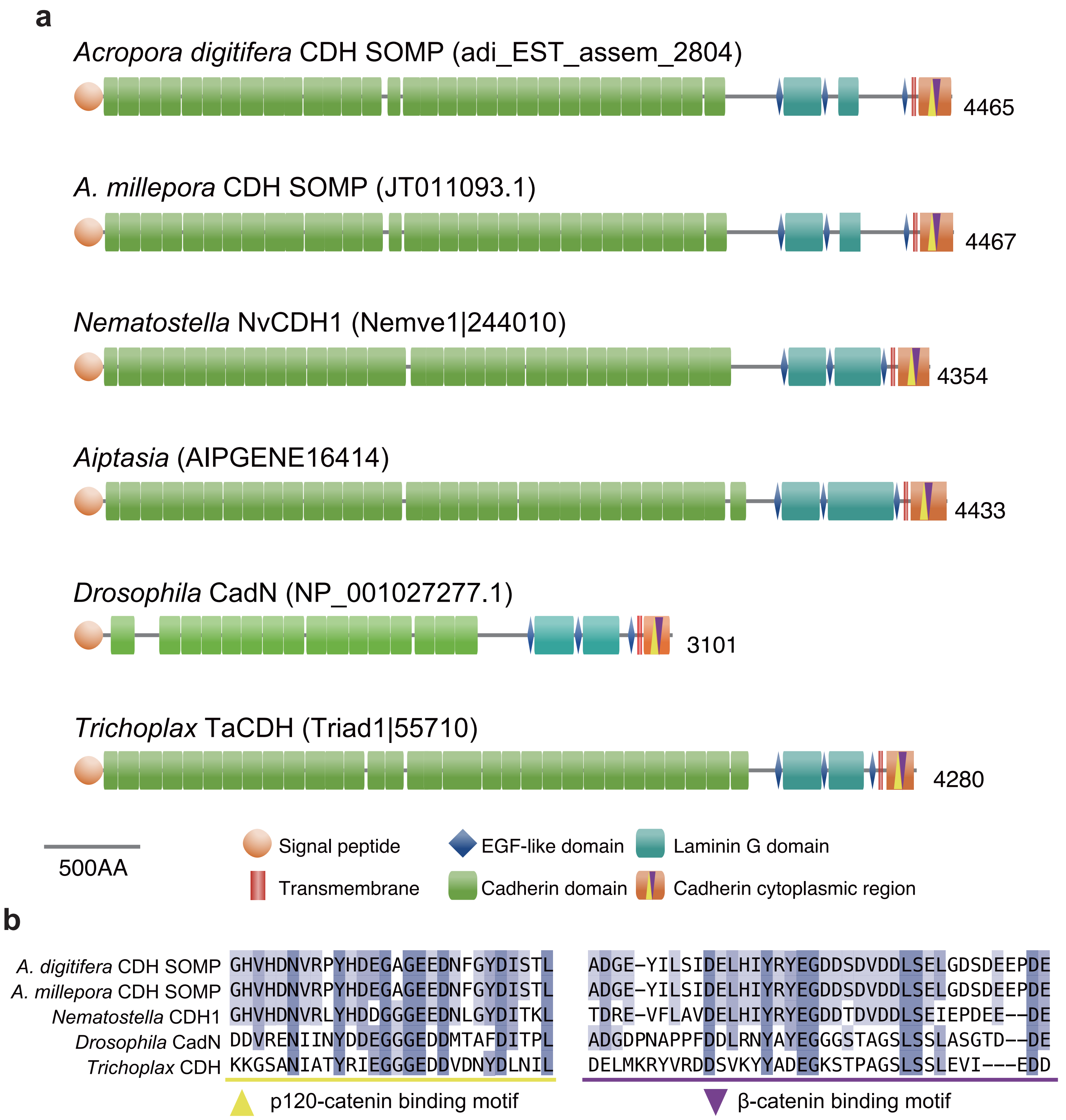

S3 Fig. Structure of the cadherin SOMP of *Acropora digitifera* and cadherins of other representative animals. (a) Schematic domain structure of cadherin proteins. The *A. digitifera* cadherin SOMP has a domain structure typical of non-chordate metazoans. Lengths of amino acid sequences are shown at the right. (b) Alignment of amino acid sequences in the cytoplasmic domain of cadherins. *A. digitifera* cadherin SOMP retains conserved motifs of p120-catenin and β-catenin-binding sequences. Conserved amino acid positions are highlighted with blue. Transcript ID, gene model ID, and NCBI accession ID of the proteins are as follows: *A. digitifera* cadherin (adi_EST_assem_2804), *N. vectensis* CDH1 (XP_001631293.1), *D. melanogaster* Cadherin-N (NP_001027277.1), *T. adhaerens* TaCDH (Triad1|55710) , and *A. millepora* cadherin (JT011093).
