# Supplementary material for: Stepwise Evolution of Coral Biomineralization Revealed with Genome-Wide Proteomics and Transcriptomics
Source: PLoS One. 2016 Jun 2;11(6):e0156424. doi: 10.1371/journal.pone.0156424 (PMC4890752; doi:10.1371/journal.pone.0156424)
Supplement: S4 Fig — (a) Domain architecture of neurexin is conserved among cnidarian species. (b) EGF-like and laminin G dcps. (c) Laminin G dcp. Lengths of amino acid sequences are shown at the right. (PDF) [file pone.0156424.s005.pdf]

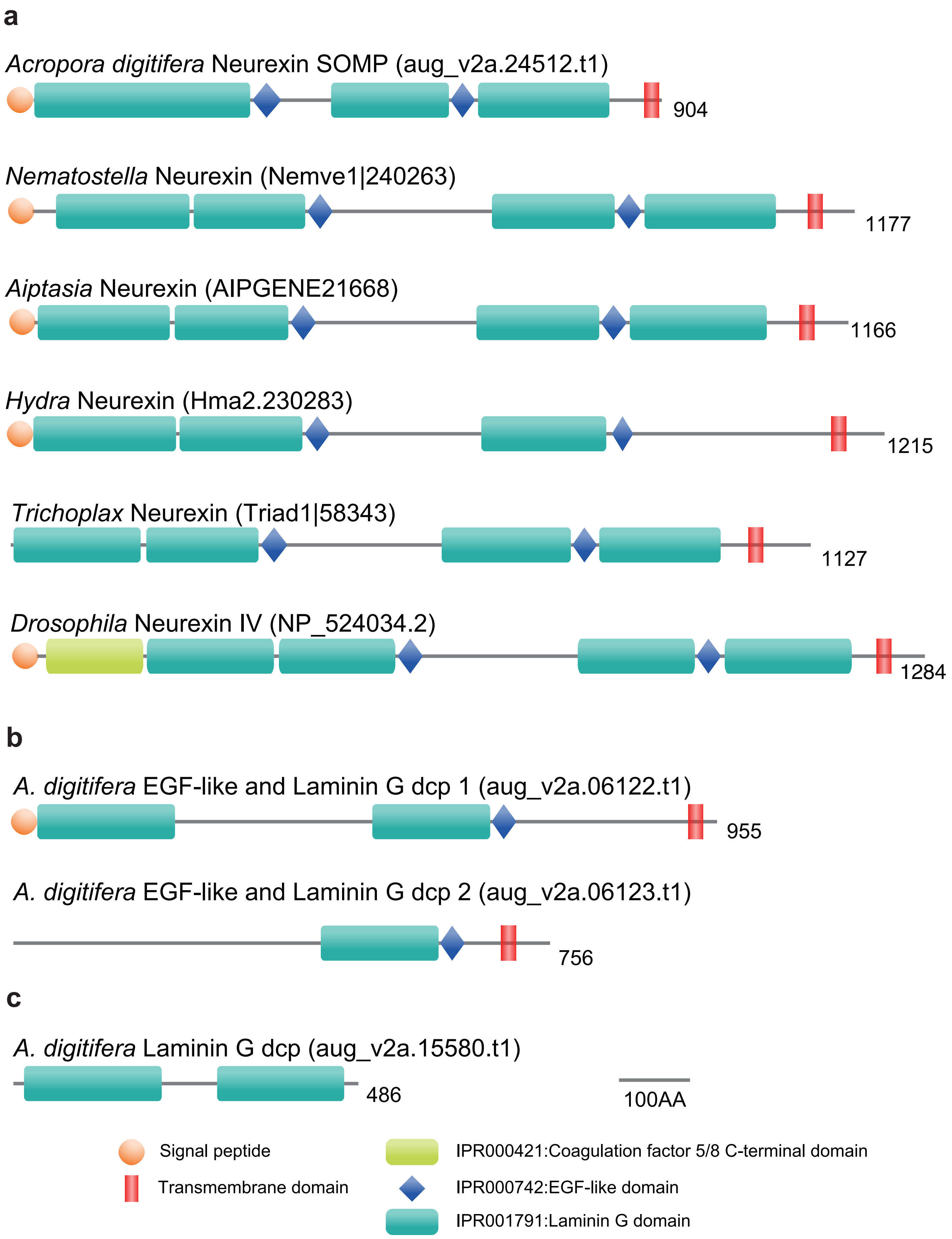

S4 Fig. Neurexin protein architectures of selected animals and related proteins of *A. digitifera*. (a) Domain architecture of neurexin is conserved among cnidarian species. (b) EGF-like and laminin G dcps. (c) Laminin G dcp. Lengths of amino acid sequences are shown at the right.
