# Supplementary material for: Stepwise Evolution of Coral Biomineralization Revealed with Genome-Wide Proteomics and Transcriptomics
Source: PLoS One. 2016 Jun 2;11(6):e0156424. doi: 10.1371/journal.pone.0156424 (PMC4890752; doi:10.1371/journal.pone.0156424)
Supplement: S5 Fig — A. digitifera neurexin (aug_v2a.24512), EGF-like and laminin G dcps (aug_v2a.06122, aug_v2a.06123), laminin G dcp (aug_v2a.15580), and A. millepora EGF and laminin G dcp (JR980881.1) are aligned. Conserved residues are highlighted with blue and the transmembrane domain is underlined with purple. (PDF) [file pone.0156424.s006.pdf]

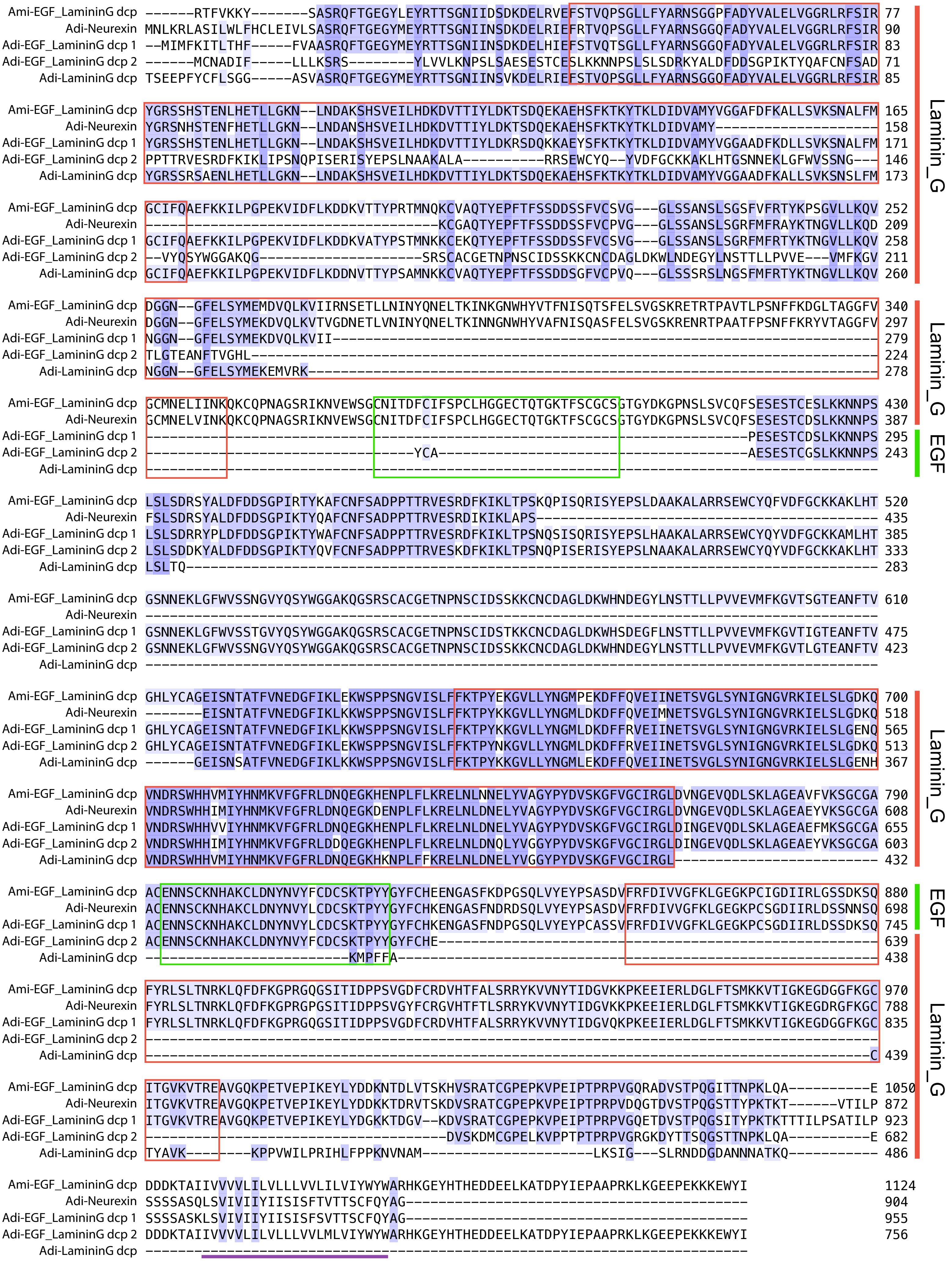

S5 Fig. Alignment of coral neurexin and related proteins. *A. digitifera* neurexin (aug_v2a.24512), EGF-like and laminin G dcps (aug_v2a.06122, aug_v2a.06123), laminin G dcp (aug_v2a.15580), andd *A. millepora* EGF and laminin G dcp (JR980881.1) are aligned. Conserved residues are highlighted with blue and the transmembrane domain is underlined with purple.
