# Supplementary material for: Stepwise Evolution of Coral Biomineralization Revealed with Genome-Wide Proteomics and Transcriptomics
Source: PLoS One. 2016 Jun 2;11(6):e0156424. doi: 10.1371/journal.pone.0156424 (PMC4890752; doi:10.1371/journal.pone.0156424)
Supplement: S6 Fig — (a) The conserved domain structure of PKD1 proteins in a coral and representative animals. The lengths of amino acid sequences are shown at the right. (b) Sequence alignment of GPS domains. The arrow indicates the putative cleavage site of the domain. Gene model IDs or NCBI accession IDs of the proteins are as follows: A. digitifera (aug_v2a.02830.t1, adi_EST_assem_6849), N. vectensis (Nmeve1|196807), S. purpuratus SpREJ3 (AAL26499.1), and H. sapiens PC1 (NP_001009944.2). (PDF) [file pone.0156424.s007.pdf]

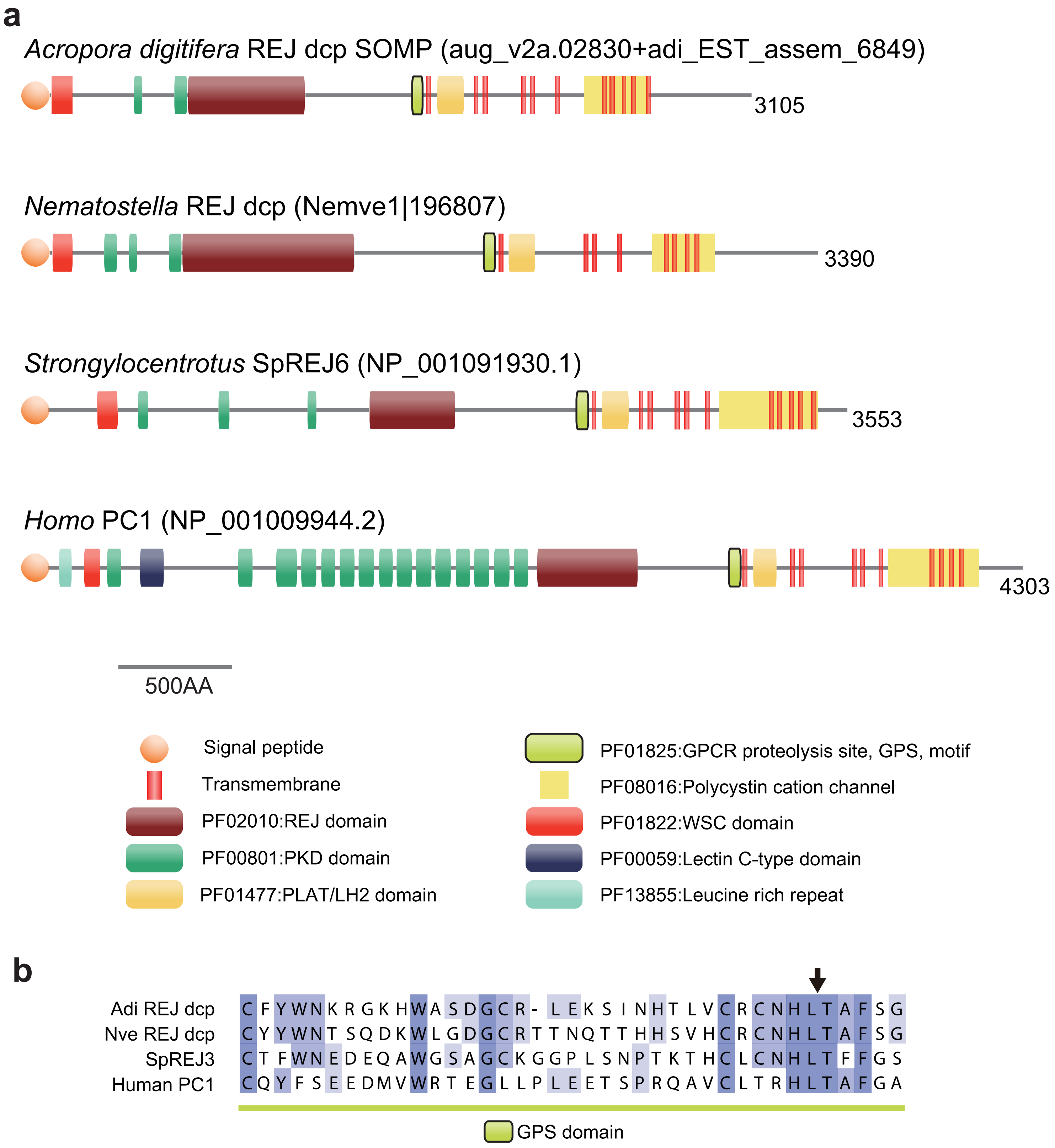

S6 Fig. Domain structure of REJ domain-containing proteins. (a) The conserved domain structure of REJ domain-containing proteins in *A. digitifera* and representative animals. Lengths of amino acid sequences are shown at the right. (b) Sequence alignment of GPS domains. The arrow indicates the putative cleavage site of the domain. Gene model IDs or NCBI accession IDs of the proteins are as follows: *A. digitifera* (adi_EST_assem_6849), *N. vectensis* (Nemve1|196807), *S. purpuratus* SpREJ3 (AAL26499.1), and *H. sapiens* PC1 (NP_001009944.2).
