# Supplementary material for: Stepwise Evolution of Coral Biomineralization Revealed with Genome-Wide Proteomics and Transcriptomics
Source: PLoS One. 2016 Jun 2;11(6):e0156424. doi: 10.1371/journal.pone.0156424 (PMC4890752; doi:10.1371/journal.pone.0156424)
Supplement: S7 Fig — Conserved cysteine residues in the ZP domain are marked by asterisks. The ZP domain underlined with red is immediately followed by a putative proteolytic cleavage site (Arg-X-Lys/Arg-Arg) underlined with green. The transmembrane domain is underlined with purple. Conserved amino acid positions are highlighted with blue. The transcriptome IDs, gene model IDs, or NCBI accession IDs of the proteins are as follows: A. digitifera ZP dcp N-terminus (adi_EST_assem_1474), C-terminus (adi_EST_2269), A. millepora (AET09743.1), N. vectensis (Nemve1|204835), and Aiptasia pallida (JV132371.1). (PDF) [file pone.0156424.s008.pdf]

```
Adi_ZP_dcp-N     1 MFLYSFVFLMLLGLSSAQTESATSPDEVETEPTMSTDQPETSPSCVNSIRTNDRNATSNHSTTP
 Ami_ZP_dcp      1 MFLYSFVFLMLLGLSSAQTESATSPDEVETEPTMSTDQPETSPSM-------------STETEP
 Nve_ZP_dcp      1 ---MTAAPMETTAAPDATT---AAPDATTAAPEATTAAPEATTAAPEA-------TTAAPVETT
 Apa_ZP_dcp      1 ---MARILVLFMCLAVLHV---AVAQDNKTDPS-------------------------------

Adi_ZP_dcp-C    65 TTETPPVTTPPPPDSLSVICTNEKMEVFLDHTKHDNLDLDKVTLKDANCKASGTLNATHLWMDV
 Ami_ZP_dcp     52 TTETPPVTTPPPPDSLSVICTNEKMEVFLDHAKHDNLDLDKVTLKDANCKASGTLNATHLWMDV
 Nve_ZP_dcp     52 AGPTEAPTTRPEAKGLFVTCTAQEIQIKLDIKEHSGLQLDTLTLKDTNCQVYKN-DGQFAYFRS
 Apa_ZP_dcp     28 -------------KGMSVTCTSEYIEAVIDTSVHKDLQPDRLTLEDKSCTLTKT-EGTLVYFRT
                                      *                            *

Adi_ZP_dcp-C   129 PFDSCMTNHSTDGDTITYQNSLVAETRASAGSSLISREFQAEFPFKCTYPRSVVLSVVAFSPRE
 Ami_ZP_dcp    116 PFDSCMTNHSTDGDTITYQNSLVAETRASAGSSLISREFQAEFPFKCTYPRSAVLSVVAFSPRE
 Nve_ZP_dcp    115 PLDGCGTTHNTTKTHIKYMNAIRAETTAKTNNAKISRDFQAEFPVQCTYERSAILSVVSFSPRK
 Apa_ZP_dcp     78 ALDGCGTKHNTTDDDIVYYNSIFGETNAGSNSAKISRKHQAEYPFKCTFPRSKILSVVNFSPRR
                       *                                         *

Adi_ZP_dcp-C   193 RIVYTKTAEFGNFTFTMDMYKTDKYET-PYEAFPVRLDLDDPMFLEVKVSSNDSKLVLIPLKCW
 Ami_ZP_dcp    180 RIVYTKTAEFGNFTFTMDMYKTDKYET-PYDSFPVRLDLDDPMFLEVKVSSNDSKLVLIPLKCW
 Nve_ZP_dcp    179 KVVYSSASGLGNFTYEMSLLKGDNAESDEITEFPHVVDLNAFLNIRASIKSNDSQLSLFIDNCW
 Apa_ZP_dcp    142 KVIYSRTADYGNFTFEMDLYKSDKYEE-AYEDYPIDVALNSWLYVQTSVSSNDSKLALFNEKCW
                                                                                 *

Adi_ZP_dcp-C   256 ATPSSDLQDDKYYAFIENGCGKADDPSLVFNYKESN-VQRFKMGAFRFIGESLNSNVYLHCDVE
 Ami_ZP_dcp    243 ATPSSDLQDDKYYTFIENGCGKADDPSLVFNYGESN-VQRFKIGAFRFIGESLNSNVYLHCDVE
 Nve_ZP_dcp    243 ATPTSDPKYKNQHKIITTGCKK--DSTVQYDYKTSKPIQFFRMRSFRFLSEN-SDNIHIHCEVE
 Apa_ZP_dcp    205 ATPSSDSGDKEQFVFIDKGCQKEKKEDMEYDFKKNKPEQQFQLKAFRFLTGS-GNTVYLHCRVE
                                      *                                         *

Adi_ZP_dcp-C   319 ACRKGDSDSRCAKGCETSRRRRRSSLASSAGTEQTVTLGPMKISEKEEVGAQEA--VSSLTIFA
 Ami_ZP_dcp    306 ACRKGDSDSRCAKGCETSRRRRRSSLASSAGTEQTVTLGPMKISEKAEVGAQEA--VSSLTIFA
 Nve_ZP_dcp    304 ACRADDKDSNCAKGCQPSSRKRRSLYSDASST--LLTIGSVKVVSDANAAQSTNANASGLTTVS
 Apa_ZP_dcp    268 VCREGDKDSGCSKGCEGVDRRRRRSLVTDASSE-VLTVGVVKVVKGSNAQSASAGNASSLTTIS
                    *        *   *

Adi_ZP_dcp-C   381 AVAGVLGVIVLFLAVALVMLYKRYRSPQSATRVVYTKTANEEGKLLV-
 Ami_ZP_dcp    368 AVAGVLGVIVLFLAVALVMLYKRYRSPQSATRVVYTKTANEEGKLLV-
 Nve_ZP_dcp    366 IVAGVLAVLVIGLVAALVIIHRR-RQQAVNRPVAFKRVQGDTEDLLI-
 Apa_ZP_dcp    331 IVAGILGVLVVALVAVLAVVYKR-R-QAVPVNATFKKVAGNDGDQLMA
```

S7 Fig. Sequence alignment of cnidarian zona pellucida (ZP) domain-containing proteins. Conserved cysteine residues in the ZP domain are marked by asterisks. The ZP domain underlined with red is immediately followed by a putative proteolytic cleavage site (Arg-X-Lys/Arg-Arg) underlined with green. The transmembrane domain is underlined with purple. Conserved amino acid positions are highlighted with blue. The transcriptome IDs, gene model IDs, or NCBI accession IDs of the proteins are as follows: *A. digitifera* ZP dcp N-terminus (adi_EST_assem_1474), C-terminus (adi_EST_2269), *A. millepora* (AET09743.1), *N. vectensis* (Nemve1|204835), and *Aiptasia pallida* (JV132371.1).
