# Supplementary material for: Stepwise Evolution of Coral Biomineralization Revealed with Genome-Wide Proteomics and Transcriptomics
Source: PLoS One. 2016 Jun 2;11(6):e0156424. doi: 10.1371/journal.pone.0156424 (PMC4890752; doi:10.1371/journal.pone.0156424)
Supplement: S8 Fig — Cu-binding histidine residues are highlighted in cyan for type I, pink for type II, and yellow for type III. Metal-binding residues are highlighted in green. Transmembrane domains are shaded in purple. Cupredoxin domains inferred from mouse Hephaestin are boxed in red for the 1st, 3rd, and 5th, and blue for the 2nd, 4th, and 6th, respectively. Transcriptome IDs or NCBI accession IDs of the proteins are as follows: A. digitifera MCO N-terminus (adi_EST_assem_20166), C-terminus (adi_EST_assem_13507), M. musculus Cp (NP_001263177.1), Heph (NP_001153099.1), and Hephl (NP_001158269.1). (PDF) [file pone.0156424.s009.pdf]

```
Adi_MCO-SOMP-N    1 -MMDRSNAAFVLTACFIFSQLICHVAAITRTYYIAAVEKEWDYAPSGYNKIKGVKLEDDSDATVFATKGAHRIGR
Mmu_Heph          1 ----MKAGHLLWALLLMHSLWSIPTDGAIRNYYLGIQDMQWNYAPKGRNVITNQTLNNDTVASSFLKSGKNRIGS
Mmu_Hephl         1 MFLKQPGGCILLQFLGLLGL----VGAVTRTYYIGIVEEYWNYVPQGKDVITGKSFSEDKLATLFLERGPNRIGG
Mmu_Cp            1 -----MKFLLLSTFIFLYSSLALARD---KHYFIGITEAVWDYA-SGTEEKKLISVDTEQ-SNFYLQNGPDRIGR

Adi_MCO-SOMP-N   75 IYDKVLYREYEDASFTKEKPHPKYLGFLGPILKGEIGDTIVVHFKNNGSRVYSMHPHGVFYSKDSEGALYEDNTK
Mmu_Heph         72 SYKKTVYKEYSDGTYTEEIAKPAWLGFLGPLLQAEVGDVILIHLKNFASRPYTIHPHGVFYEKDSEGSLYPDGSS
Mmu_Hephl        72 IYKKAVYRHFTDGSYSTEIPKPPWLGFLGPILRAEVGDVIVIHLMNFASRPFSLHPHGVFYDKDSEGALYPDGTS
Mmu_Cp           66 KYKKALYFEYTDGTFSKTIDKPAWLGFLGPVIKAEVEDKVYVHLKNLASRIYTFHAHGVTYTKEYEGAVYPDNTT

Adi_MCO-SOMP-N  150 GTFKKDDKVPPGGTHMYTWQLTQSHAPADQEDKCITWIYHSH---------------------------------
Adi_MCO-SOMP-C    1 ----------------------------------------------KDINTGLLGIMLICRKGALNQ---GQQSG
Mmu_Heph        147 GYLKADDSVPPGGSHVYNWSIPESHAPTEADPACLTWIYHSHVDAPRDIATGLIGPLITCKRGTLDGNSPPQRKD
Mmu_Hephl       147 GRNKEDDMVPPGKNYTYVWPVREEYAPAPADANCLTWVYHSHIDAPKDICSGLIGPLLVCKEGVLNRYS-GMRTD
Mmu_Cp          141 DFQRADDKVLPGQQYVYVLHANEP-SPGEGDSNCVTRIYHSHVDAPKDIASGLIGPLILCKKGSLYK---EKEKN
                                                                                  Cupredoxin_1

Adi_MCO-SOMP-C   27 VDKEFVALFTVLDENESWLLSKNIER-CSDPTGVHPNDEDFKESNKMHAINGYFYGNLPGLDMCYGDSVKWHLAG
Mmu_Heph        222 VDHNFFLLFSVIDENLSWHLDDNIATYCSDPASVDKEDGAFQDSNRMHAINGFVFGNLPELSMCAQKHVAWHLFG
Mmu_Hephl       221 VDREFVIMFTLVDENQSWYLDDNIKQFCTNPNSVDKSDAVFQRSNKMHALNGFLFGNMPEPEMCVGESVSWHLFG
Mmu_Cp          212 IDQEFVLMFSVVDENLSWYLEDNIKTFCSEPEKVDKDNEDFQESNRMYSINGYTFGSLPGLSMCAADRVKWYLFG

Adi_MCO-SOMP-C  101 IGNEVDIHTAYFHGQSFTIDGHRKDVASLLPATFVTASMKALNPGKWMLNCLVNDHYNAGMYTLFNVTKCPGKVG
Mmu_Heph        297 MGNEIDVHTAFFHGQMLSIRGHHTDVANIFPATFVTAEMVPQKSGTWLISCEVNSHLRSGMQAFYKVDSCSMDPP
Mmu_Hephl       296 MGNEIDIHSIYFYGNTFITRGHRADVVNLFPATFLTTEMIVENPGKWMITCQVSDHLQAGMLGQYSVGNCRGNAP
Mmu_Cp          287 MGNEVDVHSAFFHGQALTSRNYQTDIINLFPATLIDAYMVAQNPGVWMLSCQNLNHLKAGLQAFFQVRDCNKPSP
                                                                                  Cupredoxin_2

Adi_MCO-SOMP-C  176 VAPSVSGGKKRTYFIAANEVEWNYGPTGVNGMDGQSLIAPGSDSAVFFAQNAQRIGGTYLKAIYEQYTDARFSTK
Mmu_Heph        372 V-DQLTG-KVRQYFIQAHEIQWDYGPIGYDGRTGKSLREPGSGPDKYFQKSSSRIGGTYWKVRYEAFQDETFQER
Mmu_Hephl       371 H-PKVQG-QQRRYFIAAEKVLWDYGPQGYDKFTGFPLNTSGSDSAVYFTQADNRIGGKYWKARYTEYVDATFSRR
Mmu_Cp          362 E-DNIQDRHVRHYYIAAEEVIWNYAPSGTDIFTGENLTALESDSRVFFEQGATRIGGSYKKMAYREYTDGSFTNR

Adi_MCO-SOMP-C  251 ---VSKPEHLGFLGPVIRAEVNDIIEVVFKNNARFNFSIQPHGVFFDKSNEGALYDD-GTSRAQKADDNVQPGQT
Mmu_Heph        445 -VHQEEETHLGILGPVIRAEVGDTIQVVFYNRASQPFSIQPHGVFYEKNSEGTVYND-GTSHPKV----AKSFEK
Mmu_Hephl       444 KMPSDSEAHLGILGPVIKAEVGDILLVTFANKADKVYSILPHGVFYDKASDAAPNVD-GFLKPGA---HVKPGET
Mmu_Cp          436 KQRGPDEEHLGILGPVIWAEVGDTIKVTFHNKGQHPLSIQPMGVSFTAENEGTYYGPPGRSSQQAS--HVAPKET

Adi_MCO-SOMP-C  322 FTYRWTVPEEVGPTKSDAACITWVYHSSVDPVKDTYSGLFGPLLTCKKGTLNNDNTRKDTEKEFVLLFTVTDESE
Mmu_Heph        514 VTYYWTVPPHAGPTAQDPACLTWMYFSAADPTRDTNSGLVGPLLVCKAGALGADGKQKGVDKEFFLLFTVFDENE
Mmu_Hephl       515 FTYRWTVPESVSPTDEDPPCLTYLYFSAVQPIKDTSAGLVGPLLVCKKGTLNADGTQKGIDKEFYLLFTVFDENF
Mmu_Cp          509 FTYEWTVPKEMGPTYADPVCLSKMYYSGVDPTKDIFTGLIGPMKICKKGSLLADGRQKDVDKEFYLFPTVFDENE
                                                                                  Cupredoxin_3

Adi_MCO-SOMP-C  397 SWYHEKN-KEMKANASLINDDDEDYKESNKMHGINGFLYANLPGLEMCLGDTISWHVIGLGNEVDMHTAYFYGNT
Mmu_Heph        589 SWYNNANQAAGMLDSRLLSEDVEGFQDSNRMHAINGFLFSNLPRLDMCKGDTVAWHLLGLGTETDVHGVMFEGNT
Mmu_Hephl       590 SSYLDENIKKFTWHPFSVDKEDKEFVKSNRMHAVNGYMYGSQPGLSMCKKDRVSWHLIGMGTDTDMHGVYFQGNT
Mmu_Cp          584 SLLLDDNIRMFTTAPDQVDKEDEDFQESNKMHSMNGFMYGNQPGLNMCLGESIVWYLFSAGNEADVHGIYFSGNT

Adi_MCO-SOMP-C  471 FTHQGSVKDTVSLLPGVFGTLTMTPDNAGNWALVCRTNDHYSAGMQAKYKVNTC--NRNPELKTPGKTRRYYIAA
Mmu_Heph        664 VQLQGMRKGAVMLFPHTFVTAIMQPDNPGIFEIYCQAGSHREEGMQAIYNVSQCSSHQDSPRQHYQASRVYYIMA
Mmu_Hephl       665 IHLRGTHRDSLALFPHMATTAYMQPDHSGIFKVFCSTLPHFTRGMGQIYEISSCG-NRDPSEPPYGMLRTFFIAA
Mmu_Cp          659 YLSKGERRDTANLFPHKSLTLLMNPDTKGTFDVECLTTDHYTGGMKQKYTVNQCQ-RQFEDFTVYLGERTYYVAA
                                                                                  Cupredoxin_4

Adi_MCO-SOMP-C  544 FEMEWDYAPSGLDALDGKKLDQ--SEEAKVFTVTSDKRIGRKYVKAVYREFTNDQFKQQKLRTPAEEHLGILGPM
Mmu_Heph        739 EEIEWDYCPDRSWELEWHNTSE-KDSYGHVFLSNKDGLLGSKYKKAVFREYTDGTFRIPRPRSGPEEHLGILGPL
Mmu_Hephl       739 EEVEWDYAPNKNWEFEKQHLDAGGERHGDIFMNHTENWIGSQYRKVVYREYTNGEFVEIKARPPQEEHLQLLGPM
Mmu_Cp          733 VEVEWDYSPSRAWEKELHHLQE--QNVSNVFLDKEEFFIGSKYKKVVYRQFTDSSFREQVKRRAEDEHLGILGPP

Adi_MCB-SOMP-C  617 LHAEVGDTIEVVFKNNANHNYSVHPHGLYYSKAHEGSDYNDGTSGADKLDNAIQPGKTYKYIWKVPERAGPGKDG
Mmu_Heph        813 IRGEVGDILTVVFKNKASRPYSIHAHGV-----------LESNTG---GPQAAEPGEVLTYQWNIPERSGPGPSD
Mmu_Hephl       814 IHAEVGDSILIIFKNKASRPYSIAAQGV-----------EDSNNGKLLNVPVTKPGEIKTYRWNVPKRSGPGPSD
Mmu_Cp          806 IHANVGDKVKVVFKNMATRPYSIHAHGV-----------KTESS----TVVPTLPGEVRTYTWQIPERSGAGRED

Adi_MCO-SOMP-C  692 PACATWAYYSDVNPIKDTNSGLIGPLIICKKGKLKEGTEERSDVDREFVLMFTVLDENESWYLDENIKKY-CKNP
Mmu_Heph        874 SACVSWIYYSAVDPIKDMYSGLVGPLVICRNGILEP-NGGRNDMDREFALLFIFDENQSWYLKENIATYGPQES
Mmu_Hephl       878 PNCIPWVYFSTANFVKDTYSGLMGPLITCREGVLNE-KGRRSDVDYEFALLFLVFNENESWYLDDNIKKYLNKDP
Mmu_Cp          866 SACIPWAYYSTVDRVKDLYSGLIGPLIVCRKSYVKV-FSPKKKM--EFFLLFLVFDENESWYLDDNIKTY-SEHP
                                                                                  Cupredoxin_5

Adi_MCO-SOMP-C  766 GDKETLKADDDFMESNKMHGINGFVFGNLRGLKMYQDEKVDWLLLGIGNEVDMHTVHFHGQSFLRKQVSYHREDV
Mmu_Heph        948 SHVN--LKDATFLESNKMHAINGKLYANLRGLTVYQGERVAWYMLAMGQDTDIHTVHFHAESFLYQNGQSYRADV
Mmu_Hephl       952 RDF---KHTDDFEESNKMHAINGKIFGNLPGLIMTEDSMTNWYLLGIGSEVDIHTIHYHAESFLFKIDKSYREDV
Mmu_Cp          937 EKVN--KDNEEFLESNKMHAINGKMFGNLQGLTMHVKDEVNWYVMGMGNEIDLHTVHFHGHSFQYKHRGVYSSDV

Adi_MCO-SOMP-C  841 YDLFPGVFATVEMVPDSTGDWLLHCHVNDHMVAGMETLYSVL---------------------------DKSLKT
Mmu_Heph       1021 VDLFPGTFEVVEMVASNPGTWLMHCHVTDHVHAGMETIFTVLSH-EEHFSTMTTITKEIGKAVIL----RDIGGD
Mmu_Hephl      1024 YDLFPGTFQTIELFADHPGTWLLHCHVSDHIHAGMETTYTVLRNIDNRIPYSTKTPSGAGSHAVTVPSQEQPGKE
Mmu_Cp         1010 FDLFPGTYQTLEMFPQTPGTWLLHCHVTDHVHAGMATTYTVLPV-------------------------EQVSSQ
                                                                                  Cupredoxin_6

Adi_MCO-SOMP-C  889 TLKPITAASSFVTSSIFIYLSFPILAMLLKA------------------------------------
Mmu_Heph       1091 NVKMLGMNIPIKDVEILSSALIAICVLLLLIALALGGVVWYQHRQRKLRRNRRSILDDSFKLLSLKQ---
Mmu_Hephl      1099 ELYFFGKNLRPRGAKAALVILFILGLLLLVATVVLA---------LRLRSSRQMAYREVQSCALPTDAL
Mmu_Cp         1060 SYRM--------TWNILYTLLISIIILFQMSTKE------------------------------------
```

S8 Fig. Alignment of coral multi-copper oxidase (MCO) and mouse MCO. Cu-binding histidine residues are highlighted in cyan for type I, pink for type II, and yellow for type III. Metal binding residues are highlighted in green. Transmembrane domains are shaded in purple. Cupredoxin domains inferred from mouse Hephaestin are boxed in red for the 1st, 3rd, and 5th, and blue for the 2nd, 4th, and 6th, respectively. Transcriptome IDs or NCBI accession IDs of the proteins are as follows: *A. digitifera* MCO N-terminus (adi_EST_assem_20166), C-terminus (adi_EST_assem_13507), *M. musculus* Cp (NP_001263177.1), Heph (NP_001153099.1), and Hephl (NP_001158269.1).
