# Supplementary material for: Stepwise Evolution of Coral Biomineralization Revealed with Genome-Wide Proteomics and Transcriptomics
Source: PLoS One. 2016 Jun 2;11(6):e0156424. doi: 10.1371/journal.pone.0156424 (PMC4890752; doi:10.1371/journal.pone.0156424)
Supplement: S9 Fig — Acropora multi-copper oxidase SOMP is similar to that of Aiptasia. In addition, the domain architecture with six cupredoxin domains is conserved among bilaterian animals. Lengths of amino acid sequences are shown at the right. (PDF) [file pone.0156424.s010.pdf]

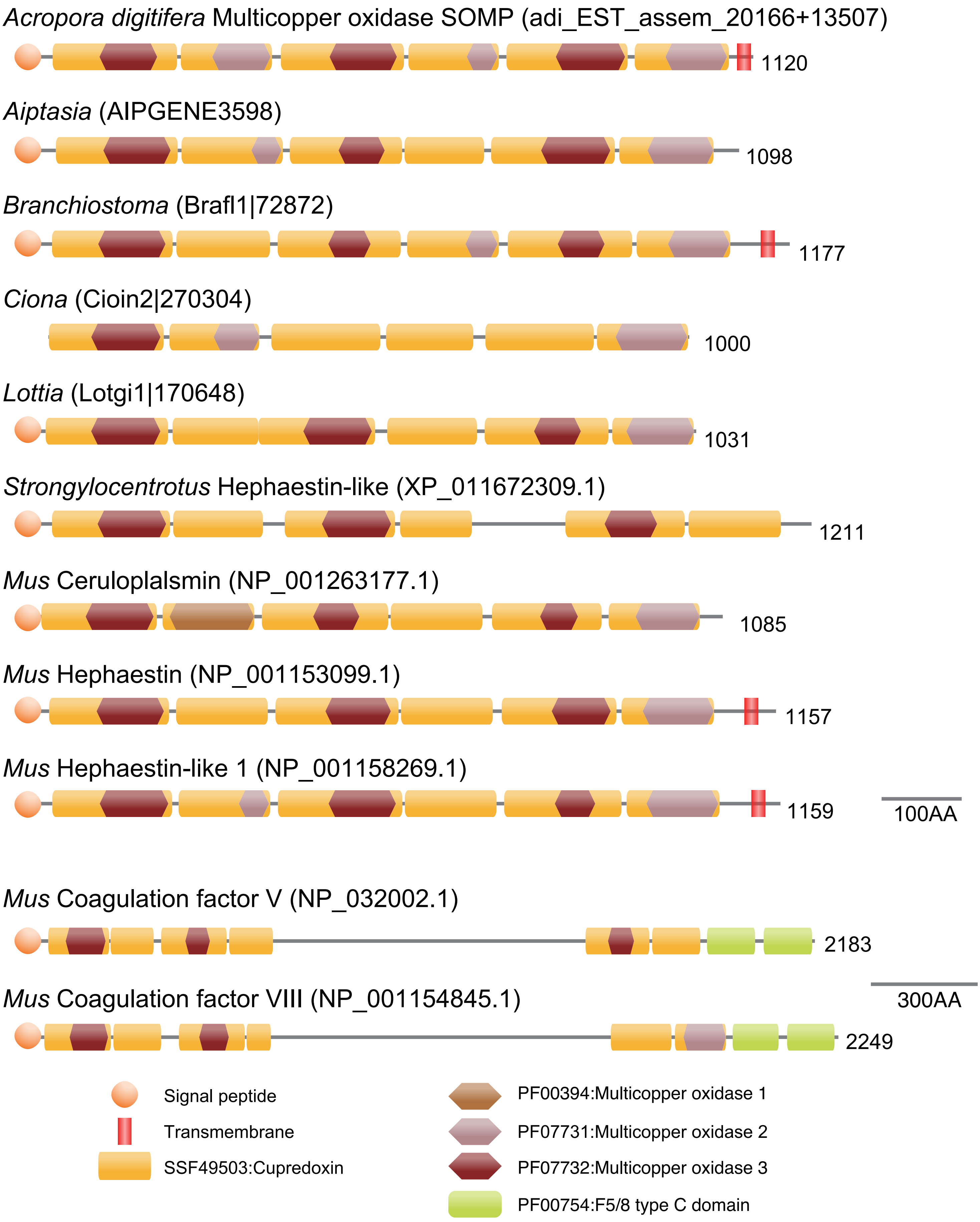

S9 Fig. Six domain multi-copper oxidase architectures of selected animals. *Acropora* multi-copper oxidase SOMP is similar to that of *Aiptasia*. In addition, the domain architecture with six cupredoxin domains is conserved among bilaterian animals. Lengths of amino acid sequences are shown at the right.
