# Supplementary material for: Stepwise Evolution of Coral Biomineralization Revealed with Genome-Wide Proteomics and Transcriptomics
Source: PLoS One. 2016 Jun 2;11(6):e0156424. doi: 10.1371/journal.pone.0156424 (PMC4890752; doi:10.1371/journal.pone.0156424)
Supplement: S10 Fig — Sequences were aligned with MUSCLE, and poorly aligned positions were removed with Gblocks. Then, the resulting dataset (S1 Data) was used for phylogenetic analysis. An ML tree was constructed using the LG + GAMMA model. Cnidarian MCOs are clustered (orange) separately from vertebrate Hephaestin, Hephaestin-like, Ceruloplasmin (green), and Coagulation factors (Cyan). Coral MCO skeletal matrix proteins are highlighted in red. Bootstrap values for the ML analysis are shown at each node. Nodes supported by neighbor-joining (NJ) analysis with high bootstrap support (BS≥70%) are gray-dotted, and nodes supported by NJ (BS≥70%) as well as by Bayesian inference with high posterior probability (≥95%) are black-dotted. The scale bar represents the number of expected substitutions per site in the aligned regions. Abbreviations for protein sequences are presented in S2 Table. (PDF) [file pone.0156424.s011.pdf]

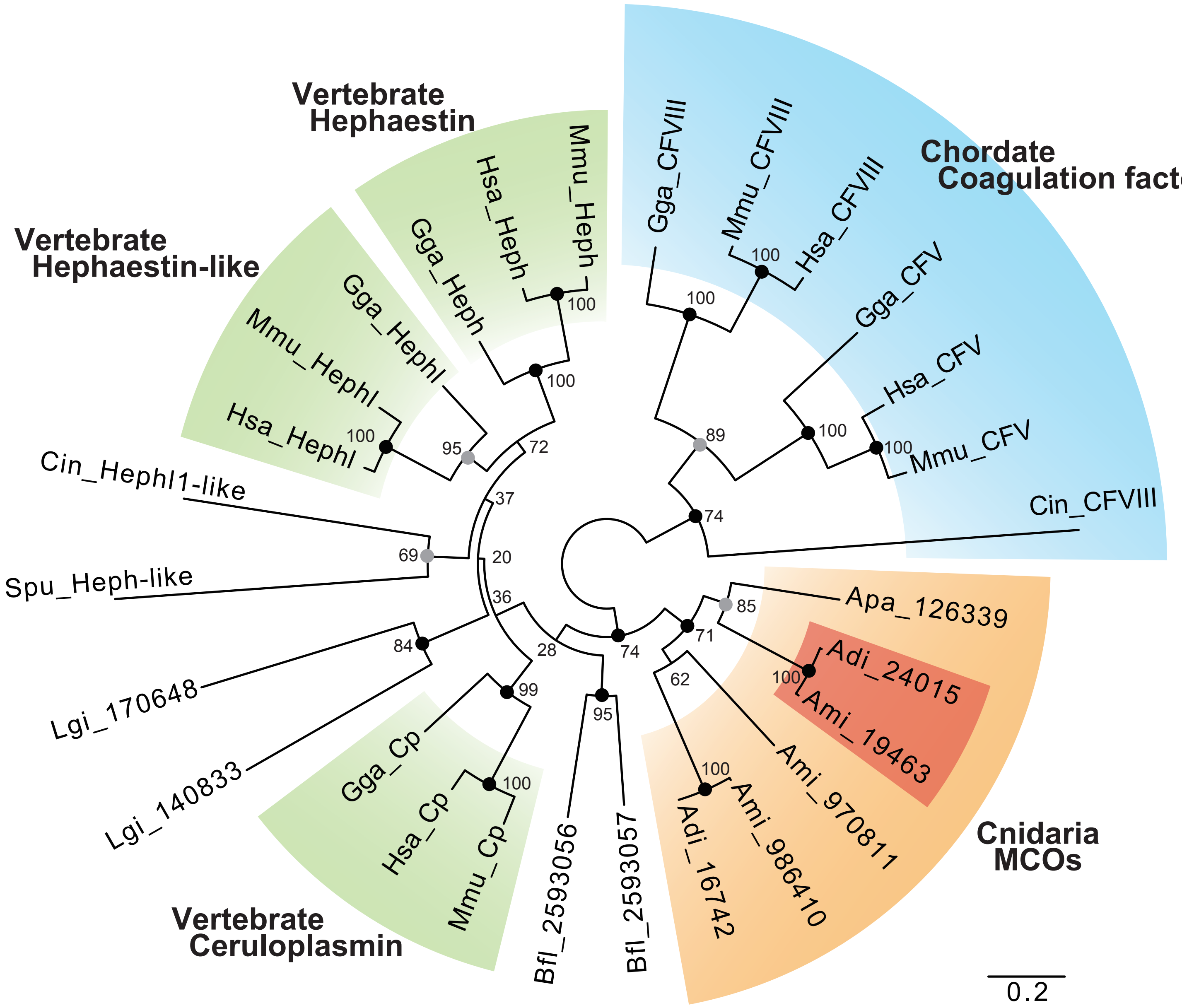

S10 Fig. Maximum likelihood (ML) molecular phylogenetic tree of six-domain multi-copper oxidases (MCOs). Sequences were aligned with MUSCLE, and poorly aligned positions were removed with Gblocks. Then, the resulting dataset (S1 Data) was used for phylogenetic analysis. An ML tree was constructed using the LG + GAMMA model. Cnidarian MCOs are clustered (orange) separately from vertebrate Hephaestin, Hephaestin-like, Ceruloplasmin (green), and Coagulation factors (Cyan). Coral MCO skeletal matrix proteins are highlighted in red. Bootstrap values for the ML analysis are shown at each node. Nodes supported by neighbor-joining (NJ) analysis with high bootstrap support (BS≥70%) are gray-dotted, and nodes supported by NJ (BS≥70%) as well as by Bayesian inference with high posterior probability (≥95%) are black-dotted. The scale bar represents the number of expected substitutions per site in the aligned regions. Abbreviations for protein sequences are presented in S2 Table.
