# Supplementary material for: Stepwise Evolution of Coral Biomineralization Revealed with Genome-Wide Proteomics and Transcriptomics
Source: PLoS One. 2016 Jun 2;11(6):e0156424. doi: 10.1371/journal.pone.0156424 (PMC4890752; doi:10.1371/journal.pone.0156424)
Supplement: S11 Fig — (a) Tandem arrangement of two gene models (aug_v2a.09968 and aug_v2a.09969) encoding MAM domains in the single scaffold of the A. digitifera genome assembly. (b) Sequence alignment of two gene models (aug_v2a.09968 and aug_v2a.09969), termini of which are connected by the sequence deduced from cDNA (adi_EST_assem_4944), indicating that these two gene models may be one gene that encodes a MAM and LDLr domain-containing protein. (PDF) [file pone.0156424.s012.pdf]

a

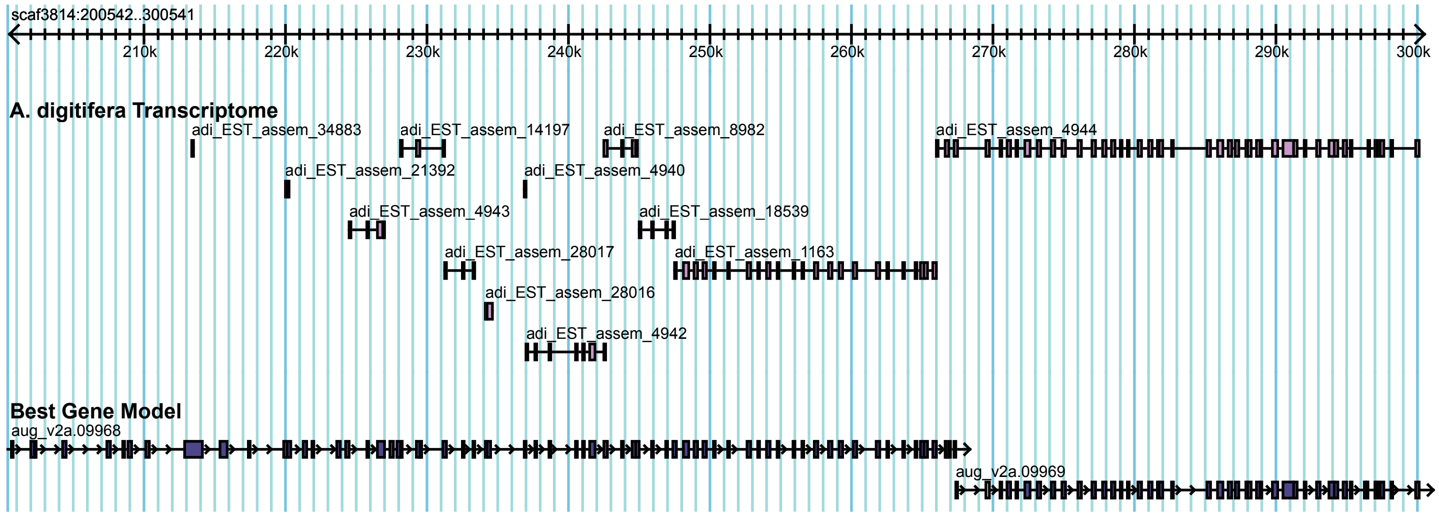

b

```
aug_v2a.09968       3156 TGPPIPGCQPGKFKCANGGNCISVSKVCNFYSDCPGGSDEMNCPATYPLTVHWTIC----
adi_EST_assem_4944     1 --PPIPGCQPGKFKCANGGNCISVSKVCNFYSDCPGGSDEMNCPATCNFQNSFCKWQNAK
aug_v2a.09969            ------------------------------------------------------------

aug_v2a.09968            ------------------------------------------------------------
adi_EST_assem_4944    59 SVDHYDWVRNKGQTPSRYTGPSVDHTTNSSAGYYIYTEVSNRTGFFADAHLVSPLFRQAG
aug_v2a.09969            ------------------------------------------------------------

aug_v2a.09968            ------------------------------------------------------------
adi_EST_assem_4944   119 KSCQFKFWYHMFGPNIGFLQVFYRRNSRDQQLFSVFGNKGNKWNQGTVDIPKCANDFRIV
aug_v2a.09969            ------------------------------------------------------------

aug_v2a.09968            ------------------------------------------------------------
adi_EST_assem_4944   179 IMAKHYSGGALGDIAVDDVSFEQCAESPPSQTCSGISVFRCQSGHCIAMSGKCDFEPDCC
aug_v2a.09969            ------------------------------------------------------------

aug_v2a.09968            ------------------------------------------------------------
adi_EST_assem_4944   239 DGSEETNIVCAKYNRCNFEAGLCDWTQLANDTFNWRMQSGRTGSHRTGPSFDHTTRSRNG
aug_v2a.09969            ------------------------------------------------------------

aug_v2a.09968            -------------------------------
adi_EST_assem_4944   299 RYMYIEASSPRKPGDSAILGSAVFKPTSSCK
aug_v2a.09969          1 --MYIEASSPRKPGDSAILGSAVFKPTSSCK
```

S11 Fig. Gene structure of MAM and LDLr domain-containing proteins. (a) Tandem arrangement of two gene models (aug_v2a.09968 and aug_v2a.09969) encoding MAM domains in the single scaffold of the *A. digitifera* genome assembly. (b) Sequence alignment of two gene models (aug_v2a.09968 and aug_v2a.09969), termini of which are connected by the sequence deduced from cDNA (adi_EST_assem_4944), indicating that these two gene models may be one gene that encodes a MAM and LDLr domain-containing protein.
