# Supplementary material for: Stepwise Evolution of Coral Biomineralization Revealed with Genome-Wide Proteomics and Transcriptomics
Source: PLoS One. 2016 Jun 2;11(6):e0156424. doi: 10.1371/journal.pone.0156424 (PMC4890752; doi:10.1371/journal.pone.0156424)
Supplement: S12 Fig — Lengths of amino acid sequences are shown at the right. (PDF) [file pone.0156424.s013.pdf]

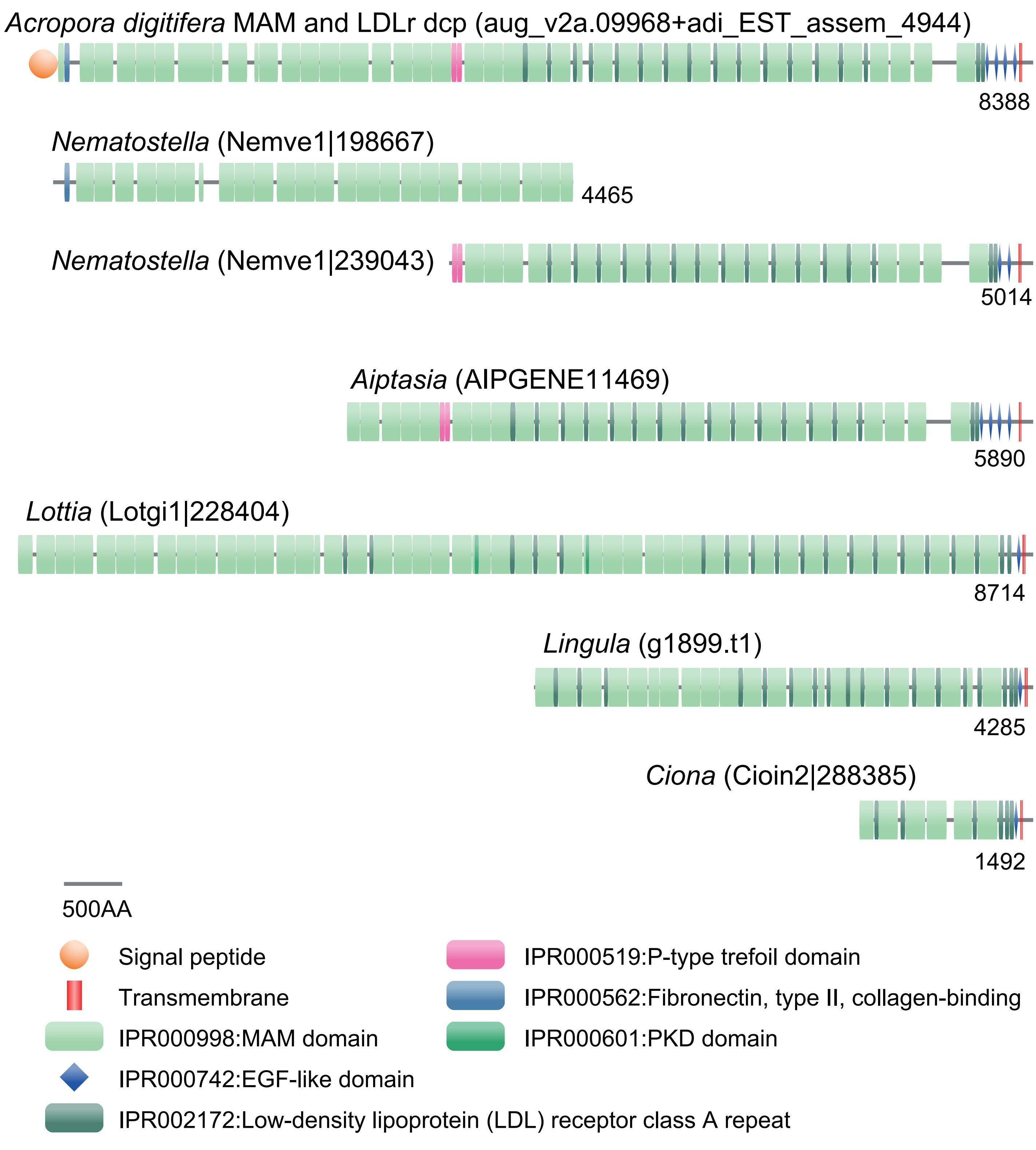

S12 Fig. Domain architecture of MAM and LDLr dcps of metazoan animals. Lengths of amino acid sequences are shown at the right.
