# Supplementary material for: Stepwise Evolution of Coral Biomineralization Revealed with Genome-Wide Proteomics and Transcriptomics
Source: PLoS One. 2016 Jun 2;11(6):e0156424. doi: 10.1371/journal.pone.0156424 (PMC4890752; doi:10.1371/journal.pone.0156424)
Supplement: S13 Fig — This domain architecture is only found in Acropora species. Lengths of amino acid sequences are shown at the right. (PDF) [file pone.0156424.s014.pdf]

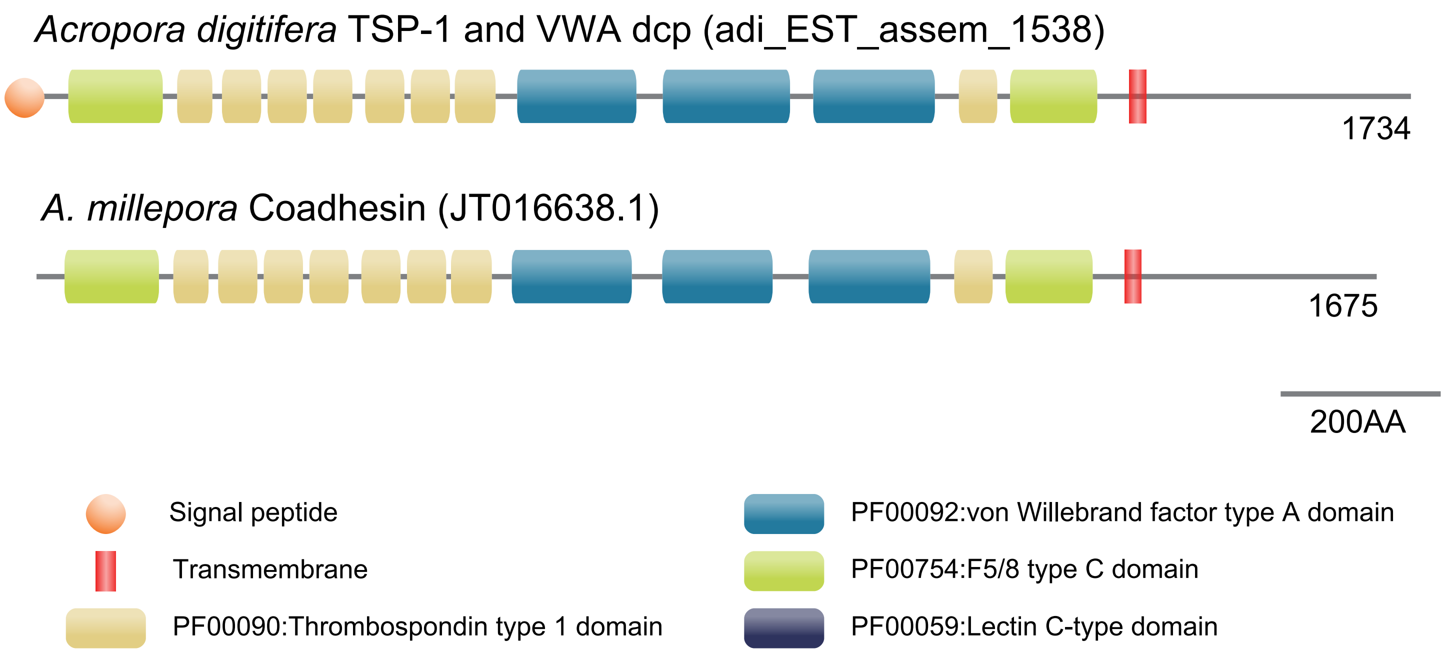

S13 Fig. Coral TSP-1 and VWA domain-containing SOMPs. This domain architecture is only found in *Acropora* species. Lengths of amino acid sequences are shown at the right.
