# Supplementary material for: Stepwise Evolution of Coral Biomineralization Revealed with Genome-Wide Proteomics and Transcriptomics
Source: PLoS One. 2016 Jun 2;11(6):e0156424. doi: 10.1371/journal.pone.0156424 (PMC4890752; doi:10.1371/journal.pone.0156424)
Supplement: S14 Fig — Coral mucin4-like SOMPs contain NIDO, AMOP, VWD, and EGF domains, which are typically present in mucin4 of other animals. In addition, coral mucin4-like proteins have TSP1 domains. Lengths of amino acid sequences are shown at the right. (PDF) [file pone.0156424.s015.pdf]

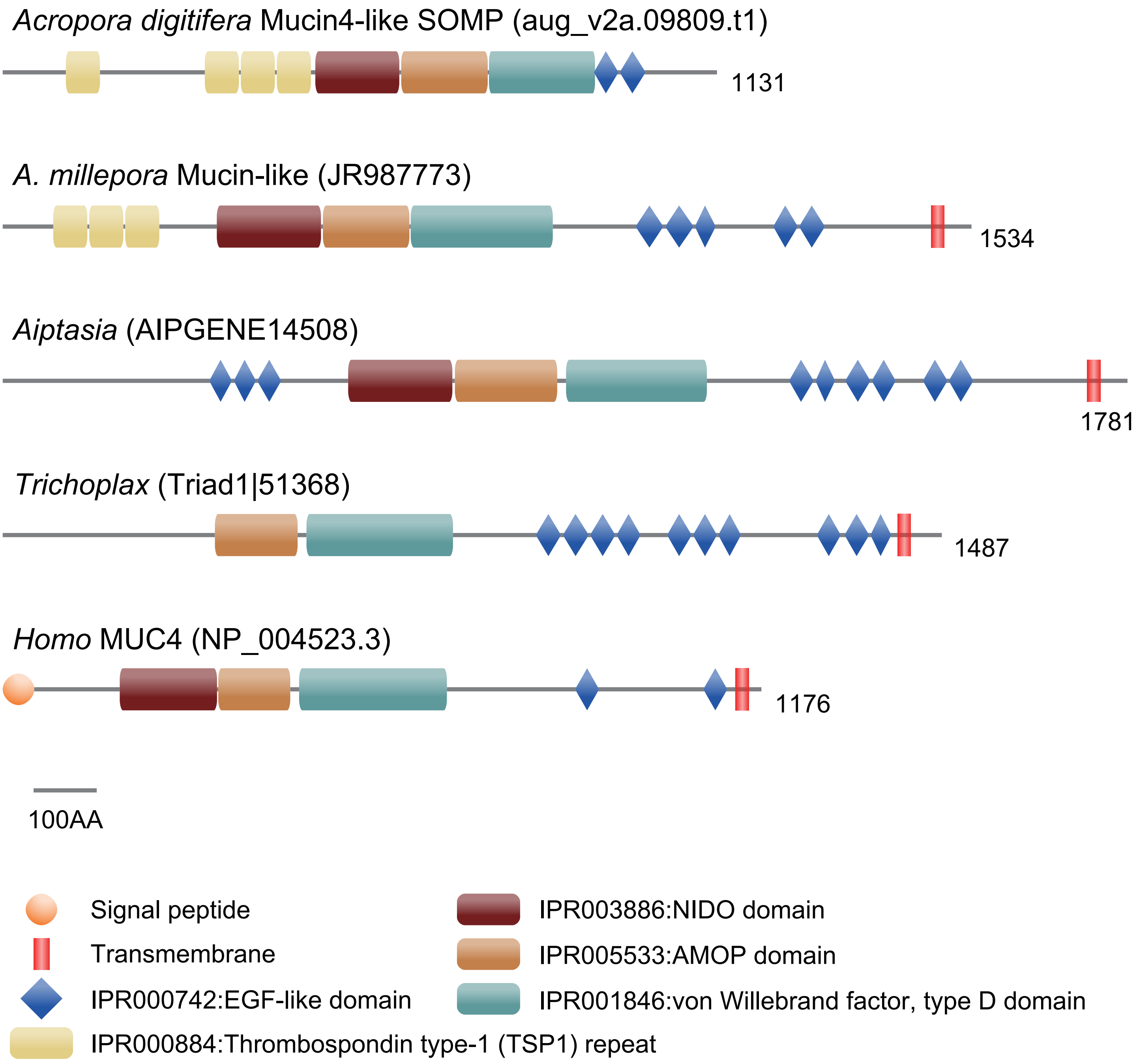

S14 Fig. Domain structure of MUC4 and mucin4-like proteins of representative animals. Coral mucin4-like SOMPs contain NIDO, AMOP, VWD, and EGF domains, which are typically present in mucin4 of other animals. In addition, coral mucin4-like proteins have TSP1 domains. Lengths of amino acid sequences are shown at the right.
