# Supplementary material for: Stepwise Evolution of Coral Biomineralization Revealed with Genome-Wide Proteomics and Transcriptomics
Source: PLoS One. 2016 Jun 2;11(6):e0156424. doi: 10.1371/journal.pone.0156424 (PMC4890752; doi:10.1371/journal.pone.0156424)
Supplement: S15 Fig — A. digitifera CUB dcps and A. millepora CUB dcp have a single CUB domain underlined with red, which is inferred from Adi_CUB dcp sequences with the InterProScan. The threonine-rich SOMP of A. millepora has a conserved sequence in the N-terminus region, while it lacks a CUB domain. Conserved amino acid positions are highlighted with blue. Transcriptome IDs or NCBI accession IDs of the proteins are as follows: A. digitifera CUB dcp-1 N-terminus (adi_EST_assem_9510), CUB dcp-1 C-terminus (adi_EST_assem_5604), CUB dcp-2 N-terminus (adi_EST_assem_30005), CUB dcp-2 C-terminus (adi_EST_assem_21039), A. millepora Threonine-rich protein (JT013896.1), and CUB dcp (JR989025). (PDF) [file pone.0156424.s016.pdf]

```
   Adi_CUB-dcp-1_N   1 MKAFLLSLATLLACIVLTESAP--------YSADVREEAFDALVRSY-LQAVQRDSHMENLTCAECQGVTERNCT
 Ami_Threonin-rich   1 MKAFLLSLATLLACIVLTESAP--------HSADVREEAFDALVRSY-LQAVQRDSHMENLTCAECQGVTERNCT
 Adi_CUB-dcp-2_N/C   1 --MYLFSLTVLSALVLITESIPSVATDFPFFEI--------TYNNDYGILKFQEQEPMENLTCASCEAPSERECT
       Ami_CUB-dcp   1 --MFLFSLTVLSALVLITESIPSVATDFPFFEITKKFDDIETYNNDYGILKFQEQEPMENLTCASCEAPSERECT

   Adi_CUB-dcp-1_N  67 LGERQVQCN--PGEVCTTLEAFNLDTGTTTVTRGCFNITGLNCGDNPGCGALNAT--GNIQSCGQFCCNTSLCNA
 Ami_Threonin-rich  67 LGERQVQCN--PGEVCTTLEAFNLDTGTTTVTRGCFNITGLNCGDNPGCGALNTT--GNIQSCDQFCCNTSLCNA
   Adi_CUB-dcp-2_C  66 LNQTAVVCDQDPNIACLTFEAFNNFTMTTTFRRGCF-LSGIFCEN--ACRSFNASQDGNLTSCVQDCCNSSLCNA
       Ami_CUB-dcp  74 LNQTAVVCDQDPNIACLTFEAFNNFTMTTTFRRGCF-LSGILCEN--ACRSFNASQDGNLTSCVQDCCNSSLCNA

 Adi_CUB-dcp-1_N/C 138 GTL------------------TTVPPQTTDGNTTTEAPTSTEAPTT-----------------------------
 Ami_Threonin-rich 138 GTL------------------TTVTPQTTDGNTTTEAPTSTEPPTNAS---------------------------
   Adi_CUB-dcp-2_C 138 GSLPTEVTTEASTTAKETTATSTTTKQSTAASTTAE-PSTTAAPSTTTKQTTVASTTATTTKPTTAPQTRATTLP
       Ami_CUB-dcp 146 GSLPTEVTTEASTTAQETTATSTTTKQSTGASTTAE-PSTTAAPSTTTKQTTVASTTATTTKPTTAPQTRATTLP

   Adi_CUB-dcp-1_C 166 TPAPTTTPAPTTPFFCNATLSGPSGTFTSPNFPADYPNGVTCVAVANVLANRQLRLSVDFIVLADSGDSLTITVG
 Ami_Threonin-rich 168 TEAPTSTEPPT-----NASTEAPTSTEPPTNASTEAPTTTEAPTTTEAPTTTEAPTTTETPTTTETPTTTAAPTT
   Adi_CUB-dcp-2_C 212 TTAPTTAPAPIA---CGGVLRG-RGTFTSPGFPGNYPNNVRCEWRVFLPRRQAIVFRIVSLDLADPGDSLEFFDS
       Ami_CUB-dcp 220 TTAPTTAPAPIA---CGGVLRG-RGTFTSPGFPGNYPNNVRCEWRVFLPRRQAIVFRIVSLDLADPGDSLEFFDS

   Adi_CUB-dcp-1_C 241 NVVVLLFVG----PLNTVVKRSVDDQSSYESSES-YNNQDYKDYYYFDGRRKEEPYLHVRRK--RQNGNVVTVAG
 Ami_Threonin-rich 238 TET----------PTTTAAPTTTPAPTTTPAPTT--------------------PFF------------------
   Adi_CUB-dcp-2_C 283 GRVIRTFRGLSRRKRSPSHRQTTNEKVLGEGEDGYYDDQEYVDYYYYDGRRKREPYFYQRRKKRRQQDRIVIQGR
       Ami_CUB-dcp 291 GRVIRTFRGLSRRKRSPSHRQTTNEKVLGEGEDGYYDDQEYVDYYYYDGRRKREPYFYQRRKKRRQQDRIVIQGR

   Adi_CUB-dcp-1_C 309 GQTATVTFNSNGSGSASGFSLSFQERAAPEPETESESGSESGGSGSDSGSD
 Ami_Threonin-rich 265 -----------CNATLAGLSGTF---TSPNFQLITQTG-------------
   Adi_CUB-dcp-2_C 358 NQVAGAIFQSDAAGNAAGFSTQFVQ-GAADSETEASAA-----SSESSDED
       Ami_CUB-dcp 366 NQVAGAIFQSDAAGNAAGFSTQFVQ-GAADSESEASAS------SESSDED
```

S15 Fig. Alignment of CUB domain containing SOMPs of *Acropora* species. *A. digitifera* CUB dcps and *A. millepora* CUB dcp have a single CUB domain underlined with red, which is inferred from Adi_CUB dcp sequences with the InterProScan. The threonine-rich SOMP of *A. millepora* has a conserved sequence in the N-terminus region, while it lacks a CUB domain. Conserved amino acid positions are highlighted with blue. Transcriptome IDs or NCBI accession IDs of the proteins are as follows: *A. digitifera* CUB dcp-1 N-terminus (adi_EST_assem_9510), CUB dcp-1 C-terminus (adi_EST_assem_5604), CUB dcp-2 N-terminus (adi_EST_assem_30005), CUB dcp-2 C-terminus (adi_EST_assem_21039), *A. millepora* Threonine-rich protein (JT013896.1), and CUB dcp (JR989025).
