# Supplementary material for: Stepwise Evolution of Coral Biomineralization Revealed with Genome-Wide Proteomics and Transcriptomics
Source: PLoS One. 2016 Jun 2;11(6):e0156424. doi: 10.1371/journal.pone.0156424 (PMC4890752; doi:10.1371/journal.pone.0156424)
Supplement: S16 Fig — The Acropora vitellogenin-like SOMP has a protein kinase domain, which is absent from the other metazoan vitellogenins. Lengths of amino acid sequences are shown at the right. (PDF) [file pone.0156424.s017.pdf]

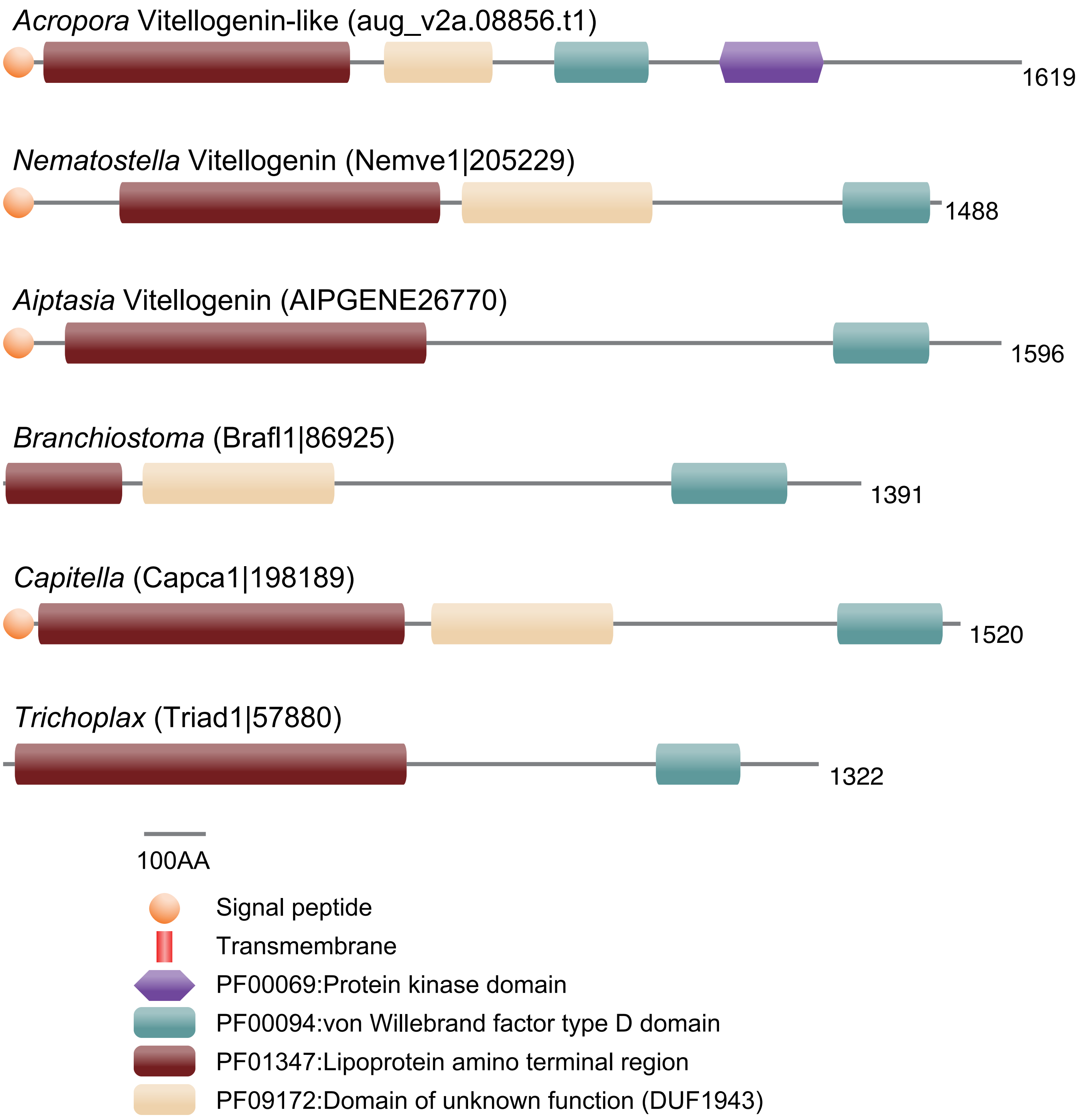

S16 Fig. Vitellogenin domain architectures of selected animals. The *Acropora* vitellogenin-like SOMP has a protein kinase domain, which is absent from the other metazoan vitellogenins. Lengths of amino acid sequences are shown at the right.
