# Supplementary material for: Stepwise Evolution of Coral Biomineralization Revealed with Genome-Wide Proteomics and Transcriptomics
Source: PLoS One. 2016 Jun 2;11(6):e0156424. doi: 10.1371/journal.pone.0156424 (PMC4890752; doi:10.1371/journal.pone.0156424)
Supplement: S17 Fig — Local sequence similarity is found in middle of the proteins. Conserved positions are shaded in blue. Gene model IDs and NCBI accession IDs of the proteins are as follows: A. digitifera EP-like-1 (aug_v2a.22918.t1), EP-like-2 (aug_v2a.00002.t1), A. millepora USOMP-5 (B8VIU6.1), Euphyllia ancora egg protein (AGO04749.1), Galaxea fascicularis egg protein (BAE94663.1), and N. vectensis (Nemve1|248548). (PDF) [file pone.0156424.s018.pdf]

```
Adi_EP-like-1     1 ----------------------------------------MGAARFLVQVTMLLAVKAALSAATNTTRM 29
Adi_EP-like-2     1 ---------------------------------------MCPGSRIFAKIVILLAVDSANSLQANTSTI 30
Ami_USOMP-5       1 ----------------------------------------MGAARFLVQVAIFLLVKPARSAPA-PMWK 28
Ean_EP            1 ------------------------------------------MKIAFVVLGLLMMIAASVA-------- 19
Gfa_EP            1 ------------------------------------------MKTAFVFLGLLLMIVAAVA-------- 19
Nve_248548        1 MQFGRISWKRVVNHFDTIDRTGFEANVENGIYFPNVLSDIHPTGKMYRFLAVLCLVACGYALDLDDQYC 69

Adi_EP-like-1    30 QANPAANTCSQAPTNNCHCQC------------------------------------ELRPDSSTAIAV 62
Adi_EP-like-2    31 HSSPTSK-CSKPASNNCNCNC------------------------------------NVHTSSISDPAL 62
Ami_USOMP-5      29 GNSTARKSCSQASINNCSCRC------------------------------------ELSPASTTANAV 61
Ean_EP           20 --------------NGSGC---------------------------------------------CDKAI 29
Gfa_EP           20 --------------NGSGC---------------------------------------------CDHAI 29
Nve_248548       70 QHQETYYTIYQVPSHNCKCTAKQTGALRYKDGNLQVCNGNEYVNVGGNAGQEAPVGTETNPGKSCRNIL 138

Adi_EP-like-1    63 SA------------------------------------------------------------------- 64
Adi_EP-like-2    63 VT------------------------------------------------------------------- 64
Ami_USOMP-5      62 SA------------------------------------------------------------------- 63
Ean_EP           30 ET------------------------------------------------------------------- 31
Gfa_EP           30 TM------------------------------------------------------------------- 31
Nve_248548      139 TKRKDAKSGMYFIKPRDVVMKTYCYMGTLCGSNGWTMVMKIDGNKNTFRYDSKYWSNKQHYNVAGATGG 207

Adi_EP-like-1    65 ---LQDKIDQL---------------------------------------------------------- 72
Adi_EP-like-2    65 ---LEDKLDHI---------------------------------------------------------- 72
Ami_USOMP-5      64 ---LEDKIDQV---------------------------------------------------------- 71
Ean_EP           32 ---FERKMETL---------------------------------------------------------- 39
Gfa_EP           32 ---FEKKMETL---------------------------------------------------------- 39
Nve_248548      208 FNHVETKLDTYSHFPLSKLCLGLETKGKREWKEINYMAGSLYELIADGKFRETHYGRKFWKSMVPDSSL 276

Adi_EP-like-1    73 ---------------------------IALVNRT-----TPRHSAPVA--------------------- 88
Adi_EP-like-2    73 ---------------------------ISLVNKTGAIPQLPPTLIPAAA-------------------- 94
Ami_USOMP-5      72 ---------------------------IALANRT-----TPRHSAPVA--------------------- 87
Ean_EP           40 ---------------------------IAELKSS---CQSPP--------------------------- 51
Gfa_EP           40 ---------------------------VMELKSS---CRTPP--------------------------- 51
Nve_248548      277 QRNCGREGFNVVKITGSDKRNGFAKVRIGIIGNQENDCHTPDSRIGFGAMGNLCGQNNDNSAGNEARCS 345

Adi_EP-like-1    89 ------------------------------PISSCKEQFDKNNSSPSQVYELTF-GSQVVSVYCHMGNF 126
Adi_EP-like-2    95 -----------------------------DPVFSCKDQFDKDNSSQSKVYELTF-GSQKLPVYCHMGNF 133
Ami_USOMP-5      88 ------------------------------SISSCKEQFDKNNSSPSQVYELTF-GSQVVPVYCHMGNF 125
Ean_EP           52 ------------------------------VASSCQELHEKDPSLESGVYKLVL-GTQKIPVYCHMGNF 89
Gfa_EP           52 ------------------------------VASSCQELHKKDPSLHSGVYELVF-GLQKLPVYCHIGNF 89
Nve_248548      346 PDNGDKTIKSFGYIFAQERQEDALLGSFDKPAKSCKEIVAARPDAKNGVYYLNLRDAPHTRVYCHMTEI 414

Adi_EP-like-1   127 GCGNGGWTLAMKMDGTKTTFHYDSLLWSAQSSYNPAAGKTG-FDMLETKLPTYWSTPFDKVCLGMRVGG 194
Adi_EP-like-2   134 GCGQGGWTLAMKTDGTKSTFHFNSHVWTDKYSFNPEGGKTG-FDSSETKLPTYWSTPFSKVCLGMKRIG 201
Ami_USOMP-5     126 GCGNGGWTLAMKMDGTKTTFHYDSLVWSAQSSYNPAAGKTG-FDMLETKLPTYWSTPFDKVCLGMRLGQ 193
Ean_EP           90 GCGDGGWTPVMKIDGTKLTFLYDSGFWTNKTVYNSEGGMTGFFDLKETMLPSYWTTPLSKICLGMMIHG 158
Gfa_EP           90 GCGDGGWTPVMKIDGKKLTFVYDSGFWSNKTVFNSEGGMTG-FDQKETMLPSYWSTPLSKICLGMMIHG 157
Nve_248548      415 -CGSRGWTIIMKIDGRKTTFKYESSYWKNHNVYNTESIADG-LDNSDMKLHTYSKLPFEKLCLGMRVGG 481

Adi_EP-like-1   195 QQLNFVVLN--MTANSLFSLIADGRYRATSLGRNTWKSLIGTQASLQRNCNREGF--NVRSGYRSNSKA 259
Adi_EP-like-2   202 QKINFIVIR--KNATSLFSLIADGIYRATSLGRDKWKSLIGTDASLQLHCDKEGF--NTVGFRAETAKA 266
Ami_USOMP-5     194 Q-LNFVVLN--MTANSLFSLIADGLYRATSLGRNTWKSLIGAQASLQRNSIEKGS--TP---------- 247
Ean_EP          159 Q-IKFVVLD--KKASSLHSLIADGQYRATSLGRNQWKTLIGSDASLQRNCNKEGF--NPVP--ETWRRV 220
Gfa_EP          158 K-VNYVVIN--KSASSLHSLIADGVYRATSLGRDKWKSLIGSEASLQRNCNKEGF--NPVP--ETWRKT 219
Nve_248548      482 S-LEWLPVNMGKKVDSLFSVIAPGKYVATHISKNEWRKLV-PGSSLQRNCNMQGFNNNPVP-SQPAAKV 547

Adi_EP-like-1   260 RIGIIGNQENNCRSCDSRIGFGTGG--IPDNSNSCGNHAIQS-GDNGRKRIETMGYILVQ          316
Adi_EP-like-2   267 RIGFLGNNQNHCGLCDSRIGFGTGG--TPDDSGTCGNEAADRYSDNGPKSIKTMGYILVQ          324
Ami_USOMP-5     248 -------------------GLVVIG--MPG------------------------------          256
Ean_EP          221 RIGFVANQENDCRTCDSYVGFGSRG------DMSCGNYAAWG-GDNGNRHTTAMGYILVQ          273
Gfa_EP          220 RIGYVANQENNCDTCDSYVGFDSKG------DMSCGNYASYD-ADNGDRRTTTMGYILVQ          272
Nve_248548      548 RIGFVANQENDCGSPDSRIGFGGSGNYCGQSSYSAGNEARCD-GDNGNKSVAGYGYILAQ          606
```

S17 Fig. Sequence alignment of coral egg proteins, EP-like proteins, and a similar protein of *Nematostella*. Local sequence similarity is found in middle of the proteins. Conserved positions are shaded in blue. Gene model IDs and NCBI accession IDs of the proteins are as follows: *A. digitifera* EP-like-1 (aug_v2a.22918.t1), EP-like-2 (aug_v2a.00002.t1), *A. millepora* USOMP-5 (B8VIU6.1), *Euphyllia ancora* egg protein (AGO04749.1), *Galaxea fascicularis* egg protein (BAE94663.1), and *N. vectensis* (Nemve1|248548).
