# Supplementary material for: Stepwise Evolution of Coral Biomineralization Revealed with Genome-Wide Proteomics and Transcriptomics
Source: PLoS One. 2016 Jun 2;11(6):e0156424. doi: 10.1371/journal.pone.0156424 (PMC4890752; doi:10.1371/journal.pone.0156424)
Supplement: S18 Fig — Conserved cysteine residues are colored in yellow. Amino acid positions are shown at the left. (PDF) [file pone.0156424.s019.pdf]

```
  5 CYYCIEDDCETMSLW-INQTCATSQRSLGTSHCGTAAVRYHEGYLGGVPLETTVKGCFDCADKSAACFALAGLLKSSLGW----VVQQCDINCC-----NDTNCNTNVTILS
123 CYECEESDNYTCILTQQSQTCRTSRAALGITHCSSAKVKTRNVLTGAVDV-SFIRGCISCEDKKSACALLAGSFKFRKYA----TMLECDIECC-----NGSYCNDGAASLS
226 CFQCMEDDGLSCSARQQRQICSLDPESLGTTHCGSAVGRKRNQ-NGAIQN-YFYRGCFNCSKKKEACFTLGGYWKGDVNAPGATTLLECELQCCDPNVINGSYCNVETPILK
352 CNVCLEKDETSCSENQQTQVCGIDPYSLGTTHCGSAVGRYRQS-NGDMVY-GFYRGCINCADKMAACAAVGGFRKNVQKW----TQLQCEIECC-----TEDNCNT------
```

S18 Fig. Alignment of repetitive sequences in the Cys-rich SOMP.
Conserved cysteine residues are colored in yellow. Amino acid positions are shown at the left.
