# Supplementary material for: Stepwise Evolution of Coral Biomineralization Revealed with Genome-Wide Proteomics and Transcriptomics
Source: PLoS One. 2016 Jun 2;11(6):e0156424. doi: 10.1371/journal.pone.0156424 (PMC4890752; doi:10.1371/journal.pone.0156424)
Supplement: S19 Fig — (a) Alignment of amino acid sequences of A. digitifera SAARPs. Gene model IDs are as follows: Adi-SAARP1(adi_EST_assem_12928), Adi-SAARP2_N (adi_EST_assem_6252), Adi-SAARP2_C (aug_v2a.01440), and Adi-SAARP3 (adi_EST_assem_995). (b) Genomic positions of gene models aug_v2a.01441 and aug_v2a_01140, consecutively located in the same scaffold, presumably encode one protein Adi-SAARP2. (PDF) [file pone.0156424.s020.pdf]

a

```
 Adi-SAARP1  -MAFVSCFHLRLLFLCLALFMAAECRPDELNKKVDSDETISDDDVSARVQPNGGKIMIVR---------- 59
Adi-SAARP2_N MALITQRVCLALAFFCLALFLV--CLADEERKDDDNTKTIRGKNVSAKIFGRSGKIMIVRVDDDEDDTKD 68
 Adi-SAARP3  -MLAPRLALILLLSSYFGSILITSVECSDEVDMEKKTVKMRGSNTSVLVEGDGGKISTLY---------- 59

 Adi-SAARP1  ------------------DNDYDASDDNDNDDDDNNDNDNDNDDDNDVDRDNDNDDDDFDDSNDDMLSFE 111
Adi-SAARP2_N TVDRVSDKKDNVDDRRDNDDREESTDKKDTVDKKNPIDDKDDKDDKDDVDNDNDKDDDFRDDDEDLLSFE 138
 Adi-SAARP3  ------------------------------------LYFEEDDDDDEDNEESEDEVEDFDD--ENALSFQ 91

 Adi-SAARP1  LDSIEEKDSDGNDVGSTE----GHSVESFKDRPFSLSSVDRNSNALGVAAINVNLSTKLEDSNADVDIML 177
Adi-SAARP2_N LDELKEVDADGDEVDD------KHSVDSFDDVEFQLSHVRTASRFKGLAVISVNLSTHLQNLKANVGIMV 202
 Adi-SAARP3  VESLQEVDESGKPVKASESSEIQHSVSSVKSLSFTVSALQNSTTYQNLTAKTVTLQAQLPNM-ATLELMV 160

 Adi-SAARP1  YLFREDGTVSFGNETFDVQAGTVKFNIKISNWDFCDGSAQDCSEGKA-----GEYLDVNIKFKSK-DTPI 241
Adi-SAARP2_N YLFLEPGSVTFGNETFNVKAGTVKFNIEVNNWDFCQGSSPACSSRKE-----GKFLDLTMKIKSK-DSPT 266
 Adi-SAARP3  VLFLEDGTIKFGNETFKVLSGTMKFNINVTGWQYCDGATVSCLSDSNQPAAVGDNLDLALTVKSEAEDPE 230

 Adi-SAARP1  EVTDEERKSQNKPAVCKDKDTPDTDSDPDDSSDNANDGDDDDDDDCPHIYNMGGDSEMLLNRGVM-NGDT 310
Adi-SAARP2_N EVEDDDR----KKAVCNDKDDDKDK--------------------------------------------- 287
Adi-SAARP2_C --------------------------------XXXDDDDDDDDDDCPIIYSMGGDSEMLLNRGVMLDDDE 38
 Adi-SAARP3  EVDDAKRAETGKDPICVDP-----------------DDPDEEDDDCPVVYDMGGNSEMVLNKGVLVNNMD 283

 Adi-SAARP1  YTAMPFGFPKVEIED-GEKKIKFRVPKFDDNVIIDPSVTPG-RVPKNASPSPALCLKIHILFIAILQAVT 378
Adi-SAARP2_C YTAMPVGFPKLEIED-ETRKFVFRIPKFSKRALVDPSVTPGERTPKLAISAGTW-LQLNFLVTVLVQIAV 106
 Adi-SAARP3  YVAMPQGFPNLEKTGMMQKKLTFRLPKTPGSVIIDPSVNIG-VPPKKQSGSGESIKASSFCFFTLAMLLS 352

 Adi-SAARP1  LFINSW                                                                 384
Adi-SAARP2_C MFVFH-                                                                 111
 Adi-SAARP3  VLIAHF                                                                 358
```

b

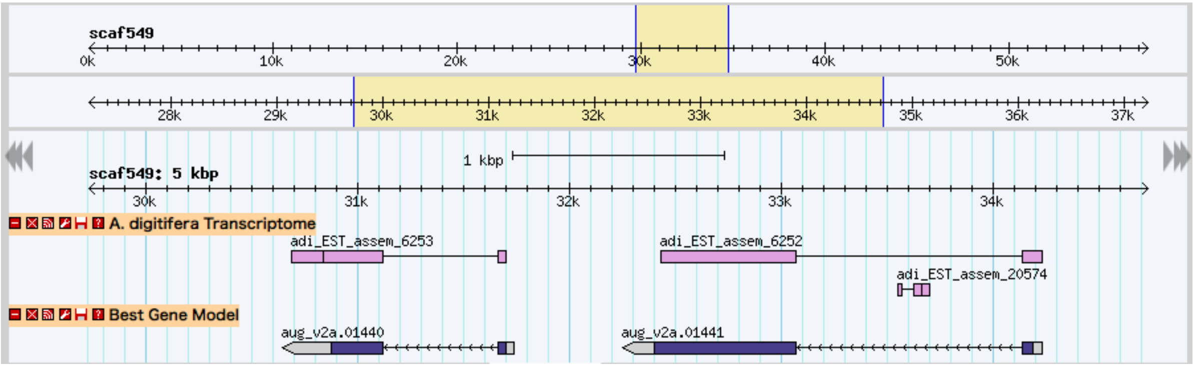

S19 Fig. Amino acid sequences and gene structure of SAARPs. (a) Alignment of amino acid sequences of *A. digitifera* SAARPs. Gene model IDs are as follows: Adi-SAARP1 (adi_EST_assem_12928), Adi-SAARP2_N (adi_EST_assem_6252), Adi-SAARP2_C (aug_v2a.01440), and Adi-SAARP3 (adi_EST_assem_995). (b) Genomic positions of gene models aug_v2a.01441 and aug_v2a_01140, consecutively located in the same scaffold, presumably encode one protein Adi-SAARP2.
