# Supplementary material for: Stepwise Evolution of Coral Biomineralization Revealed with Genome-Wide Proteomics and Transcriptomics
Source: PLoS One. 2016 Jun 2;11(6):e0156424. doi: 10.1371/journal.pone.0156424 (PMC4890752; doi:10.1371/journal.pone.0156424)
Supplement: S21 Fig — Sequence positions are indicated in parentheses. Conserved amino acid positions are highlighted with blue. (PDF) [file pone.0156424.s022.pdf]

```
Adi_SAARP1 (121-240)                  1 DGNDV--------------GSTE----GHSVESFK--DRPFSLSSVDRNSN   31
Ami_SAARP1 (123-242)                  1 DGNDV--------------GSTE----GHSVESFE--DRPFSLSSVDRNSN   31
Adi_SAARP2 (148-265)                  1 DGDEV--------------DD------KHSVDSFD--DVEFQLSHVRTASR   29
Ami_SAARP2 (139-256)                  1 DGDEV--------------DD------KHSVDSFD--DVEFQLSHVRTASR   29
Adi_SAARP3 (101-229)                  1 SGKPV--------------KASESSEIQHSVSSVK--SLSFTVSALQNSTT   35
Ami_Acidic_SOMP (101-229)             1 SGKPV--------------KASKSSEIQHSVSSVG--SLAFTVSALQNSTT   35
Spi_CARP4 (59-177)                    1 DGNDV--------------DTMQ----RHSVDSFD--DVDFTFTKVDTQAK   31
Spi_CARP5 (94-198)                    1 DADDN--------------EVDD----KHSVDSFD--DVKFTFGKVNKKST   31
Spi_integrin_like (15-144)            1 NGNVV--------------GLTSQK--KHSWLSSENPNQKFTFSPLNNFTT   35
Nve_240217 (48-185)                   1 NGSGVILDFDSVAEYNAAGQLVDGG--RRNFQNFE--KLDFKFYGDRD-AT   46
Nve_246686 (75-195)                   1 NGNPV--------------GQAGSV--KHNFNNFA--QLDFQLSDVVQ-DK   32
Nve_215658 (1-79)                       ---------------------------------------------------
Hma_232959 (295-428)                  1 RGKVLDIKIYSVEEVDSLGNKIEEK--GHFIRNFS--NQFFNISQVSNALK   47
Cgi_011423598 (167-290)               1 DGTEI--------------GKSGQQ--THTFNTFA--NLDFSFSQIID-DR   32
Cgi_011442506 (69-189)                1 -GNTV--------------GTSGSD--KHSFNNFA--QLSFEISSLQD-TM   31
Cgi_011442493_C (361-484)             1 SGNPV--------------GASGAM--KHSFNTFA--SLDFTFSALED-TS   32
Cgi_011423636 (421-544)               1 NGNNV--------------GTSGRF--KHIFNNFA--GQKFDISELTD-DT   32
Cgi_011442493_N (97-220)              1 DGAVI--------------GKGGRV--KHSFNTFA--SQSFMFSNITD-DQ   32
pfu_aug1.0_25886.1_69736.t1 (65-206)  1 DDDERRITLEYESIQEFDQSGTGINLRDHYLRNFA--GRDFSFGTTRE-QL   48

Adi_SAARP1 (121-240)                 32 ALGVAAINVNLSTKLEDS-NADVDIMLYLFREDGTVSF-GNETFDVQAGTV   80
Ami_SAARP1 (123-242)                 32 ALGVAAINVNLSTKLEDS-NADVDIMLYLFREDGTISF-GNETFDVQAGTV   80
Adi_SAARP2 (148-265)                 30 FKGLAVISVNLSTHLQNL-KANVGIMVYLFLEPGSVTF-GNETFNVKAGTV   78
Ami_SAARP2 (139-256)                 30 FKGLAVISVNLSTHLQNN-KANVGIMVYLFLEPGSVTF-GNETFNVKAGTV   78
Adi_SAARP3 (101-229)                 36 YQNLTAKTVTLQAQLPNM--ATLELMVVLFLEDGTIKF-GNETFKVLSGTM   83
Ami_Acidic_SOMP (101-229)            36 YQNLSAKTVTLQAQLPNM--ATLELMVVLFLEDGTIKF-GNETFKVLSGTM   83
Spi_CARP4 (59-177)                   32 YDGLPVTNVNLSATLPSS--SSLEIMVYLFRRAGKVTF-GNETFRVEKGTI   79
Spi_CARP5 (94-198)                   32 LDGIHVTTVNLSTYLDDQ-KASLEIIVYLFHEAGSVRF-------------   68
Spi_integrin_like (15-144)           36 FQRLPVKRINLSVSLAGP-QANLEIQVLLFLKSGKIEF-GNETFNVRSGTF   84
Nve_240217 (48-185)                  47 FQNISCRTFSMEAKIPST-NATFRVQLFIFKENGTYEW-GDEAIDVTRGAL   95
Nve_246686 (75-195)                  33 FQDISVYTFKMTANITSV-KATFMSQVYIFAESGSYVW-GDEETEVKKGSV   81
Nve_215658 (1-79)                     1 ----------MTSYISSV-KANFMSQVYIFAENGSYVW-GDEETEVRKGSV   39
Hma_232959 (295-428)                 48 Y-GLFATVFNFTLFFG---NASISMEFIIFNSSGNILVESGETYHIVPGTM   94
Cgi_011423598 (167-290)              33 YMNLTSKRVDFSAKFPST-GGRLVVQVFVFTQAGEISV-DGERTAVTAGTV   81
Cgi_011442506 (69-189)               32 YENLTTKRINFTASIADV-SATLQVQTYIFTEAGNITV-GGEITKVEKGTV   80
Cgi_011442493_C (361-484)            33 FAGITAKRLNFTANIESV-NATLTVPVYIFTNGGVISI-DSEKSTVTAGTI   81
Cgi_011423636 (421-544)              33 YQNLAVKRLDCTAYLDTV-GARLAVQIYLFREEGSVSQ-GDEESRVSKGTL   81
Cgi_011442493_N (97-220)             33 YQNLSAKRLDFVANLTSV-GATLTAQMYLFEEEGNITQ-DDEVSQVSKGTL   81
pfu_aug1.0_25886.1_69736.t1 (65-206) 49 FQNLTTRSFDFQGRIQDTFNAQLVSNVYIFRDSGNVTN-GNETFPVRRGNI   98

Adi_SAARP1 (121-240)                 81 KFNIKISNWDFCDG--SAQDCSEG-----KAGEYLDVNIKFKS-KDTP     120
Ami_SAARP1 (123-242)                 81 KFNIKISNWDFCDG--SAQDCSEA-----KAGEYLDVNIKFKS-KDTP     120
Adi_SAARP2 (148-265)                 79 KFNIEVNNWDFCQG--SSPACSSR-----KEGKFLDLTMKIKS-KDSP     118
Ami_SAARP2 (139-256)                 79 KFNIEVNNWDFCEG--SSPACSSR-----KEGKFLDLTMKIKS-KDSP     118
Adi_SAARP3 (101-229)                 84 KFNINVTGWQYCDG--ATVSCLSDSNQPAAVGDNLDLALTVKSEAEDP     129
Ami_Acidic_SOMP (101-229)            84 KFNINVTGWQYCDG--ATVSCLSDSNQPAAVGDNLDLALTVKSEAEDP     129
Spi_CARP4 (59-177)                   80 KFNIRISNWDFCDG--SRFDCDEG-----KIGEFLDLKLKIKS-KDSP     119
Spi_CARP5 (94-198)                   69 -----ISNWNFCDGDESSGECSES-----KIGEFLDLSLKIKS-KGSP     105
Spi_integrin_like (15-144)           85 KFNIKVSDWQFCGT--NAEVCKNSTTGANEIGQFLDIGMSIGSVAEEP     130
Nve_240217 (48-185)                  96 KFNIEVEDWKFCGS--GNLTCRRSGQN--EVGEYLEVVICIKG-RKSP     138
Nve_246686 (75-195)                  82 KFNIRVEGWKFCGE--PGFTCNDG------VGSYLDSKICIASKKGSG     121
Nve_215658 (1-79)                    40 KFNIKVEGWKFCGD--QGYTCKEG------VGSYLDSKICIASKKGSG     79
Hma_232959 (295-428)                 95 KFNIVIRDWIFCGT--QKAACKTG-----TIGSYLDLQLEMKG-LLCV     134
Cgi_011423598 (167-290)              82 KFNILVESWGFCGY--NGITCTKGNVE--QQGEAIDFTISVKG-KGDQ     124
Cgi_011442506 (69-189)               81 KFNIILDGWTFC----TASTCNKGGSG--EVGAFVDLTISIKG-KGTP     121
Cgi_011442493_C (361-484)            82 KFNIQIEGWQFCGN--SGVTCKQGSKD--EIGDAIEFVITIKG-KGTQ     124
Cgi_011423636 (421-544)              82 KFNVFIENWKFCGS--DGMECRKGKKS--EFGEFIDFALSIKG-SKPP     124
Cgi_011442493_N (97-220)             82 KFNIVIENWSFCGM--NGETCKQGPKT--SVGEYIEFVIAIKG-KKSG     124
pfu_aug1.0_25886.1_69736.t1 (65-206) 99 KFGFDITNWRFCGGD-GGATCRMGNRQ--YEGAGIDLTLRIRG-PRVP     142
```

S21 Fig. Alignment of non-acidic, conserved sequences of coral SAARPs, CARPs, and other metazoan proteins. Sequence positions are indicated in parentheses. Conserved amino acid positions are highlighted with blue.
