# Supplementary material for: Stepwise Evolution of Coral Biomineralization Revealed with Genome-Wide Proteomics and Transcriptomics
Source: PLoS One. 2016 Jun 2;11(6):e0156424. doi: 10.1371/journal.pone.0156424 (PMC4890752; doi:10.1371/journal.pone.0156424)
Supplement: S22 Fig — Conserved positions are shaded in blue. Gene model IDs or transcriptome IDs of the proteins are as follows: A. digitifera USOMP-1a (aug_v2a.21723.t1), USOMP-1b (aug_v2a.02662.t1), USOMP-1c (aug_v2a.02663.t1), USOMP-9 (aug_v2a.20893), and A. millepora USOMP-1 (JT021412.1). (PDF) [file pone.0156424.s023.pdf]

```
Adi_USOMP-1a        --------------------------------------------------------------------------
Adi_USOMP-1b      1 MMEDKKDLEKKMDLP----DSKLKEPTRTKRLLFVGALTLFLLLGVVVSFALFLTLKETVAAPSKDRLVDVNLR
Adi_USOMP-1c      1 -MEDKKDLEKKMDLP----DSKLKESSRRKKLLLVGALTLFLLLGVVVSFALFLTLNETAAAPSKDRLVDVNLR
Adi_USOMP-9       1 -MDATKDPEPKVQFSEKANDSLKKPCRRKTKIVALGGITLVLLLCAVVSVSVYFLLKKNV--PHTENLVEVDLE
Ami_USOMP-1         --------------------------------------------------------------------------

Adi_USOMP-1a      1 --------------------------MVICVTGVLRRT----------------------VVGDSHPDDHF---
Adi_USOMP-1b     71 EGDSLTYRVDQDIETNTGAFTHKDKSVIIVGIQVLNKSSQEYWFVVNFNVSQ-----DGKIKEDSMSVDYYLVR
Adi_USOMP-1c     70 EGDSLTYRVDQDIETNTGAFTHKDKNVIIVGIQVLNKSSEEYWFVVNFNVSQ-----DGKIKEDSMSVDYYLVR
Adi_USOMP-9      72 EGETLTYLVDHDINVQGGG-VQKTAIKVTVGFRVLNKTSEEYWFLTKFNFSRVQLEGNMDIGKIKSLTEYFVVR
Ami_USOMP-1         --------------------------------------------------------------------------

Adi_USOMP-1a     24 ---PSRSPGESFEVHGESSADDQQLRLVFSVLQFLLPAVKRDLYENIDGNRNATVSAEDSPLLPANVMMHREAD
Adi_USOMP-1b    140 LRMNSSLPGESLEVYGENSTDDQQLRLVFSVLQLLLPAVKRDLYENIDGNRNATVSAEDSPLLPANVIMHREAN
Adi_USOMP-1c    139 LNMNSRSPGESFEVHGESSADDQQLRLVFSVLQFLLPAVKRDLYENIDGNRNATVSAEDSPLLPANVMMHREAD
Adi_USOMP-9     145 LLTHSRKTTFPFEVHGDRHTNIELIRLVYGILVQLIPSLKRDLYECVDGQKAHLLDTEESPLLPGSVKMHREAN
Ami_USOMP-1         --------------------------------------------------------------------------

Adi_USOMP-1a     95 TSGKDAVSIKNHFNGADMVGMLSDLDMEFTYSDQSSIKKSNGMVSEGHAYFSQQLNFETPIRTENGTEISMIKM
Adi_USOMP-1b    214 TSGKDAVSIKNHFNGADMVGMLSDLDMEFTYSDQSSIKKSNGMVSEGHAYFSQQLNFETPIRTENGTEISMIKM
Adi_USOMP-1c    213 TSGKDAVSIKNHFNGADMVGMVSDLDMEFTYSDQSSIKKSNGMVSEGHAYFSQQLNFETPIRTENGIEISMIKM
Adi_USOMP-9     219 TTDKDRVTIKNHFDRTDFDDKLSDIDLDLKYSDIAVINKSNGMVSESHVHFYERLNFGEPI-DNVGFKVTNMQV
Ami_USOMP-1       1 --------------------------------------KSNGMVSEGHAYFSQQLNFETPIRTENGTEISMIKM

Adi_USOMP-1a    169 TVKSRVLLIGTEALIYPSPESIDFQGLFVKLFLSKP-----------SPPVLSLNETTDAGQFSLNDTNEDPF-
Adi_USOMP-1b    288 TVKSRVLLIGTVALIYPSPESIDFQGLFVKLFLSKP-----------SPPVLSLNETTDAGQFPLNDTNEDPF-
Adi_USOMP-1c    287 TVKSRVLLVGTEAFIYPSPESIDFQGLFVKLFLSKP-----------SPPVLSLNETTDAGQFSLNDTNEDPF-
Adi_USOMP-9     292 TVDSHASLIESNFLGFEESKTVDLHS-FVRLVISNTNFTFSGVNNTETPTVVEPNTVSNSSSSPFNTSSPSQHL
Ami_USOMP-1      37 TVKSRVLLXGTVALIYPSPESIDFQGLFVKLFLSKP-----------SPPVLSLNETTDAGQFSLNDTNEDPF-

Adi_USOMP-1a    231 --------APLSRSRRAVSNSDNANASLVSEILERIGPVCLFFDRQFQLYSLNVNSVNLTLSASVSVQIDGGNT
Adi_USOMP-1b    350 --------APLSRSRRAVSNSDNANVSLVSEILERIGPVCLFFDRQFQLYSLNVNSVNLTVSASVSVQIDGANT
Adi_USOMP-1c    349 --------APLSRSRRAVSNSDNANASLVSEILERIGPVCLFFDRQFQLYSLNVNSVNLTVSARVSVQIDGANM
Adi_USOMP-9     365 NLTGNFSGTPLERSRRA--------GNVVP--WNEAGHLSLNTERSFTLFEKKVIGINIKGEGKIWLK--DGRP
Ami_USOMP-1      99 --------APLSRSRRAVSNSXNANASLVSEILERIGPVCLFFDRQFQLYSLNVNSVNLTLSASVSVQIDGPHT

Adi_USOMP-1a    297 SRIDVSLVLSV-GQNLTSVVIQKFVRMVSLQELSDVNLNFPPIFRFLRGSTSFLESNTDVSGRLVVLARFRLSL
Adi_USOMP-1b    416 SRIDVSLVLSV-GKNLTWFVIQKFVRMVSLQELSDVSLNLPPIFRRLRGSTSFLESNTDVSGRLVVFPGFRLSL
Adi_USOMP-1c    415 SRIDVSLVLSV-GQNLTSVVIKKFVRMVSLQELSDVNLNFPPIIRFLRGSTSFLESNTVVSGRLIVVTGFRLSL
Adi_USOMP-9     427 AELGVGVYLFIGGRRVDGNLLTKQYKGNQLRDGIGLRIEYTRSIPVITIRKPVFILLLGIDFRLIGAFGIELNF
Ami_USOMP-1     165 SRIDVSLVLSV-GQNLTSVVIQKFVRMVSLQELSDVNLNFPPIFRFLRGSTSFLESNTDVRGRLVVLARFRLSL

Adi_USOMP-1a    370 PLQNNSVDPPRLNLK-----------------------------------------------------------
Adi_USOMP-1b    489 PLQSNSVDPPRLNLK-----------------------------------------------------------
Adi_USOMP-1c    488 PLQNNSVDPPRLNLKG----------------------------------------------------------
Adi_USOMP-9     501 PWDGNSVSPVKLAVEMEPNAEVTLSIHAYVSAYFIRVGVYGDGTLVRVGLPVTLSYQATRFSGEKWCLDLSARL
Ami_USOMP-1     238 PLQNNSVDPPRLNLKIEPYAVIVVRRLIVA----MSVBXIQQXVXARXVVXXSGPKVTLSFNDDQLCVTVSDRV

Adi_USOMP-1b    504 -----------------------------KLWLL----------------------------------------
Adi_USOMP-1c        --------------------------------------------------------------------------
Adi_USOMP-9     575 TALELSAGIFYQWRGWWFRWGTRHTLYEFGRWAAINRNWRLLALCG----------------------------
Ami_USOMP-1     308 IGPDVPVTFFRRLR------VCRRIPRVGRLWVRTRRGWRLRRIFTFSRRCFWVIISGFRGRLSPTVTQEGFVR

Ami_USOMP-1     376 VCNITKAANPSILLPTPTSQIAQSISTAQMVSSTSASIFATPVLALQSSSLRISPASTAPTSATVSSPVASIS
```

S22 Fig. Sequence alignment of the Adi-USOMP-1 family and USOMP-9. Conserved positions are shaded in blue. Gene model IDs or transcriptome IDs of the proteins are as follows: *A. digitifera* USOMP-1a (aug_v2a.21723.t1), USOMP-1b (aug_v2a.02662.t1), USOMP-1c (aug_v2a.02663.t1), USOMP-9 (aug_v2a.20893), and *A. millepora* USOMP-1 (JT021412.1).
