# Supplementary material for: Stepwise Evolution of Coral Biomineralization Revealed with Genome-Wide Proteomics and Transcriptomics
Source: PLoS One. 2016 Jun 2;11(6):e0156424. doi: 10.1371/journal.pone.0156424 (PMC4890752; doi:10.1371/journal.pone.0156424)
Supplement: S23 Fig — (PDF) [file pone.0156424.s024.pdf]

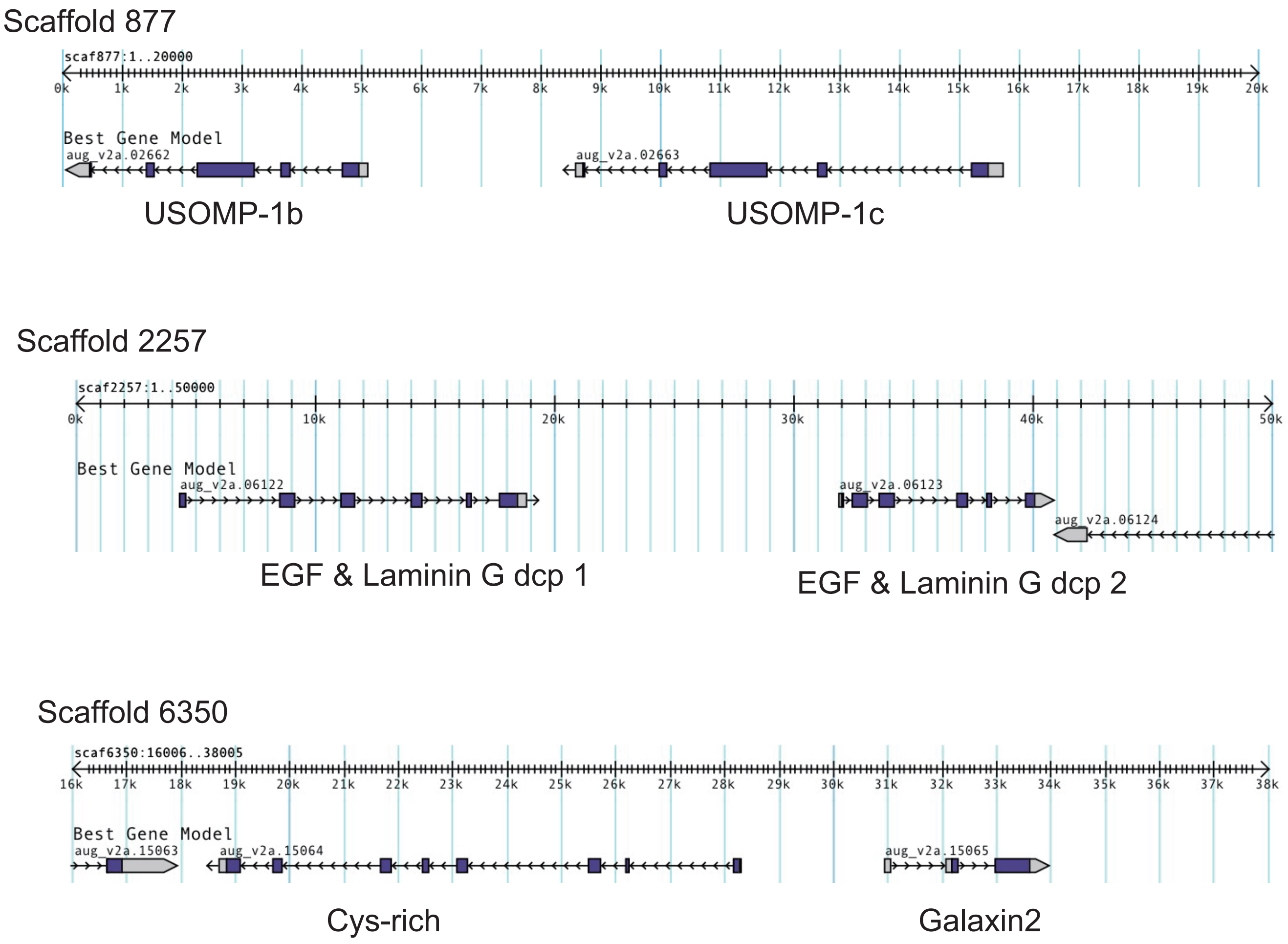

S23 Fig. Tandem arrangement of SOMP genes in the *A. digitifera* genome scaffold.
